# Small-scale screening of novel biobased monomers: the curious case of 1,3-cyclopentanediol†

**DOI:** 10.1039/c8ra08811j

**Published:** 2018-11-29

**Authors:** G. J. Noordzij, C. H. J. T. Dietz, N. Leoné, C. H. R. M. Wilsens, S. Rastogi

**Affiliations:** Chemelot InSciTe Urmonderbaan 20F NL-6167 RD Geleen The Netherlands; Aachen-Maastricht Institute of Biobased Materials (AMIBM), Faculty of Science and Engineering, Maastricht University, Brightlands Chemelot Campus 6167 RD Geleen The Netherlands karel.wilsens@maastrichtuniversity.nl; Inorganic Membranes and Membrane Reactors, Dept. Chemical Engineering and Chemistry, Eindhoven University of Technology PO Box 513 Eindhoven The Netherlands

## Abstract

In this work, we report on the small scale polycondensation and consecutive analysis of novel polyesters based on the potentially renewable 1,3-cyclopentanediol (CPdiol). To avoid evaporation of monomers during thin-film polymerization reactions, trimer pre-polyesters have been synthesized from the corresponding acid-chlorides with diol monomers. Polymerization of these trimers was explored by thermogravimetric analysis to identify potential side reactions, and to assess the ideal polymerization temperature. In general we observe that *trans*-1,3-cyclopentanediol exhibits good thermal stability up to 200 °C, whereas thermal dehydration of the alcohol end-groups occurs upon further heating. In contrast, for *cis*-1,3-cyclopentanediol, the ester bonds of the cyclopentane end-groups become labile, thereby generating carboxylic acid end-groups, and 3-cyclopentenol already at 180 °C. The thermal dehydration reactions yield double bond end-groups, which in turn facilitate cross-linking through cross-coupling and Diels–Alder reactions, leading to an increase in molecular weight. Despite the limited thermal stability of CPdiol, here we demonstrate that polymerization of CPdiol can successfully be achieved in thin-film polycondensation conditions at 180 °C, yielding molecular weights well above 10 kg mol^−1^.

## Abstract 2

In this work, we report on the small scale polycondensation and consecutive analysis of novel polyesters based on the potentially renewable 1,3-cyclopentanediol (CPdiol).

## Graphical abstract

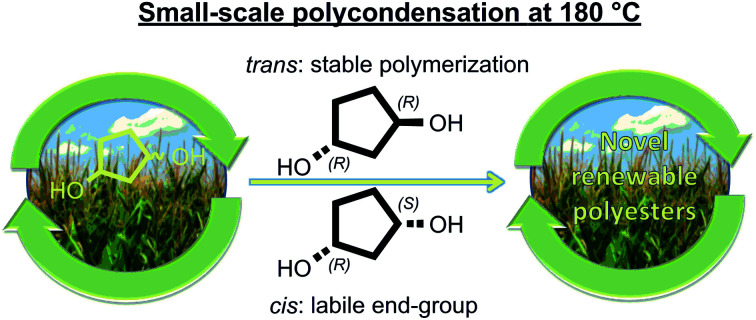

## Introduction

Worldwide, strenuous efforts are taking place to replace oil-based monomers with renewable biomass-based monomers, for both “drop-in” purposes and as new monomers.^1–5^ However, in particular for new building blocks, it is not assured that these monomers – or the resulting polymeric materials – will function as desired. In order to fully assess such monomers and polymers, generally large amounts (*e.g.* several kilos) are required to conduct repeatable processing and characterization tests. Unfortunately, academic research often leads to new monomers obtained *via e.g.* new (bio)catalytic processes having yields in gram scale, or lower. Another major limitation for translations of lab scale synthesis to large scale are the required revenues: significant investment in both time and money is needed to develop new monomers and polymers on a larger scale. To overcome this mismatch in scale, a viable assessment method is desired to polymerize and qualify monomers on a small scale.

High-throughput screening equipment is a well-known tool in the fast screening of molecules in *e.g.* organic synthesis, drug-discovery, and cell-experiments. Also in the field of polymer synthesis, semi-automated high-throughput techniques have been used in solution polymerization,^6,7^ olefin polymerization,^8^ and melt-polycondensation.^9,10^ For example, a small-scale high-throughput melt-polycondensation experiment has been described by Gruter *et al.*^11^ for the evaluation of catalyst effectivity in the synthesis of poly(ethylene 2,5-furandicarboxylate) (PEF), commonly considered to be the renewable replacement of poly(ethylene terephthalate) (PET). In general, such screening techniques can lead to faster catalyst development, but can also provide systematic identification of structure–property relation in polymers by varying monomer compositions. An added benefit of small-scale screening experiments is a minimal requirement of monomer availability, making this an attractive technique for assessing the potential of new building blocks. Despite the advantages of high-throughput experimentation, full automation for polymerization reactions can still be challenging due to the time restrictions for polymer characterization. For example, important polymer characterization techniques such as gel-permeating chromatography (GPC) or differential scanning calorimetry (DSC) require relatively long analysis times, potentially becoming the bottleneck in high-throughput screening processes.

**Scheme 1:**
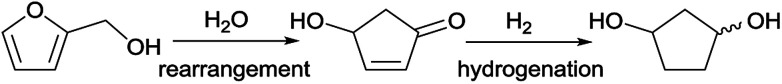
Reaction pathway for the synthesis of 1,3-cyclopentanediol from renewable furfuryl alcohol.^12^

The group of Zhang *et al.*^12^ has developed a new industrially scalable and cost-effective synthesis route to 1,3-cyclopentanediol (CPdiol) from biomass. These authors reported that CPdiol, usually obtained from non-renewable cyclopentadiene, could be obtained from the aqueous phase rearrangement of furfuryl alcohol, followed by hydrogenation over RANEY® (Scheme 1). CPdiol was obtained as a mixture of *cis* and *trans* isomers, which could be further purified *via* fractional distillation under vacuum.

Typically in racemic cycloaliphatic monomers the *trans* isomer results in higher rigidity in the polymer, resulting in an increase in glass transition temperature (*T*_g_). Higher symmetry of the *trans* isomer also results into more perfect crystals, favoring higher crystallinity, as opposed to the *cis* isomer. Indeed, this effect is well described for polyesters having cycloaliphatic rings, for example 1,4-cyclohexanediol (CHdiol),^13,14^ 1,4-cyclohexanedimethanol (CHdm),^15^ and others.^14,16–19^ In most cases the polyesters with a high *trans* ratio are semi-crystalline, whereas crystallinity is often lost when reaching, or exceeding, a 50/50 *cis*/*trans* mixture. With this literature precedent we expect that the properties of polymers with CPdiol will vary with the *cis*/*trans* ratio. Additionally, compared to six-membered rings, the five membered cyclo-aliphatic ring in CPdiol is expected to suppress rigidity and symmetry.^20^ This is due to the steric configuration of the cyclopentane ring, which cannot adopt the well-known ‘boat’-conformation found in cyclohexane.^21^ These characteristic differences make CPdiol an interesting candidate for polymerization in order to provide a chemical and physical comparison with CHdiol and CHdm.

Zhang *et al.*^12^ polymerized 1,3-CPdiol into polyurethanes, and several (enzymatic) transesterification reactions involving various 1,2- and 1,3-CPdiol structures are known.^22–24^ However, to the best of our knowledge, polyesters having 1,3-CPdiol have not been reported in literature. This might be attributed to the fact that CPdiol is only available on small scale.

To overcome the challenges of the limited availability of CPdiol, and to investigate the physical properties of polyesters with CPdiol compared to polymers having cyclohexane counterparts, a small-scale polymerization screening method is reported in this publication.

## Experimental section

### Materials

1,3-Cyclopentanediol (15/85 *cis*/*trans*), 1,4-cyclohexanedimethanol (38/62 *cis*/*trans*), adipoyl chloride, sebacoyl chloride, terephthaloyl chloride, tin(ii) 2-ethyl-hexanoate, and anhydrous chloroform, DMF, THF, and toluene were obtained from Sigma Aldrich. Magnesium(iv) sulfate, 4-dimethylaminopyridine and pyridine were obtained from ACROS. 2,5-Furan-dicarboxylic acid was obtained from ABCR. 1,4-Cyclohexanediol (46/54 *cis*/*trans*) was obtained from Alfa Aesar. Ethylene glycol was obtained from TCI. Standard laboratory solvents were obtained from Biosolve. Deuterated solvents were obtained from Buchem BV (Netherlands). The purchased compounds were used directly without further purification, unless otherwise specified.

### Characterization methods

^1^H-NMR and ^13^C-NMR spectra were recorded with a Bruker Ultrashield 300 spectrometer (300 MHz magnetic field). NMR-samples were prepared by dissolving *ca.* 10 mg of sample in 0.5 mL deuterated solvent, including dimethyl sulfoxide (DMSO-*d*_6_), deuterated chloroform (CDCl_3_), and deuterated trifluoroacetic acid (*d*-TFA). All spectra were referenced against tetramethylsilane (TMS), or residual solvent peak from the deuterated solvent.

Molecular weight (*M*_n_, *M*_w_) and dispersity (*Ð*) of the polymers were calculated after gel permeation chromatography (GPC) on a PSS SECcurity GPC system using Agilent 1260 Infinity instrument technology. The GPC system was equipped with two PFG combination medium micro-columns with 7 μm particle size (4.6 × 250 mm, separation range 100–1.000.000 Da), a PFG combination medium pre-column with 7 μm particle size (4.6 × 30 mm), and a Refractive Index detector (RI). Distilled 1,1,1,3,3,3-hexafluoroisopropanol (HFIP) containing 0.019% sodium trifluoroacetate was used as mobile phase at 40 °C, with a 0.3 mL min^−1^ flow rate. The GPC apparatus was calibrated with poly(methyl methacrylate) standards obtained from PSS. GPC samples were prepared by dissolving 5 mg of polymer in 1.5 mL HFIP overnight under constant shaking, the samples were filtered over a 0.2 μm PTFE syringe filter prior to injection.

Thermal stability of compounds and screening of polymerization conditions were performed *via* thermogravimetric analysis (TGA) using a TA Instruments Q500. Experiments were performed under a nitrogen atmosphere with a heating rate of 10 °C min^−1^, from room temperature (RT) up to 700 °C. For polymerization screening experiments the sample was heated to the desired reaction temperature (generally 180, 200, or 220 °C) at a rate of 10 °C min^−1^ and kept isothermal for the desired reaction time. Thermal transition temperatures of the polymers were analyzed *via* differential scanning calorimetry (DSC) using a TA Instruments DSC Q2000. Typically, two heating and cooling runs were performed at 10 °C min^−1^, where the first heating was used to erase any thermal history in the samples. The glass-transition (*T*_g_), melt-transition (*T*_m_), crystallization (*T*_c_), and cold-crystallization (*T*_cc_) temperatures were obtained from the second heating and cooling run. DSC samples were prepared by loading 3–5 mg oven-dried samples in Tzero Hermetic Aluminium pans.

Polarized optical microscopy (POM) images were recorded on an Olympus BX53DP 26, equipped with a Linkam HFSX350 Hotstage. POM was used to determine melt-temperatures of samples which could not be measured *via* DSC (*e.g.* because of degradation upon melting). POM samples were loaded on a microscopy slide and heated at a rate of 10 °C min^−1^. The melt-temperature or range was determined by visual aid of the samples becoming isotropic in nature.

Matrix assisted laser desorption/ionization-time of flight-mass spectroscopy (MALDI-ToF-MS) analysis was recorded on a Voyager DESTR from Applied Biosystems (laser frequency 20 Hz, 337 nm and a voltage of 25 kV). The matrix material was DCTB (40 mg mL^−1^ in HFIP). Potassium trifluoroacetate was added as cationic ionization agent (5 mg mL^−1^ in HFIP). The polymer sample was dissolved in HFIP (1 mg mL^−1^), to which the matrix material and the ionization agent were added (5 : 1 : 5). If the samples were not fully dissolved, the mixture was filtered over a 0.2 μm PTFE syringe filter prior to placing on the target plate.

### Synthesis methods

#### Enantiomeric enrichment of 1,3-cyclopentanediol *via* fractional distillation

1,3-Cyclopentanediol from Sigma Aldrich was obtained as an *cis*/*trans* enantiomeric mixture having >95% purity. The commercial batch was analyzed prior to use *via* NMR and chiral GC to determine the *cis*/*trans* ratio, and was found to be 15/85 *cis*/*trans* (see ESI for analysis†). Based on the different boiling point of the *cis*/*trans* enantiomers, the commercial batch was purified by fractional distillation under vacuum. Various *cis*/*trans* ratios were obtained *via* batch-wise fractional distillation; Generally, 1,3-cyclopentanediol was loaded in a 50 mL 2-neck round-bottom flask, equipped with a 10 cm vigreux column, distillation 3-way adapter, condenser path, and a vacuum adapter to collect different fractions. Temperature of the oil-bath was varied from 130 °C to 160 °C. Temperature of the boiling phase was measured in the 3-way adapter, and the depth of the vacuum was noted. All fractions were obtained either as colorless liquids or as colorless solids: 7.6% *cis* (110 °C, 0.85 mbar), 14% *cis* (125 °C, 1.4 mbar), 20.7% *cis* (120 °C, 1.6 mbar), 30% *cis* (116 °C, 1.2 mbar), 41% *cis* (111 °C, 1.3 mbar).

#### Synthesis of FDCA-Cl

2,5-Furandicarboxylic acid (16.5 gr, 0.11 mol) was loaded in a 100 mL 3-neck round-bottom flask, equipped with a N_2_-inlet and a condenser, and was purged 3 times with argon/vacuum cycles. Thionyl chloride (27.5 mL, 0.38 mol) and DMF (100 μL, catalytic) were added, and the reaction mixture was refluxed for 4 hours at 85 °C, until the reaction mixture was a clear solution. A N_2_-flow over the reaction ensured the formed gasses to pass through a gas-washing bottle (250 mL 0.2 M aq. NaOH) attached *via* the top of the condenser. After cooling, the excess thionyl chloride was removed *in vacuo*, the product was isolated in several recrystallization steps from toluene as white crystals (15.2 gr, 75% yield).

#### General synthesis of trimer pre-polyesters

**Scheme 2:**
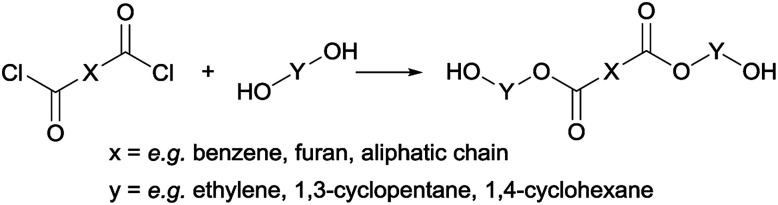
General synthesis route to obtain trimer pre-polyesters.

A generally applicable synthesis method for the preparation of trimer pre-polyesters has been developed (Scheme 2). The syntheses were performed in anhydrous conditions. A solution of 9 mmol diol, 7 mmol pyridine, and catalytic amount of DMAP in 5 mL anhydrous THF was stirred in an ice bath in a 25 mL 2-neck round-bottom flask equipped with a condenser. At 0 °C, under N_2_-flow, a solution of 3 mmol di-acid chloride in 3 mL anhydrous THF was added dropwise. After stirring the reaction mixture overnight, the THF was removed *in vacuo*. The product was extracted with CHCl_3_ (3 × 50 mL) and washed 10w% aq. Cu_2_SO_3_ (3 × 50 mL), and with 0.01 M aq. HCl (3 × 50 mL). The CHCl_3_ extract was dried with MgSO_4_, filtered, and reduced *in vacuo*. Generally, the extracts could be used as is, however some required further purification by precipitation from CHCl_3_ in ether. Typically the trimers from FDCA and terephthalic acid were obtained as white solids. Purity was confirmed by NMR, and/or LC-MS. The pre-polyesters were mostly isolated as trimers, however also some pentamers and heptamers are present. Detailed synthesis and characterization of the trimers used in this study are supplied in the ESI.†

### Polymerization screening methods

#### Small scale screening in TGA

Trimer pre-polyesters were polymerized in the TGA with or without catalyst. Trimers with catalyst were prepared by dissolving the trimer in CHCl_3_ followed by the addition of 1 mol% catalyst solution (0.01 M in anhydrous CHCl_3_). Next, the CHCl_3_ was removed *in vacuo*, and the trimers with catalyst were stored in a desiccator until use. Polymerization in the TGA was performed by loading 5–10 mg of sample, followed by heating at a rate of 10 °C min^−1^ to the desired polymerization temperature. Next, the samples were kept isothermal for the desired polymerization time, after which they were cooled to room temperature and used for analysis.

#### Small scale screening reactor design and validation

**Fig. 1:**
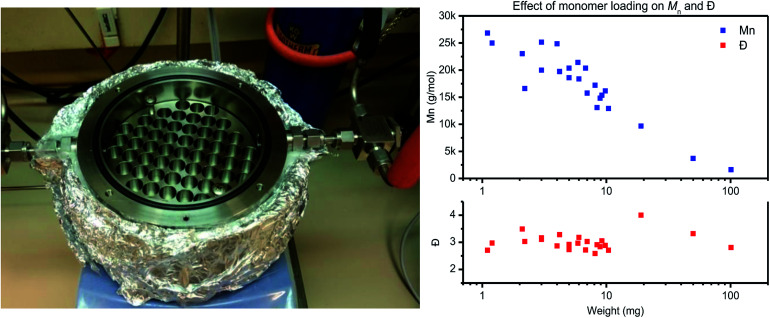
The left image depicts the used stainless-steel reactor with a block for 57 HPLC vials for polycondensation screening reactions. The image on the right depicts the effect of monomer loading (1–100 mg) on *M*_n_ (top) and *Ð* (bottom) for PET polymerized in the same reactor.

The small-scale polycondensation reactions were carried out in a stainless-steel reactor. The design for this reactor (Fig. 1, left) is based on earlier work of Gruter *et al.*:^11^ the stainless steel reactor block is designed to fit on top of a heating plate. To either side an inlet/outlet switch is attached for vacuum/N_2_ control. On the inside is an interchangeable reactor block with space for up to 57 HPLC vials. Reactor temperatures were monitored *via* one temperature sensor in the bottom–middle of the reactor and *via* a temperature sensor located in the side of the reactor wall. The reactor lid is closed with 4 screws, and a rubber O-ring providing air-tight sealing. Due to the large mass of the reactor block heating from 25 °C to 200 °C is achieved in approximately 30 minutes. Temperature calibration experiments were performed using a temperature probe in oil inside the reactor and consistently showed a temperature in between the set temperature, and the temperature measured on the side as is shown in the ESI.†

The reactor is designed for thin-film polymerization reactions, as thin-film polycondensation reactions do not require stirring for effective condensate removal. Therefore the film thickness should be low enough so that diffusion and mass transfer do not limit the rate of polymerization. For the materials used in this study, placement of 4 mg of polymer material in standard HPLC vial results in a thin-film with a thickness of roughly 200 μm (taking into account an inner vial radius of *r* = 2.5 mm and assuming a polymer density of *ρ* = 1.0 g mm^−3^ (*V* = (*π* × *r*^2^ × *h*)/*ρ*)).

To test the actual effect of monomer loading on the polymerization results, a PET trimer – 1,4-bis(ethyleneglycol-terephthalate) – was polymerized with a loading varying from 1 mg to 100 mg (Fig. 1, right). With increasing monomer loading from 1 mg to 100 mg, the *M*_n_ values drops from >25 kg mol^−1^ to <5 kg mol^−1^, respectively. For a monomer loading around 10 mg, *M*_n_ values around 15 kg mol^−1^ were consistently obtained on all runs. Therefore, to ensure repeatability of the polymerization experiments, in combination with ease in weighing, further work with this reactor is based on a monomer loading of 10 ± 1 mg. A detailed overview of the polymerization reactions performed for assessment of the repeatability of the thin-film polymerization reactions is provided in the ESI.†

#### General small-scale screening reactor polymerization

Trimer pre-polyesters were polymerized in a stainless-steel reactor block with or without catalyst. All the vials were weighed before and after reaction to monitor the weight loss during polymerization. Generally, 10 ± 1 mg of trimer was loaded in each vial, and 1 mol% of catalyst solution (0.01 M in anhydrous CHCl_3_) was added when required. The reactor block was placed in the reactor and next the reactor was closed after which it was purged 3× with a N_2_/vacuum cycle. The reactor was heated under a low N_2_-flow to the desired polymerization temperature, and kept at isothermal conditions for the desired polymerization time. Reduced pressure was applied during the polymerization when required. Polymerization was stopped by switching from vacuum to N_2_, followed by cooling of the reactor. After cooling down, the vials were weighed and the visible color and state of the polymer materials were noted.

## Results and discussion

### Trimer synthesis

Following previous work of Sweileh *et al.*,^25^ a general synthesis method for the preparation of trimer pre-polyesters, shown in Scheme 2, was developed. Although the use of acid chlorides is not sustainable, it allows for synthesis of trimer pre-polyesters, which do not suffer from evaporation and sublimation during thin-film polycondensation conditions. In other words, these trimer pre-polyesters are excellent candidates for rapid screening and optimization of polymerization conditions. Please note, for bulk polymerizations the use of acid chlorides is unnecessary as dimethyl esters can readily be used.

**Fig. 2:**
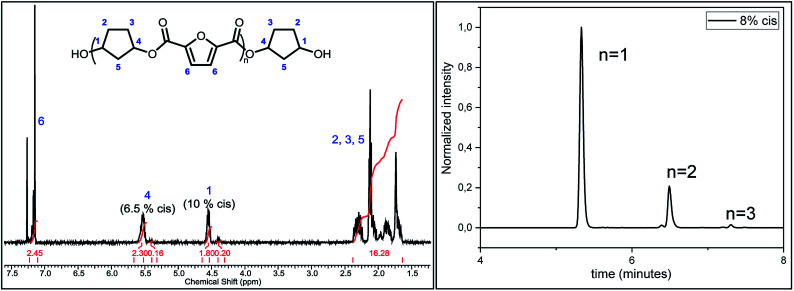
The image on the left depicts the ^1^H-NMR spectrum of the CP-F trimer with 8/92 *cis*/*trans* ratio. The image on the right displays the elution of the same trimer in LC-MS, highlighting the presence of small amounts of pentamer and heptamer.

To evaluate the effect of *cis*/*trans* ratio in CPdiol, 1,3-cyclopentanediol-furanoate (CP-F) trimers with varying *cis* content (8%, 20%, 30%, and 40%) were prepared. In spite of the addition of a 3-fold excess of free diol in the reaction mixture, some traces of pentamer and heptamer oligomers were found in addition to the expected trimer oligomers. The presence of trimers, observed from ^1^H-NMR, LC-MS, and GPC analysis, is exemplified in Fig. 2 for CP-F trimer having a 8/92 *cis*/*trans* ratio. A detailed overview of the analysis of the other trimers used in this work is provided in the ESI.† In general, with the used synthesis method, the ratio of trimer/pentamer/heptamer oligomers was 1/0.15/0.01. Nevertheless, all oligomers could be synthesized successfully using the reported synthesis method.

Interestingly, we observed that the CP-F trimers with the trimers with increasing *cis* content displayed increased discoloration after drying at 110 °C under vacuum. To investigate the cause of the increased discoloration the oven-dried samples were analyzed with LC-MS. In addition to the trimer/pentamer/heptamer distribution, new peaks with a mass difference of −18, and −84 g mol^−1^ were identified. The weight loss of 18 g mol^−1^ likely corresponds to the occurrence of a dehydration reaction, whereas the weight loss of 84 g mol^−1^ suggests the liberation of a 3-cyclopentenol (CPol) group.

To obtain the products in a pure form, the samples were dissolved in chloroform, resulting in a clear solution in combination with black insoluble particles, which were filtered out. Precipitation in heptane yielded the desired products, ranging from off-white to light brown in color. Interestingly, with increasing *cis* content, the yield dropped from ∼80% to ∼50%. Overall, these observations suggest that CPdiol exhibits limited thermal stability, where the *cis* isomer seems less thermally stable than the *trans* isomer. An image of the discolored products and the analysis of the trimers before and after purification are provided in the ESI.†

### Evaluation and optimization of polymerization conditions

**Fig. 3:**
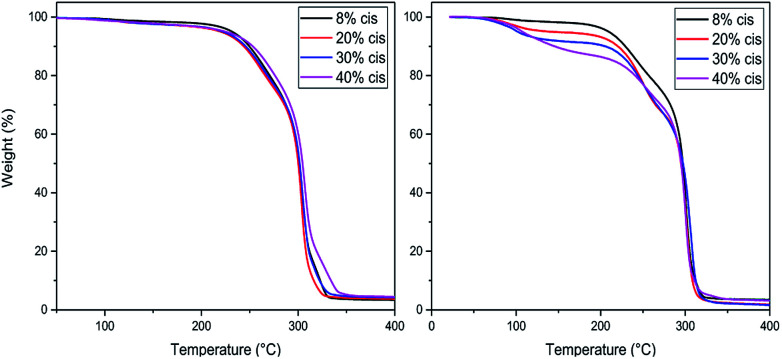
TGA experiments for CP-F trimer, with varying *cis* content, with a heating ramp of 10 °C min^−1^, non-catalyzed (left) and catalyzed (right).

TGA studies were performed to ascertain the right polymerization conditions for each trimer and preventing potential degradation resulting from the thermal instability of the CP-F trimers. From the left image in Fig. 3, it can be observed that the CP-F trimers are all thermally stable up to 200 °C, irrespective of the *cis*/*trans* ratio. Considering that no evaporation, sublimation, or degradation occurs below 200 °C, polymerization can be performed in TGA. In this case, all observed mass-loss should result from the generation of the condensate CPdiol: the mass loss at the theoretical maximum conversion equals the mass-loss of one CPdiol group of the trimer, which contributes to 31% of the weight.

To this end, all trimers were mixed with 1 mol% of Sn(ii)octanoate catalyst and the same TGA experiments were performed after extensive drying (Fig. 3, right). Indeed, upon the addition of the tin catalyst weight loss is found to start around 100 °C, followed by a stable plateau. Further heating results in degradation, starting above 220 °C. The amount of weight-loss between 100 °C and 200 °C increases with increasing *cis*-CPdiol content (respectively 3.9, 6.8, 9.1, and 13.5%, Fig. 3, right). These findings indicate that trimers having a higher *cis*-CPdiol content generate volatiles more rapidly during polymerization, potentially indicating an increased rate of polycondensation.

To identify the optimal polymerization temperature, all trimers were polymerized in the TGA for one hour at 180, 200, and 220 °C (Table 1). As observed previously, an increased weight-loss is observed with increasing *cis*-fraction, combined with an increased discoloration of the final product. Due to the absence of a vacuum-step in the polymerization in the TGA, the molecular weight of the resulting oligomers remained low, with *M*_n_ varying between 500 and 3000 g mol^−1^. The melting behavior of the polymers was determined *via* polarized optical microscopy (POM) equipped with a Linkam hot-stage, rather than in DSC due to the limited thermal stability of the samples. The hot-stage was heated at 10 °C min^−1^, and the visual off-set of melting was noted.

**Table 1:** Results of small-scale polymerizations performed in TGA

Polymer	*Cis*	Pol. *T*.	W. loss	Color	*M*_n_ (g mol^−1^)	*M*_w_ (g mol^−1^)	*Đ*	*T*_m_ (°C)	*T*_d_ (°C)
Poly(CP-F)	8%	180 °C	11.1%	Light yellow	1224	2448	2.0	260	285
Poly(CP-F)	8%	200 °C	19.1%	Light orange	1846	3628	1.97	300	308
Poly(CP-F)	8%	220 °C	26.1%	Light brown	2769	6401	2.31	285	295
Poly(CP-F)	20%	180 °C	9.2%	Light orange	546	1206	2.21	290	296
Poly(CP-F)	20%	200 °C	20.3%	Light orange	1602	3484	2.17	285	296
Poly(CP-F)	20%	220 °C	26.8%	Light brown	2680	6772	2.53	267	281
Poly(CP-F)	30%	180 °C	11.8%	Light brown	525	1161	2.21	245–280	280
Poly(CP-F)	30%	200 °C	23.3%	Light brown	1660	3724	2.24	245–282	280
Poly(C-PF)	30%	220 °C	28.9%	Brown	2342	7169	3.06	254–274	274
Poly(CP-F)	40%	180 °C	14.2%	Transp. brown	663	1414	2.13	—	n.d.
Poly(CP-F)	40%	200 °C	23.5%	Transp. brown	2071	4503	2.18	—	n.d.
Poly(CP-F)	40%	220 °C	29.1%	Dark brown	2330	8129	3.49	—	n.d.

As expected, all samples showed rapid signs of (further) discoloration with the evolution of gasses directly after melting, indicating that the degradation temperature (*T*_d_) is very close to the melting temperature (*T*_m_). Additionally, increasing of the *cis*-fraction resulted in an increase in *M*_w_ and a decrease in *T*_m_, until a fully amorphous material is obtained for the polymer having 40% *cis*-CPdiol. These findings are in coherence with literature on the *cis*/*trans* effect of other cycloaliphatic diols.

**Fig. 4:**
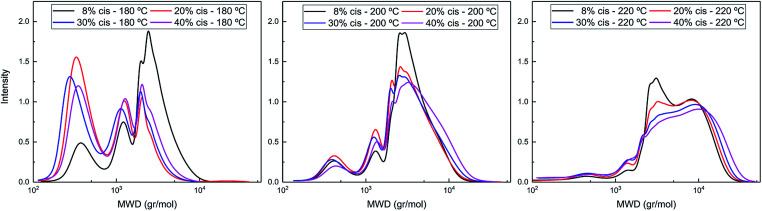
GPC traces of poly(CP-F) polymerized at 180 °C (left), 200 °C (middle), and 220 °C (right), with varying *cis*-1,3-CPdiol content from 8% (black), 20% (red), 30% (blue) to 40% (purple).

The difference in polymerization behavior of varying *cis*-CPdiol content becomes apparent by analyzing the GPC traces (Fig. 4). In general, the trimer containing 8% *cis*-CPdiol polymerized rapidly at 180 °C, indicated by the narrow *Ð* and the relatively high molecular weight compared to the polymer with higher *cis*-CPdiol content. When increasing the reaction temperature to 200 °C, the rise of a high molecular weight tail is observed in the molecular weight distribution. In fact, this is true for all samples, and is particularly dominant in the sample having 40% *cis*-CPdiol. Further heating to 220 °C broadens the molecular weight distribution even further, and this effect again becomes more apparent with increasing amount of *cis*-CPdiol. It should be noted that the oligomers with increasing *cis*-CPdiol content became increasingly difficulty to dissolve in the HFIP solvent. In fact, especially for the oligomers containing 30 and 40% *cis*-CPdiol, black insoluble components remained in the HFIP solvent, which were filtered off prior to GPC analysis. This indicates that the GPC traces shown in Fig. 4 might not reflect the true polymer content as not all material has dissolved.

Nevertheless, the formation of insoluble polymer particles suggests the presence of a cross-linking side reaction: the presence of a bimodal distribution is highly unlikely in polycondensation reactions considering the continuously occurring transesterification reaction in combination with Flory's equireactivity theory. Instead, considering the increasing discoloration and decreasing solubility of polymers with increasing *cis*-CPdiol content, we consider it more likely that *cis*-CPdiol undergoes a degradative cross-linking reaction in addition to the polycondensation reaction, thereby generating high molecular weight components. To assess whether such a side reaction proceeds during polymerization, the obtained oligomers have been analyzed using MALDI-TOF-MS.

### Oligomer analysis using MALDI-ToF-MS

**Fig. 5:**
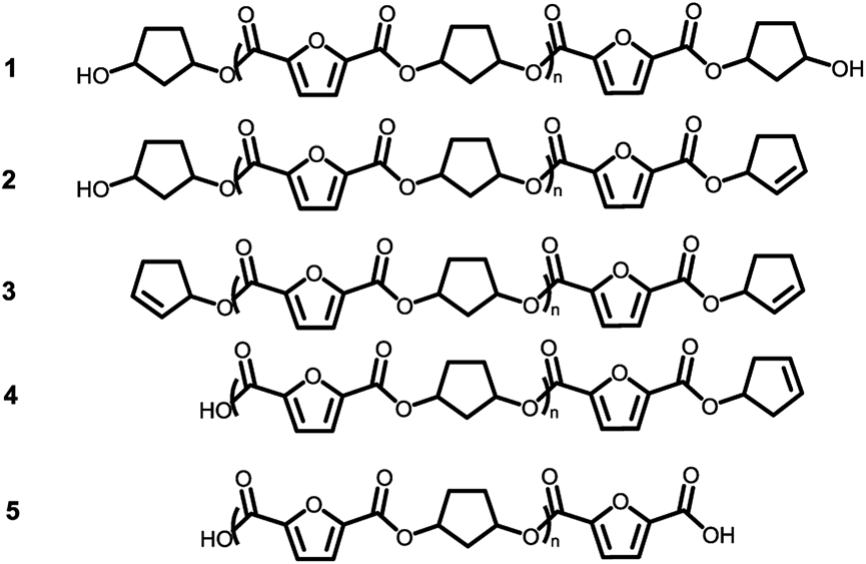
Expected products for poly(CP-F) before and after thermal stability experiments.

**Fig. 6:**
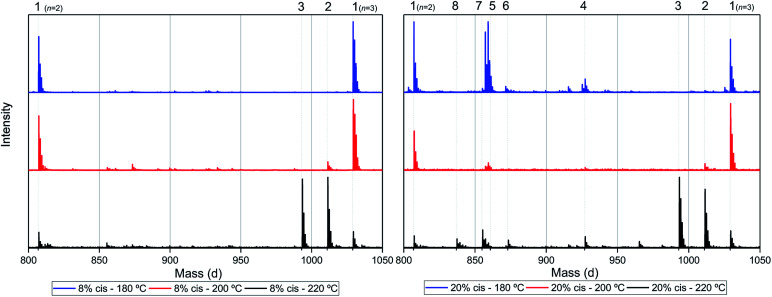
MALDI-ToF-MS spectra of poly(CP-F) polymerized at 180 °C (blue, top), 200 °C (red, middle), and 220 °C (black, bottom), with *cis* fractions of 8% (left), and 20% (right). Zoomed in on 750–1050 dalton, between repeating units *n* = 2 (*m*/*z* = 806.82) and *n* = 3 (*m*/*z* = 1029.02) of linear diol 1.

**Fig. 7:**
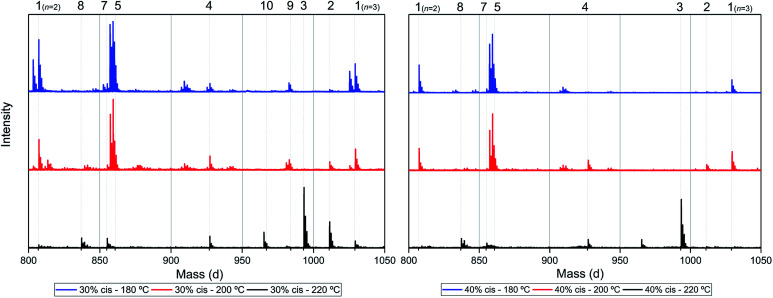
MALDI-ToF-MS spectra of poly(CP-F) polymerized at 180 °C (blue, top), 200 °C (red, middle), and 220 °C (black, bottom), with *cis* fractions of 30% (left), and 40% (right). Zoomed in on 750–1050 dalton, between repeating units *n* = 2 (*m*/*z* = 806.82) and *n* = 3 (*m*/*z* = 1029.02) of linear diol 1.

Assuming that the CP-F polycondensation reaction proceeds without side-reactions, only the presence of linear chains with cyclopentanol (CPol) groups on both chain ends is expected (structure 1, Fig. 5). As is observed from LC-MS analysis, thermal dehydration (2, 3), and loss of 1,3-CPol end-group (4, 5) can be expected to occur as a side-reaction during polymerization. The mass distributions of the oligomers after polymerization at 180 °C (blue, top), 200 °C (red, middle), and 220 °C (black, bottom) of 1,3-CP-F trimers having 8% (Fig. 6, left), 20% (Fig. 6, right), 30% (Fig. 7, left), and 40% *cis*-CPdiol (Fig. 7, right) are reported. The depicted region is selected between 800 and 1050 dalton, in order to show the distribution between repeat units *n* = 2 (*m*/*z* = 806.82) and *n* = 3 (*m*/*z* = 1029.02) of expected main product 1. A more detailed list, the calculated masses of possible chain distributions, and the full MALDI-TOF-MS spectra of the trimers are provided in the ESI section.†

As can be seen from Fig. 6, left, the polymer having 8% *cis*-CPdiol polymerized at 180 °C shows a single distribution of the expected diol product 1 (as is noted on top of the figure). Dehydration of the alcohol end-group takes place when increasing the polymerization temperature, leading to a rise of products 2 and 3 at the expense of product 1, as is clearly visible from the MALDI-ToF-MS spectrum taken at 220 °C. Similar to the samples having 8% *cis*-CPdiol, an increase in polymerization temperature for the sample having 20% *cis*-CPdiol results in dehydration of the CPdiol end-groups, as is indicated by the rise in reaction products 2 and 3.

Interestingly, the presence of product 1 becomes less abundant at 180 °C as the *cis* content increases. Instead, the samples with 20%, 30%, and 40% *cis*-CPdiol polymerized at 180 °C contain products corresponding to chains with one (structure 4) or two (structure 5) carboxylic acid end-groups. Since the carboxylic acid moiety is not present as starting material, they have to be generated during polymerization. Generally, carboxylic acid groups can be generated through ester hydrolysis by water, (degradative) chain-scission of ester bonds, or complete removal of a 3-cyclopentenol end-group.^26,27^ Hydrolysis by water would lead to a distribution of chains with a CPol end-group on one side, and a carboxylic acid on the other side. However, such a distribution was not detected in MALDI-TOF-MS analysis (data provided in ESI†). Instead, (degradative) chain-scission of ester bonds would lead to product 4 and 5, however would also inherently lead to a drop in molecular weight, which was not observed in GPC analysis (Fig. 4). Thus it seems likely that only the CPol end-groups have been removed from the product, leading to the observed drop in product 1, and rise in the carboxylic acid terminated products 4 and 5.

An increase in polymerization temperature from 180 °C to 200 °C and 220 °C for CP-F samples having 20%, 30%, and 40% *cis*-CPdiol results in a decrease in distributions 4 and 5. This could potentially be resulting from thermal decarboxylation of the generated FDCA moieties,^26,27^ which yields a mono-substituted furan based diene, which can undergo a Diels–Alder reaction with CPene end-groups, also providing a potential route towards branched or cross-linked species. We will elaborate further on this hypothesis later in this work.

**Scheme 3:**
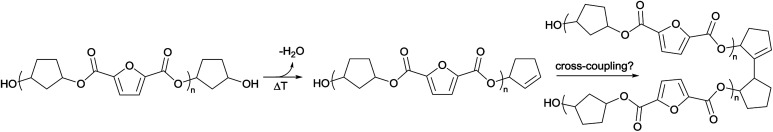
Anticipated degradation process of oligomers. A cyclopentanol end-group can undergo thermal dehydration, generating a cyclopentene end-group, which in turn can undergo a cross-coupling reaction.

**Fig. 8:**
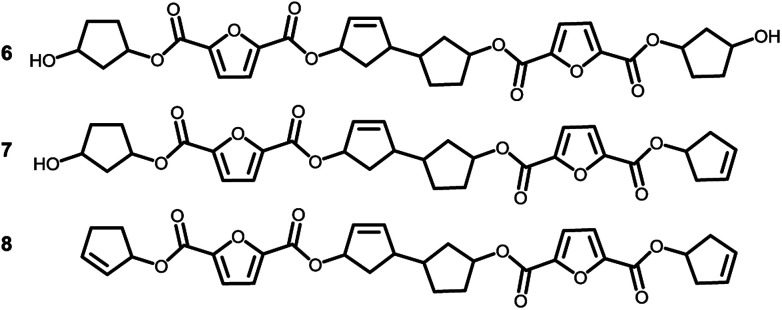
Expected products for poly(CP-F) after CPene cross-coupling reaction and thermal dehydration.

To recall, both LC-MS (provided in the ESI†) and the MALDI-TOF-MS analysis depicted in Fig. 6 suggest that thermal dehydration takes place on the external OH group of CPdiol, thereby generating cyclopentene end-groups. These cyclopentene groups are likely to undergo cross-coupling reactions (Scheme 3), leading to products 6, 7, and 8 (Fig. 8), thereby providing a means to build up high molecular weight materials, in addition to the polycondensation reaction. Indeed, a rise in products 6, 7, and 8 can be observed in the samples with increasing *cis*-CPdiol content, confirming that CPene cross-coupling reactions are occurring. This CPene–CPene coupling reaction also explain the rise in molecular weight observed in GPC analysis of samples having higher *cis*-CPdiol content (Fig. 4).

**Scheme 4:**
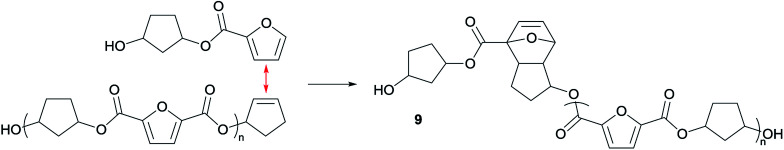
Anticipated Diels–Alder side reaction between a furan group and a CPene group leading to a polymer distribution 9.

As indicated earlier, an additional chain-extension reaction that can take place is the Diels–Alder addition reaction between decarboxylated furan moieties and CPene groups. This Diels–Alder side reaction would result into the formation of distribution 9, as shown in Scheme 4. Indeed, such a distribution is found in the polymer having 30% *cis*-CPdiol, (highlighted by dotted line 9 in Fig. 7, left). With increased polymerization temperature to 220 °C, distribution 9 decreases, and a distribution with a mass loss of 18 g mol^−1^ arises (dotted line 10, Fig. 7). It is likely that distribution 10 corresponds to the dehydrated product of 9. In fact, this distribution is observed in both polymers having 20% and 40% *cis*-CPdiol, polymerized at 220 °C. These findings provide further proof for the occurrence of a Diels–Alder reaction between the degradation products.

It should be noted that the obtained mass-distributions in these MALDI-ToF-MS analysis seem limited to fit only linear chains that are ending with either carboxylic acid, CPol, or CPene end-groups. Although cross-linked or branched mass-distributions are expected given the presence of insoluble components in GPC analysis, such cross-linked or branched fractions are not detected in these MALDI-ToF-MS experiments. Most likely these branched or cross-linked materials are filtered off during sample preparation or are too low in concentration to measure. Nevertheless, the current MALDI-ToF-MS data provides valuable insights in the polymerizability and thermal stability of CPdiol as monomer in polycondensation reactions.

### Stability difference *cis* and *trans* 1,3-cyclopentanediol

**Fig. 9:**
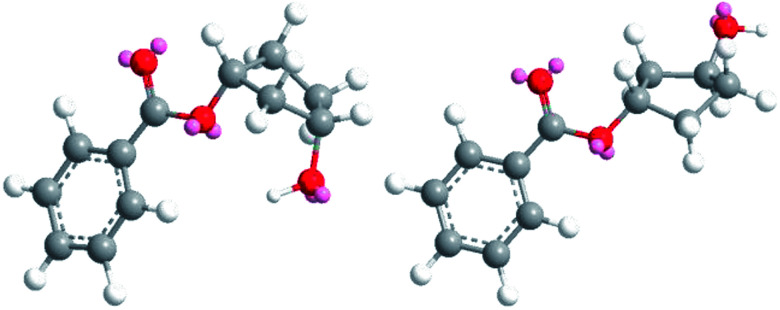
Minimized energy calculations of the mono-benzoate ester of *cis*-CPdiol (left) and *trans*-CPdiol (right). Calculated with Chemdraw 3D v17, *via* MM2 molecular dynamics and minimize energy.

The obtained results in the polymerization stability study suggest that there is an inherent difference in thermal stability between the *cis* and *trans* isomer of 1,3-cyclopentanediol: structures with mostly *trans*-CPdiol end-groups undergo thermal dehydration at temperatures of 200 °C and higher, thereby generating CPene end-groups. Structures with *cis*-CPdiol end-groups rather undergo degradative ester bond cleavage at 180 °C instead, thereby generating free carboxylic acid groups in addition to CPene end-groups. It is possible that the origin of this stability difference can be found in the different conformations of these two isomers. The conformations of *cis*- and *trans*-1,3-cyclopentanediol have been evaluated using theoretical and NMR investigations in the work of Koniotou *et al.*^21^ These authors reported that the most stable conformers for *cis*-CPdiol have the hydroxyl groups in axial positions, allowing for intramolecular hydrogen bonding.^28^ In contrast, the most stable conformers for *trans*-CPdiol have the hydroxyl groups in equatorial positions, where intramolecular hydrogen bonding is not observed. When these findings are extrapolated to esters of 1,3-cyclopentanediol, preliminary minimized energy calculations in ChemDraw 3D software show similar axial and equatorial positioning (Fig. 9). These conformations possibly allow *cis*-CPdiol to undergo intramolecular hydrogen bonding of the external hydroxyl group with the ester bond on the same CP-ring (Fig. 9, left).

**Scheme 5:**
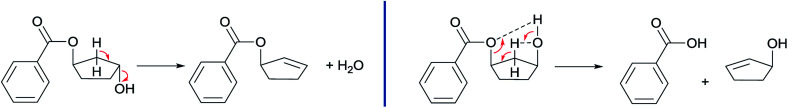
Proposed thermal degradation mechanisms of *trans* (left) and *cis* (right) 1,3-CPdiol.

Combining our experimental findings with the preferred conformations of CPdiol esters, suggests that the origin in thermal stability between *cis*- and *trans*-CPdiol might be related to the absence or presence of intramolecular hydrogen bonding. Even though in-depth mechanistic studies of the degradation are beyond the scope of this research, a possible degradation mechanism based on the conformations of CPdiol is postulated in Scheme 5. In both cases, β-hydrogen elimination takes place, followed by the breaking of a σ-bond: either C–OH (left), or C–OR (right). Without intramolecular hydrogen bonding it appears that the weakest σ-bond to break is C–OH, leading to dehydration in the *trans* isomers (Scheme 5, left). However, with intramolecular hydrogen bonding it appears that C–OR becomes the weaker σ-bond, leading to removal of the CPol group, generation an acid-terminated chain (Scheme 5, right).

Furthermore, dehydration *via* β-hydrogen elimination is acid catalyzed, and indeed it is observed that with increasing acid-terminated species 5, dehydrated species (2 and 3) start to form at the expense of diol terminated chains (1) (Fig. 6 and 7). Therefore, it appears that the thermal degradation mechanism of *cis*-1,3-CPdiol leads to free acid species, which in turn catalyzes the dehydration of the remaining *trans* 1,3-CPdiol end-groups, leading to an even lower thermal stability of the polymer.

Please note that there is no increase in the low molecular weight fractions observed at the evaluated polymerization temperatures (Fig. 4), indicating that the difference in thermal stability at these temperatures seems to originate solely from the difference in *cis* or *trans* end-groups. The thermal stability seems enhanced once the 1,3-CPdiol end-groups are completely polymerized even though ‘normal’ β-hydrogen chain scission still occurs at elevated temperatures.^27^ Unfortunately, we were unable to study the rate difference in β-hydrogen chain scission between the *cis* and *trans* conformers of CPdiol. This is related to the inability of the poly(CP-F) to build up high molecular weight under the explored polymerization conditions.

### Assessment of 1,3-cyclopentanediol as building block

In the previous section we reported on the thermal lability of the *cis*-CPdiol, and the corresponding thermal limitations. Even though presence of 15% *cis* content in the commercial batch of 1,3-cyclopentanediol limits the polymerization temperature to 180 °C, polyesters could be prepared in small-scale thin-film polycondensation reactions. Apart from poly(CP-F), trimers of CPdiol with adipic acid (A), sebacic acid (S), and terephthalic acid (T) have been synthesized. Furthermore, trimers with structurally related 1,4-cyclohexanediol (CH) (50/50 *cis*/*trans* ratio) and 1,4-cyclohexanedimethanol (CHdm) (30/70 *cis*/*trans* ratio) have been prepared and polymerized. The polymerizations were performed at 180 °C with 1 mol% of catalyst for 3 hours under vacuum. The resulting polyesters were analyzed for their molecular weight and thermal properties, as is summarized in Table 2. Note, the full details of all polymerization experiments are available in the ESI.† The polymers are abbreviated according to the diol and diacid used, *e.g.* poly(1,3-cyclopentane-terephthalate) is abbreviated as poly(CP-T).

**Table 2:** Overview of properties of prepared polymers of cycloaliphatic diols 1,3-cyclopentanediol (CP), 1,4-cyclohexanediol (CH), and 1,4-cyclohexanedimethanol (CHdm), bridged by terephthalate (T), adipate (A), and sebacate (S) moieties

	*M*_n_ (g mol^−1^)	*M*_w_ (g mol^−1^)	*T*_g_ (°C)	*T*_m_ (°C)
CP-T	5k	11k	65 °C	240–300^a^ °C
CP-A	17k	57k	−30 °C	Amorphous
CP-S	25k	92k	−38 °C	45 °C
CH-T	1.1k	1.4k	n.o.	300+^b^ °C
CH-A	13k	37k	5 °C	117 °C
CH-S	16k	45k	−22 °C	75 °C
CHdm-T	4k	7k	n.o.	240+^b^ °C
CHdm-A	12k	27k	−30 °C	92 °C
CHdm-S	18k	42k	−38 °C	44 °C

a Degradation upon melt.

b Obtained *via* polarized optical microscopy.

**Fig. 10:**
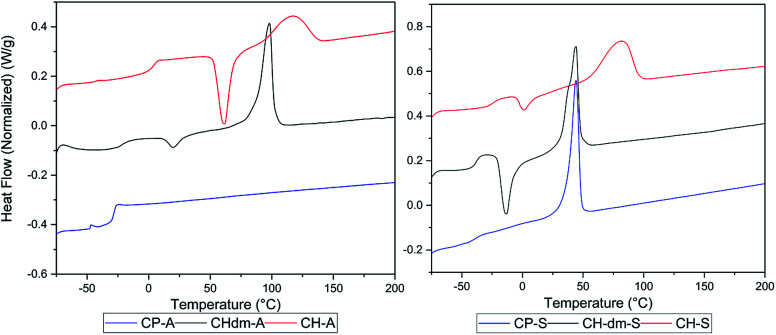
DSC curves of prepared polymers of cycloaliphatic diols 1,3-cyclopentanediol (CP, bottom), 1,4-cyclohexanedimethanol (CHdm, middle), and 1,4-cyclohexanediol (CH, top) with soft blocks adipate (A, left) and sebacate (S, right). Heating and cooling performed with 10 °C min^−1^, 2^nd^ heating run is shown.

Poly(CP-T) was obtained as semi-crystalline polymer with an unstable melt above 240 °C, which is likely a result from the previously observed instability of CPdiol based materials. Furthermore, the melting temperature of poly(CP-S) is significantly lower compared to its 1,4-cyclohexanediol based counterpart, *i.e.* 45 °C *versus* 75 °C. Instead, the melting behavior of poly(CP-S) is comparable to the polymer containing its 1,4-cyclohexanedimethanol counterpart, as they have similar melting temperatures. Furthermore, poly(CP-A) is fully amorphous, whereas poly(CH-A) and poly(CHdm-A) are both semi-crystalline in nature (Fig. 10, left) unfortunately, the *T*_g_ of poly(CH-T) and poly (CHdm-T) could not be observed in DSC analysis, likely due to their high crystallinity. With respect to the glass transition temperature (*T*_g_), polymers containing CPdiol are rather comparable to materials containing CHdmol: both monomers yield polymers with significantly lowered *T*_g_ compared to polymers containing CHdiol. These results indicate that polyesters with CPdiol have a reduced rigidity compared to polyesters with CHdiol, and should be compared to the more flexible CHdmol instead. However, although the thermal transitions are comparable, the rate of crystallization of polymers containing CPdiol is significantly faster than for polymers containing CHdmol, as is indicated by the absence of cold-crystallization for poly(CP-S), shown in Fig. 10, right.

Overall, this preliminary polymerization screening data shows that CPdiol can readily be polymerized under thin-film conditions with various dicarboxylic acids, despite the previously observed degradation reactions. We expect that the application of reduced pressure promotes the polycondensation reaction, and thereby limits the molecular weight build-up by the formed degradation products reported earlier. In fact, under these conditions, *M*_n_ values > 15 kg mol^−1^ are readily obtained for polymers based on CPdiol and sebacic acid or adipic acid. However, the use of high-melting polymers with CPdiol should be avoided due to the inherently unstable melt of CPdiol, preventing melt processing.

## Conclusions

A generic synthesis route has been developed to prepare trimer pre-polyesters from various diols, in particular for 1,3-CPdiol, and di-acid-chloride monomers. These trimers were directly used in small-scale thin-film polycondensation reactions as they do not have the drawback of monomer evaporation. Using this approach, TGA was used as a screening technique to identify the thermal stability, and to determine optimal polymerization conditions of the CPdiol based trimers. In general we observed that esters having the *trans*-CPdiol isomer are found to be stable up to 180 °C, and undergo dehydration at 200 °C and higher. In contrast, esters having the *cis*-1,3-CPdiol isomer are thermally labile, as they undergo rapid degradation already at 180 °C, which is attributed to the ability to form intramolecular hydrogen bonding. Using the optimal polymerization temperature of 180 °C, high molecular weight polymers (>15 kg mol^−1^) having 1,3-CPdiol and sebacic acid or adipic acid were successfully made. A comparative study showed that polyesters having CPdiol exhibit lower rigidity and crystallinity than their CHdiol counterpart. Instead, the CPdiol moiety is found to be comparable CHdmol, which can likely be attributed to the less stable half-boat conformation of CPdiol compared to the stable boat-conformation of cyclohexane structures.

## Conflicts of interest

There are no conflicts to declare.

## Supplementary Material

RA-008-C8RA08811J-s001

## References

[cit1] Sara M. , Brar S. K. and Blais J. F., in Platform Chemical Biorefinery, Elsevier, 2016, pp. 249–283

[cit2] Nakajima H., Dijkstra P., Loos K. (2017). Polymers.

[cit3] Hillmyer M. A. (2017). Science.

[cit4] Zhu Y., Romain C., Williams C. K. (2016). Nature.

[cit5] Bomtempo J.-V., Chaves Alves F., de Almeida Oroski F. (2017). Faraday Discuss..

[cit6] Hoogenboom R., Schubert U. S. (2005). Rev. Sci. Instrum..

[cit7] Meier M. A. R., Hoogenboom R., Schubert U. S. (2004). Macromol. Rapid Commun..

[cit8] Murphy V., Bei X., Boussie T. R., Brümmer O., Diamond G. M., Goh C., Hall K. A., Lapointe A. M., Leclerc M., Longmire J. M., Shoemaker J. A. W., Turner H., Weinberg W. H. (2002). Chem. Rec..

[cit9] Potyrailo R. A., Lemmon J. P., Leib T. K. (2003). Anal. Chem..

[cit10] Brocchini S., James K., Tangpasuthadol V., Kohn J. (1998). J. Biomed. Mater. Res..

[cit11] Gruter G.-J. M., Sipos L., Adrianus Dam M. (2012). Comb. Chem. High Throughput Screening.

[cit12] Li G., Li N., Zheng M., Li S., Wang A.-Q., Cong Y., Wang X., Zhang T. (2016). Green Chem..

[cit13] Wang J., Liu X., Jia Z., Sun L., Zhang Y., Zhu J. (2018). Polymer.

[cit14] Celli A., Marchese P., Sisti L., Dumand D., Sullalti S., Totaro G. (2013). Polym. Int..

[cit15] Kibler C. J., Bell A., Smith J. G. (1964). J. Polym. Sci., Part A: Gen. Pap..

[cit16] Colonna M., Berti C., Binassi E., Celli A., Fiorini M., Marchese P., Messori M., Brunelle D. J. (2011). Polym. Int..

[cit17] Berti C., Celli A., Marchese P., Marianucci E., Sullalti S., Barbiroli G. (2010). Macromol. Chem. Phys..

[cit18] Zhang M., Moore R. B., Long T. E. (2012). J. Polym. Sci., Part A-1: Polym. Chem..

[cit19] Kelsey D. R., Scardino B. M., Grebowicz J. S., Chuah H. H. (2000). Macromolecules.

[cit20] Sandhya T. E., Ramesh C., Sivaram S. (2007). Macromolecules.

[cit21] Abraham R. J., Koniotou R. (2003). Magn. Reson. Chem..

[cit22] Mattson A., Orrenius C., Öhrner N., Unelius C. R., Hult K., Norin T., Homsi M. N., Kuske F. K. H., Haugg M., Trabesinger-Rüf N., Weinhold E. G. (1996). Acta Chem. Scand..

[cit23] Fransson A.-B. L., Xu Y., Leijondahl K., Bäckvall J.-E. (2006). J. Org. Chem..

[cit24] Agou T., Ohata R., Mizuhata Y., Tokitoh N., Fukumoto H., Kubota T. (2018). J. Fluorine Chem..

[cit25] Sweileh B. A., Al-Qalawi H. R., Mohammad H. A. (2014). J. Appl. Polym. Sci..

[cit26] Tsanaktsis V., Vouvoudi E., Papageorgiou G. Z., Papageorgiou D. G., Chrissafis K., Bikiaris D. N. (2015). J. Anal. Appl. Pyrolysis.

[cit27] Terzopoulou Z., Tsanaktsis V., Nerantzaki M., Achilias D. S., Vaimakis T., Papageorgiou G. Z., Bikiaris D. N. (2016). J. Anal. Appl. Pyrolysis.

[cit28] Saegebarth K. (1960). J. Org. Chem..

