# Supplementary material for: Small-scale screening of novel biobased monomers: the curious case of 1,3-cyclopentanediol
Source: RSC Adv. 2018 Nov 29;8(70):39818–28. doi: 10.1039/c8ra08811j (PMC9091289; doi:10.1039/c8ra08811j)
Supplement: RA-008-C8RA08811J-s001 [file RA-008-C8RA08811J-s001.pdf]

Electronic Supplementary Material (ESI) for RSC Advances.
This journal is © The Royal Society of Chemistry 2018

Small-scale screening of novel biobased monomers: the curious case of 1,3-cyclopentanediol

Authors:

G.J. Noordzij[a,b], C.H.J.T. Dietz[c], N. Leoné[b], C.H.R.M. Wilsens*[b], S. Rastogi[b]

[a.] Chemelot InSciTe, Urmonderbaan 20F, NL-6167 RD Geleen, The Netherlands.
[b]Aachen-Maastricht Institute of Biobased Materials (AMIBM), Faculty of Science and Engineering, Maastricht University, Brightlands Chemelot Campus, 6167 RD Geleen, The Netherlands.
[c]Inorganic Membranes and Membrane Reactors, Dept. Chemical Engineering and Chemistry, Eindhoven University of Technology, PO Box 513, Eindhoven, The Netherlands
* Corresponding author

Contents

Small-scale screening of novel biobased monomers: the curious case of 1,3-cyclopentanediol 1

Reactor block design 3

GC data 4

Detailed synthesis of trimers 5

LC-MS data 8

NMR data trimers 9

NMR analysis of trimer 1,2-ethyleneglycol-terephthalate 9

NMR analysis of trimer 1,3-cyclopentanediol-terephthalate 10

NMR analysis of trimer 1,3-cyclopentanediol-furanoate – 8% *cis* 12

NMR analysis of trimer 1,3-cyclopentanediol-furanoate – 20% *cis* 12

NMR analysis of trimer 1,3-cyclopentanediol-furanoate – 30% *cis* 13

NMR analysis of trimer 1,3-cyclopentanediol-furanoate – 40% *cis* 14

NMR analysis of trimer 1,3-cyclopentanediol-adipate 15

NMR analysis of trimer 1,3-cyclopentanediol-sebacate 16

NMR analysis of trimer 1,4-cyclohexanediol-terephthalate 17

NMR analysis of trimer 1,4-cyclohexanediol-adipate 19

NMR analysis of trimer 1,4-cyclohexanediol-sebacate 20

NMR analysis of trimer 1,4-cyclohexanedimethanol-terephthalate 21

NMR analysis of trimer 1,4-cyclohexanedimethanol-adipate 23

NMR analysis of trimer 1,4-cyclohexanedimethanol-sebacate 24

Detailed polymerization of small-scale screening 25

MALDI-ToF-MS 30

MALDI-ToF-MS expected linear chains 30

MALDI-ToF-MS - side reactions with cyclopentene group 31

MALDI-ToF-MS - other possible side reactions 32

Obtained MALDI-ToF-MS spectra 8% cis 33

Obtained MALDI-ToF-MS spectra 20% cis 34

Obtained MALDI-ToF-MS spectra 30% cis 35

Obtained MALDI-ToF-MS spectra 40% cis 36

Reactor block design

The temperature consistency in the reactor was tested by measuring the temperature on 3 different points in the reactor: temperature sensor in the bottom part, in the side part, and inside in oil in a HPLC vial. There was consistently a small deviation in temperature inside the reactor as compared to the set & bottom temperature, which is caused by heat loss of the reactor itself to the surrounding environment. This temperature-loss was compensated for in the settings: *i.e.* for a desired polycondensation temperature of 220 ºC, the temperature was set to 228 ºC.

Table 1. Obtained values for temperature control throughout the stainless steel reactor.

**Set temp (ºC)**	**Control bottom**	**Control side**	**Oil in vials**
100 ºC	98 ºC	92 ºC	92 ºC
150 ºC	148 ºC	140 ºC	142 ºC
200 ºC	198 ºC	186 ºC	188 ºC
250 ºC	246 ºC	230 ºC	237 ºC

The reactor was validated for polycondensation experiments by the polymerization of a known polymer: poly(ethylene terephthalate). First a consistency check was performed by polymerizing batches of 10 mg over 3 runs (Table 3), and secondly the effect of loading on the polycondensation was checked (Table 4). Overall, the polycondensation is considered to perform consistently well in order to perform polycondensation reactions on the novel polyesters described in this work.

GC data

Chiral GC data for *cis*/*trans* ratio determination of 1,3-cyclopentanediol.

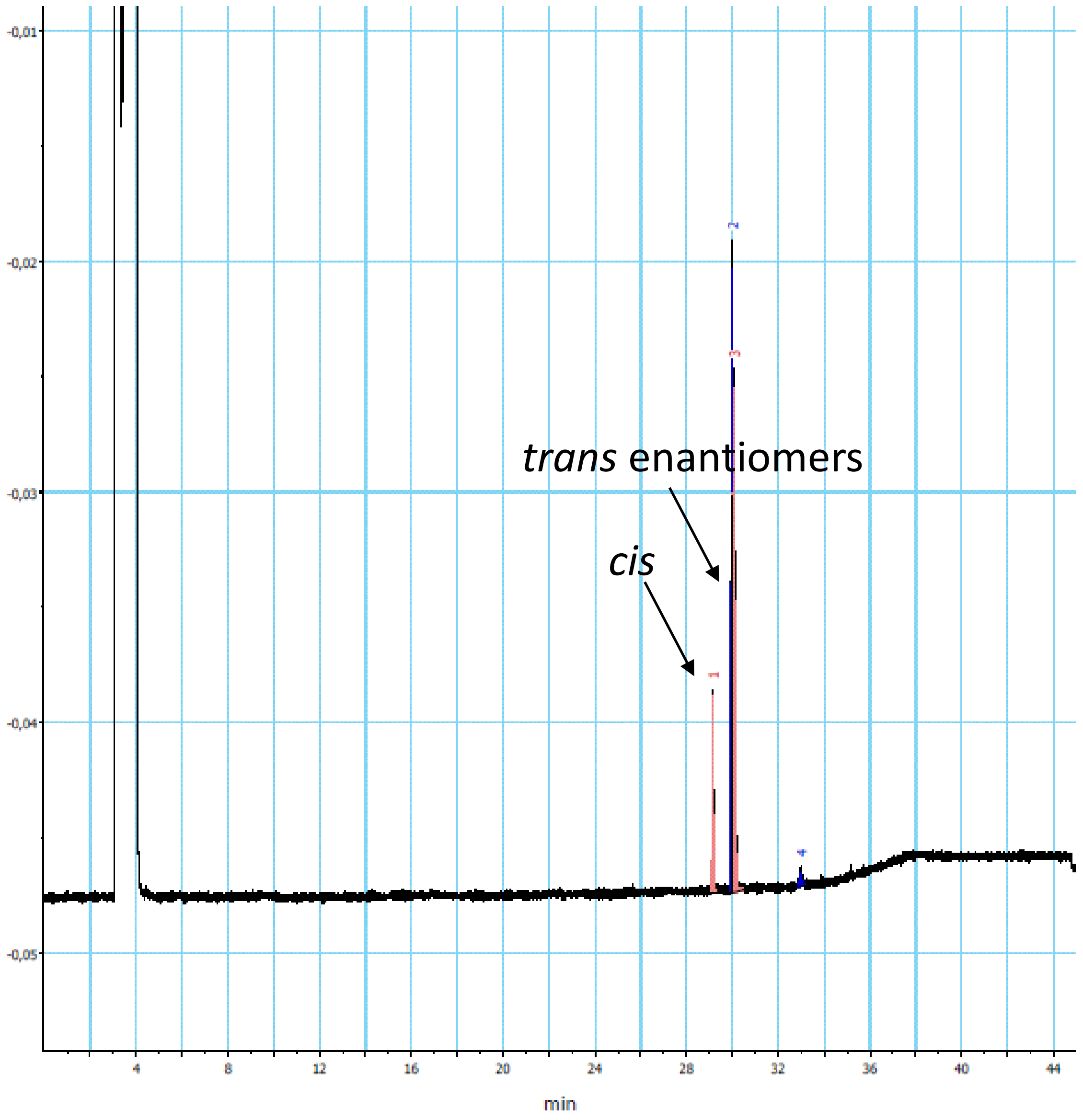

Figure 1. Chiral GC chromatogram on the commercial batch of 1,3-cyclopentanediol.

Peak	Fill	Peak Name	t_R	t_S	t_E	H (V)	H_{Norm}	A (V.s)	A_{Norm}	$W_{0,1}$	Asym	Effic	Res
1			29,14	29,06	29,33	0,01	14,55	0,04	12,12	0,12	1,75	1137189	
2			29,97	29,90	30,02	0,03	46,61	0,11	37,81				
3			30,05	30,02	30,48	0,02	37,45	0,14	47,77				
4			32,97	32,78	33,18	0,00	1,39	0,01	2,30	0,26	1,31	292616	
						0,06	100,00	0,29	100,00				

Figure 2. Chiral GC data on the commercial batch of 1,3-cyclopentanediol, with 12.12% *cis*, and 85.58% *trans*.

Detailed synthesis of trimers

A generally applicable synthesis method for the preparation of trimer pre-polyesters has been developed based on previously reported synthesis method. The syntheses were performed in anhydrous conditions. A molar ratio of 3:1 diol:di-acid was used. A typical reaction is described: A solution of 9 mmol diol, 7 mmol pyridine, and catalytic amount of DMAP in 5 mL anhydrous THF was stirred in an ice bath in a 25 mL 2-neck round-bottom flask equipped with a condenser. At 0 °C, under N_2-flow, a solution of 3 mmol di-acid chloride in 3 mL anhydrous THF was added dropwise. After stirring the reaction mixture overnight, the THF was removed in vacuo. The product was extracted with $CHCl_3$ (3x 50 mL), and washed with 10w% aq. Cu_2SO_3 (3x 50 mL), and with 0.01 M aq. HCl (3x 50 mL). The $CHCl_3$ extract was dried with $MgSO_4$, filtered, and reduced in vacuo. Typically the extracts could be used as is, however some could be further purified by precipitation from $CHCl_3$ in ether. Typically the trimers from FDCA and terephthalic acid were obtained as white solids. Purity was confirmed by NMR, and/or LC-MS. The pre-polyesters were mostly isolated as trimers, however also some pentamers and heptamers are present.

Trimer 1,2-ethyleneglycol-terephthalate

The product was obtained as white solids (0.9 gr, 72% yield). ^{1}H NMR ($CDCl_3$, 300 MHz): δ 8.11 (bs, 4H), 4.95 (m, 0.93H), 4.69 (m, 2.11H), 4.31 (m, 1.80H), 3.70 (m, 1.81H). ^{13}C NMR ($CDCl_3$, 300 MHz): δ 165.5 (*C*=O), 134.4 & 133.79 (O=C-*C*), 130.1 & 130.0 (*C*=*C*), 67.5 (HO-CH_2-*C*H_2-O-C), 63.7 (C-O-*C*H_2-*C*H_2-O-C), 59.4 (HO-*C*H_2-CH_2-O-C).

Trimer 1,3-cyclopentanediol-terephthalate

The product was obtained as white solids (5.05 gr, 76% yield). ^{1}H NMR ($CDCl_3$, 300 MHz): δ 8.04 (s, 4H), 5.52 (bs, 0.28H), 5.39 (bs, 1.59H), 5.23 (bs, 0.23 H), 4.67 (bs, 1.77 H), 2.8 (m, 2.49H), 1.95 (m, 5.43H), 1.72 (m, 2.23H), 1.55 (m, 1.73H). ^{13}C NMR ($CDCl_3$, 300 MHz): δ 165.2 (*C*=O), 77.2 (*C*H-OR ring) 70.8 (*C*H-OH ring), 42.4 (*C*H_2 ring), 33.6 (*C*H_2 ring), 30.5 (*C*H_2 ring).

Trimer 1,3-cyclopentanediol-furanoate

The product was obtained as white solids (1.99 gr, 62% yield). ^{1}H NMR ($CDCl_3$, 300 MHz): δ 7.16 (m, 2H), 5.53 (m, 1.90H), 5.39 (m, 0.16 H), 4.55 (m, 1.30H), 4.40 (m, 0.16H), 2.32 (m, 2.59H), 2.13 (m, 6.32H), 1.87 (m, 2.27H), 1.70 (m, 1.36H). ^{13}C NMR ($CDCl_3$, 300 MHz): δ 157.7 (*C*=O), 146.7 (*C*-O, furan ring) 118.2 (*C*-*C*, furan ring) 77.4 (*C*H-OR ring) 72.1 (*C*H-OH ring), 42.2 (*C*H_2 ring), 33.5 (*C*H_2 ring), 30.3 (*C*H_2 ring).

Trimer 1,3-cyclopentanediol-furanoate, -8% *cis*

The product was obtained as white solids (1.12 gr, 67% yield). After drying at 110 ºC discoloration occurred, the product was isolated by dissolving the mix in $CHCl_3$, filter out the brown solids, and reduce the $CHCl_3$ *in vacuo*, to re-obtain the product as white solids (0.64 gr, 38% yield). ^{1}H NMR ($CDCl_3$, 300 MHz): δ 7.12 (m, 2H), 5.46 (m, 2.27H), 4.60 (m, 1.74H), 4.51 (m, 0.16H), 2.22 (m, 2.53H), 2.12 (m, 5.91H), 1.85 (m, 2.56H), 1.73 (m, 1.82H). ^{13}C NMR ($CDCl_3$, 300 MHz): δ 158.4 (*C*=O), 146.8 (*C*-O, furan ring) 118.7 (*C*-*C*, furan ring) 79.7 (*C*H-OR ring) 73.2 (*C*H-OH ring), 41.6 (*C*H_2 ring), 32.8 (*C*H_2 ring), 30.3 (*C*H_2 ring).

Trimer 1,3-cyclopentanediol-furanoate, -20% *cis*

The product was obtained as off-white solids (1.34 gr, 80% yield). After drying at 110 ºC discoloration occurred, the product was isolated by dissolving the mix in $CHCl_3$, filter out the brown solids, and reduce the $CHCl_3$ *in vacuo*, to re-obtain the product as off-white solids (0.88 gr, 52% yield). ^{1}H NMR ($CDCl_3$, 300 MHz): δ

7.12 (m, 2H), 5.46 (m, 2.27H), 4.60 (m, 1.74H), 4.51 (m, 0.16H), 2.22 (m, 2.53H), 2.12 (m, 5.91H), 1.85 (m, 2.56H), 1.73 (m, 1.82H). ^{13}C NMR ($CDCl_3$, 300 MHz): δ 158.4 (*C*=O), 146.8 (*C*-O, furan ring) 118.7 (*C*-*C*, furan ring) 79.7 (*C*H-OR ring) 73.2 (*C*H-OH ring), 41.6 (*C*H_2 ring), 32.8 (*C*H_2 ring), 30.3 (*C*H_2 ring).

Trimer 1,3-cyclopentanediol-furanoate, -30% *cis*

The product was obtained as light brown solids (1.37 gr, 82% yield). After drying at 110 ºC discoloration occurred, the product was isolated by dissolving the mix in $CHCl_3$, filter out the brown solids, and reduce the $CHCl_3$ *in vacuo*, to re-obtain the product as light brown solids (0.91 gr, 54% yield).

^{1}H NMR ($CDCl_3$, 300 MHz): δ 7.12 (m, 2H), 5.46 (m, 2.27H), 4.60 (m, 1.74H), 4.51 (m, 0.16H), 2.22 (m, 2.53H), 2.12 (m, 5.91H), 1.85 (m, 2.56H), 1.73 (m, 1.82H). ^{13}C NMR ($CDCl_3$, 300 MHz): δ 158.4 (*C*=O), 146.8 (*C*-O, furan ring) 118.7 (*C*-*C*, furan ring) 79.7 (*C*H-OR ring) 73.2 (*C*H-OH ring), 41.6 (*C*H_2 ring), 32.8 (*C*H_2 ring), 30.3 (*C*H_2 ring).

Trimer 1,3-cyclopentanediol-furanoate, -40% *cis*

The product was obtained as yellow oil (0.98 gr, 87% yield). After drying at 110 ºC discoloration occurred, the product was isolated by dissolving the mix in $CHCl_3$, filter out the brown solids, and reduce the $CHCl_3$ *in vacuo*, to re-obtain the product as light brown solids (0.48 gr, 43% yield). ^{1}H NMR ($CDCl_3$, 300 MHz): δ 7.12 (m, 2H), 5.46 (m, 2.27H), 4.60 (m, 1.74H), 4.51 (m, 0.16H), 2.22 (m, 2.53H), 2.12 (m, 5.91H), 1.85 (m, 2.56H), 1.73 (m, 1.82H). ^{13}C NMR ($CDCl_3$, 300 MHz): δ 158.4 (*C*=O), 146.8 (*C*-O, furan ring) 118.7 (*C*-*C*, furan ring) 79.7 (*C*H-OR ring) 73.2 (*C*H-OH ring), 41.6 (*C*H_2 ring), 32.8 (*C*H_2 ring), 30.3 (*C*H_2 ring).

Trimer 1,3-cyclopentanediol-adipate

The product was obtained as orange oil (5.31 gr, 86% yield). ^{1}H NMR ($CDCl_3$, 300 MHz): δ 5.27 (m, 2.20H), 5.17 (m, 0.32H), 4.47 (m, 1.75H), 4.33 (m, 0.25H), 2.27 (m, 7.73H), 1.97 (m, 10.88H), 1.63 (m, 9.52H). ^{13}C NMR ($CDCl_3$, 300 MHz): δ 173.1 (*C*=O), 75.5 (*C*H-OR ring) 72.3 (*C*H-OH ring), 42.4 & 33.6 & 30.5 (*C*H_2 ring), 34.1 & 24.4 (*C*H_2 adipate).

Trimer 1,3-cyclopentanediol-sebacate

The product was obtained light orange oil (6.42 gr, 88% yield). ^{1}H NMR ($CDCl_3$, 300 MHz): δ 5.28 (m, 2.44H), 5.16 (m, 0.31H), 4.47 (m, 1.78H), 4.32 (m, 0.22H), 2.24 (m, 10.89H), 1.96 (m, 8.12H), 1.59 (m, 10.97H), 1.29 (bs, 11.89H). ^{13}C NMR ($CDCl_3$, 300 MHz): δ 173.6 (*C*=O), 75.4 (*C*H-OR ring) 72.3 (*C*H-OH ring), 42.4 & 33.6 & 30.4 (*C*H_2 ring), 34.5 & 29.0 & 24.9 (*C*H_2 sebacate).

Trimer 1,4-cyclohexanediol-terephthalate

The product was obtained as white solids (0.89 gr, 50% yield). ^{1}H NMR ($CDCl_3$, 300 MHz): δ 8.09 (m, 4H), 5.17 (bs, 1.24 H), 5.04 (bs .076 H), 3.83 (m, 1.56 H), 2.05 (m, 6.09 H), 1.71 (m, 10.28H). ^{13}C NMR ($CDCl_3$, 300 MHz): δ 165.2 (*C*=O), 129.5 (*C*=*C* terephthalic ring), 72.7 (*cis*-*C*H-OR), 70.8 (*trans*-*C*H-OR), 68.7 & 67.8 (*C*H-OH), 32.0 & 30.5 & 28.3 & 27.5 (*C*H_2 cyclohexane).

Trimer 1,4-cyclohexanediol-adipate

The product was obtained as a yellow oil (1.78 gr, 95% yield). ^{1}H NMR ($CDCl_3$, 300 MHz): δ 4.74 (ds, 2.92H), 3.74 (ds, 2.0H), 2.31 (s, 6.0H), 1.65 (m, 28.8H). ^{13}C NMR ($CDCl_3$, 300 MHz): δ 172.9 (*C*=O), 71.6 & 69.7 (*C*H-OR), 68.8 & 67.8 (*C*H-OH), 34.1 & 24.3 (*C*H_2 adipate), 32.1 & 30.5 & 28.6 & 27.4 (*C*H_2 cyclohexane).

Trimer 1,4-cyclohexanediol-sebacate

The product was obtained as white waxy solids (1.64 gr, 98% yield). ^{1}H NMR ($CDCl_3$, 300 MHz): δ 4.87 (ds, 2.5H), 3.77 (ds, 2.0H), 2.29 (m, 7.75H), 1.62 (m, 34.3H). ^{13}C NMR ($CDCl_3$, 300 MHz): δ 173.4 (*C*=O), 71.4 & 69.4 (*C*H-OR), 68.9 & 67.9 (*C*H-OH), 34.7 & 25.0 (*C*H_2 adipate), 32.2 & 30.5 & 29.0 & 27.4 (*C*H_2 cyclohexane).

Trimer 1,4-cyclohexanedimethanol-terephthalate

The product was obtained as white solids (1.36 gr, 66% yield). ^{1}H NMR ($CDCl_3$, 300 MHz): δ 8.10 (bs, 6.06H), 4.29 (ds, 2.07H), 4.19 (ds, 3.70H), 3.56 (ds, 1.35 H), 3.50 (ds, 2.64H), 1.90 (m, 21.6H), 1.11 (m, 6.65H). ^{13}C NMR ($CDCl_3$, 300 MHz): δ 165.9 (*C*=O), 134.2 (O=C-*C*, terephthalic ring),129.5 (*C*=*C* terephthalic ring), 70.3 & 68.0 (*C*H_2-OR), 68.4 & 66.1 (*C*H_2-OH), 40.4 + 37.4 (*C*H-OH), 37.4 & 34.6 (*C*H-OR), 29.1 & 28.7 & 25.6 & 25.1 (*C*H_2 cyclohexane ring).

Trimer 1,4-cyclohexanedimethanol-adipate

The product was obtained light yellow oil (1.94 gr, 89% yield). ^{1}H NMR ($CDCl_3$, 300 MHz): δ 3.93 (ds, 1.22H), 3.84 (ds, 2.49H), 3.48 (ds, 0.65H), 3.40 (ds, 1.35H), 2.26 (bs, 3.75H), 1.60 (m, 15.85H), 0.93 (m, 3.93H). ^{13}C NMR ($CDCl_3$, 300 MHz): δ 173.4 (*C*=O), 69.3 & 67.1 (*C*H_2-OR), 68.4 & 66.0 (*C*H_2-OH), 40.3 + 37.3 (*C*H-OH), 37.3 & 34.5 (*C*H-OR), 33.9 & 24.4 (*C*H_2, adipate), 29.0 & 28.8 & 28.7 & 25.5 & 25.3 & 25.1 (*C*H_2 cyclohexane ring).

Trimer 1,4-cyclohexanedimethanol-sebacate

The product was obtained colorless oil (1.74 gr, 91% yield). ^{1}H NMR ($CDCl_3$, 300 MHz): δ 3.98 (ds, 1.86H), 3.90 (ds, 3.96H), 2.90 (2.53H), 2.29 (m, 6.78H), 1.82 (bs, 7.95H), 1.43 (m, 31.10H), 1.00 (m, 6.81H). ^{13}C NMR ($CDCl_3$, 300 MHz): δ 174.0 (*C*=O), 69.3 & 67.0 (*C*H_2-OR), 68.4 & 66.1 (*C*H_2-OH), 40.4 + 37.3 (*C*H-OH), 37.3 & 34.3 (*C*H-OR), 34.3 & 25.1 (*C*H_2, sebacate), 29.0 & 28.7 & 25.5 (*C*H_2 cyclohexane ring).

LC-MS data

The trimers of 1,3-CP-F with various *cis* content were dried at 110 ºC, after which significant discoloration was visible (Figure 3). The obtained trimers where analyzed via LC-MS (Figure 4) to assess the degradation products (Table 2) prior to a second purification step.

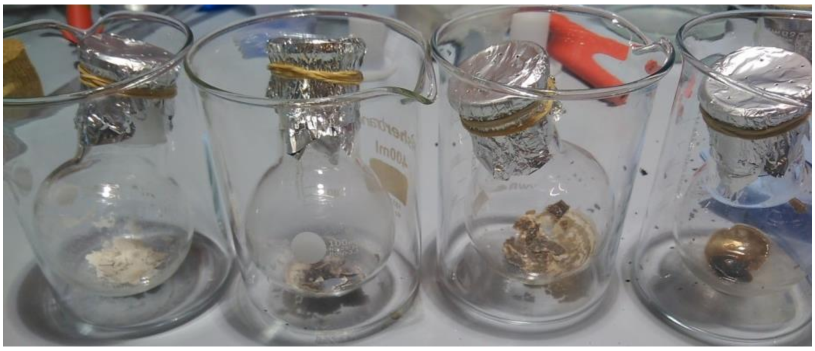

Figure 3. Obtained trimers of 1,3-CP-F, with varying *cis* content (8%, 20%, 30%, 40%, left to right), after drying at 110 ºC, showing various stages of discoloration.

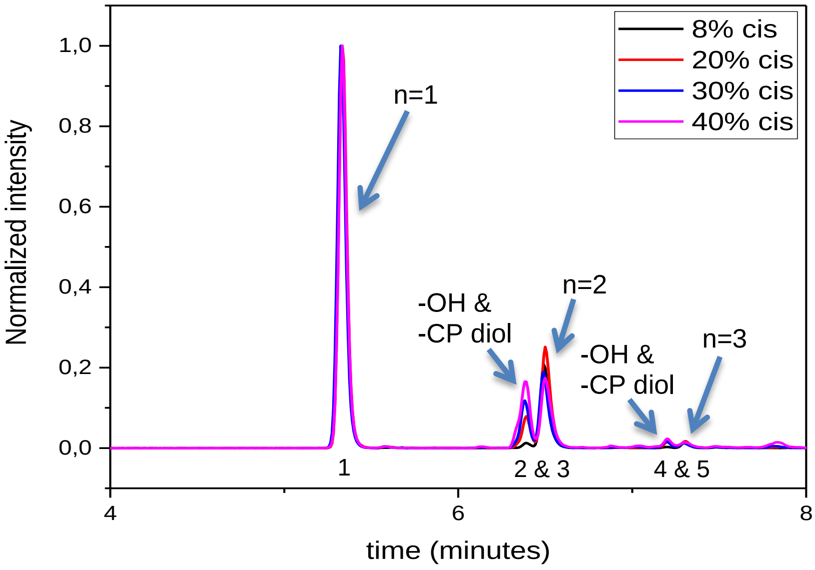

Figure 4. LC-MS data on partially degraded CP-F trimers after drying at 110 ºC.

Table 2. List of masses found for partially degraded 1,3-CP-F trimers in LC-MS, supporting Figure 4.

	n=1	n=2	n=3
Calculated mass →	324.3	546.5<br>528.5 (-OH)<br>462.4 (-CPol)	768.7<br>750.7 (-OH)<br>684.6 (-CPol)
Peak 1	342 (H_3O^+ adduct)		
Peak 2		463, 529, 547	
Peak 3		547, 564 (H_3O^+ adduct)	
Peak 4			685, 751, 769
Peak 5			769, 786 (H_3O^+ adduct)

NMR data trimers

NMR analysis of trimer 1,2-ethyleneglycol-terephthalate

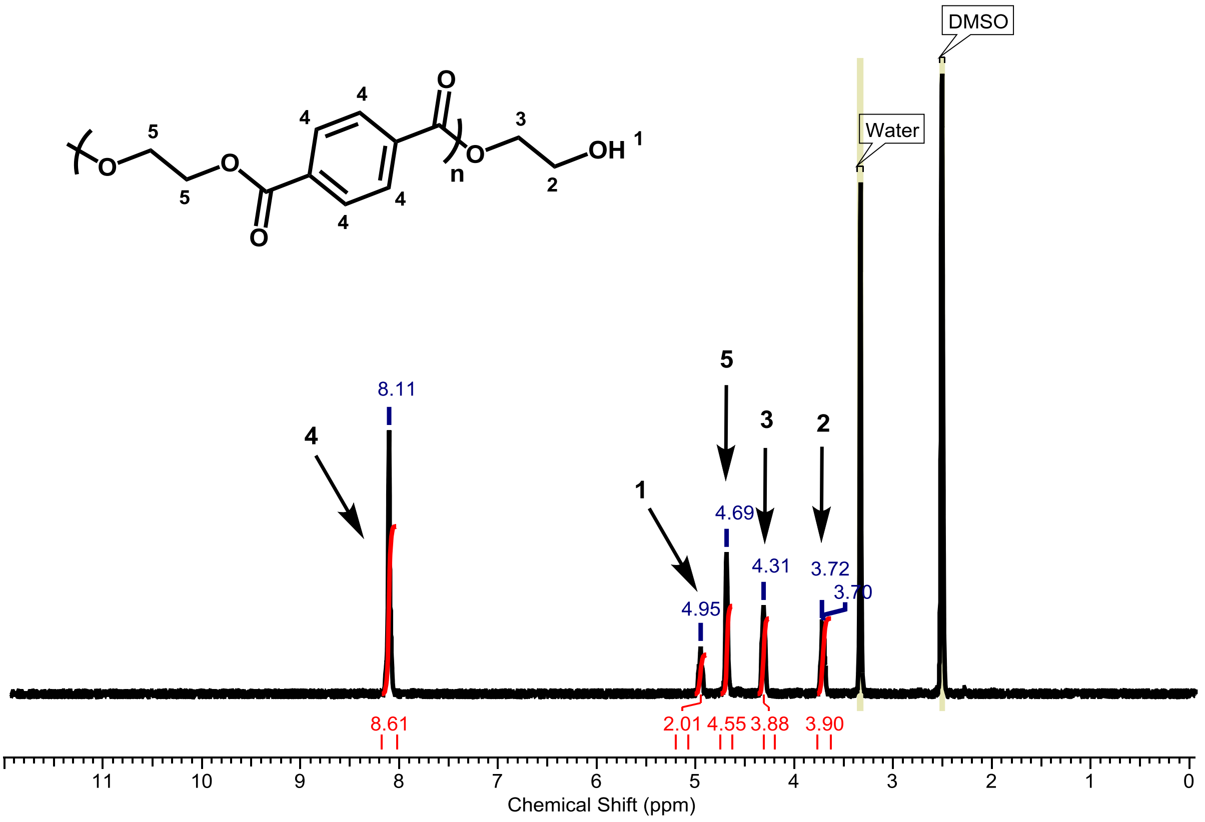

Figure 5. ^{1}**H-NMR analysis of trimer 1,2-ethyleneglycol-terephthalate.**

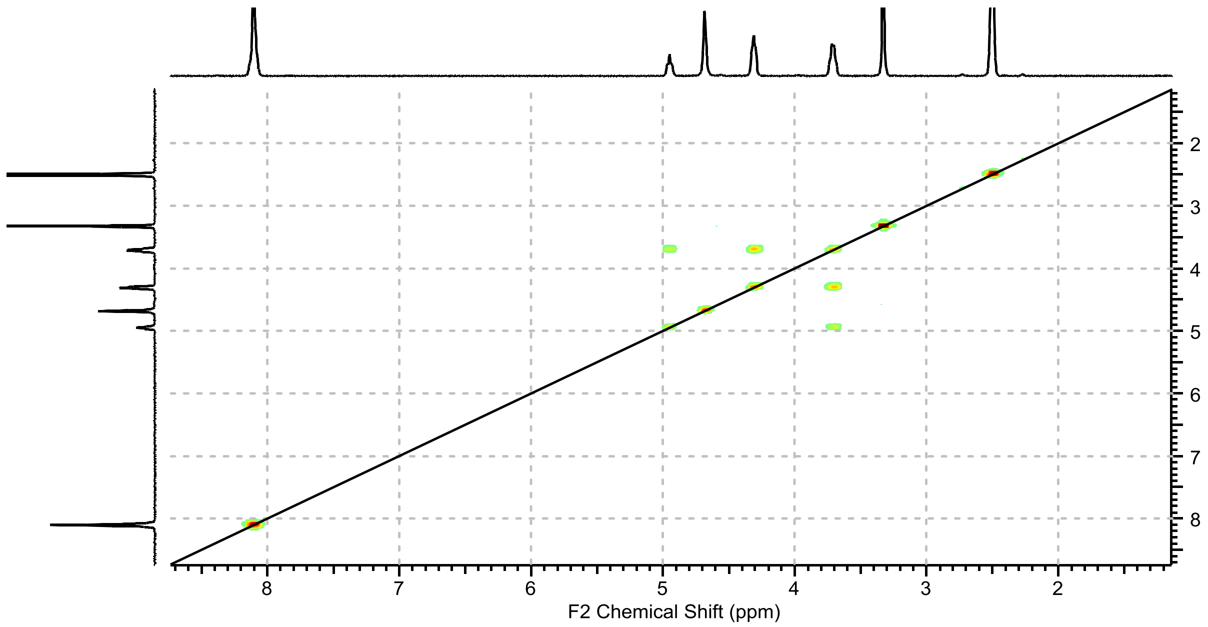

Figure 6. COSY (^{1}**H-**^{1}**H) NMR analysis of trimer 1,2-ethyleneglycol-terephthalate.**

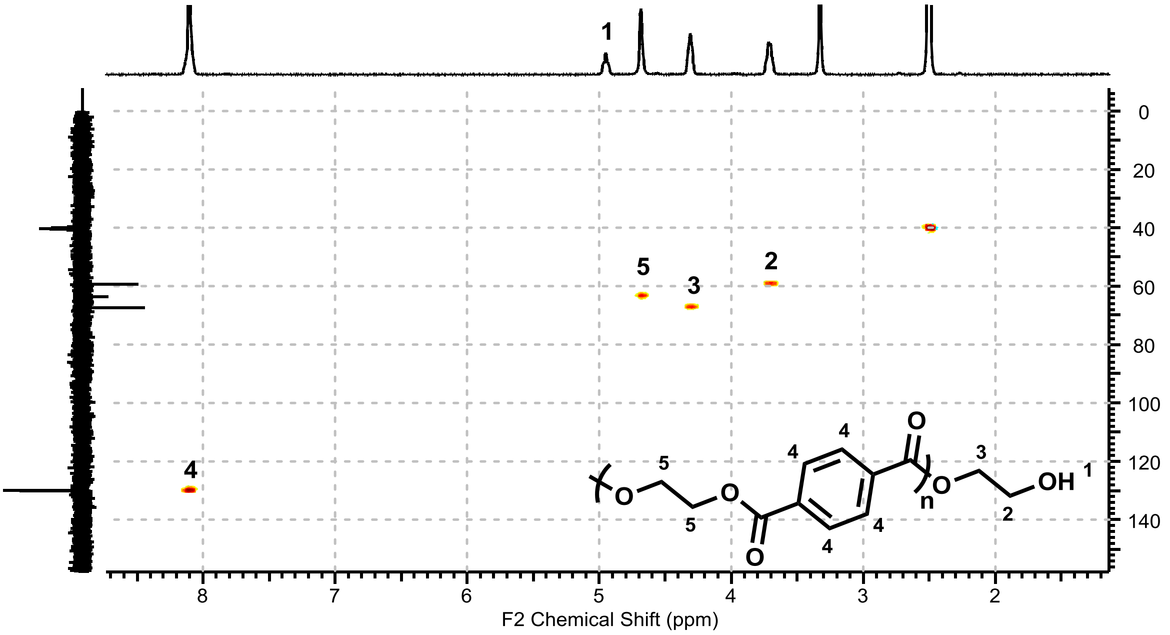

Figure 7. HSQC (¹H-DEPT) NMR analysis of trimer 1,2-ethyleneglycol-terephthalate.

NMR analysis of trimer 1,3-cyclopentanediol-terephthalate

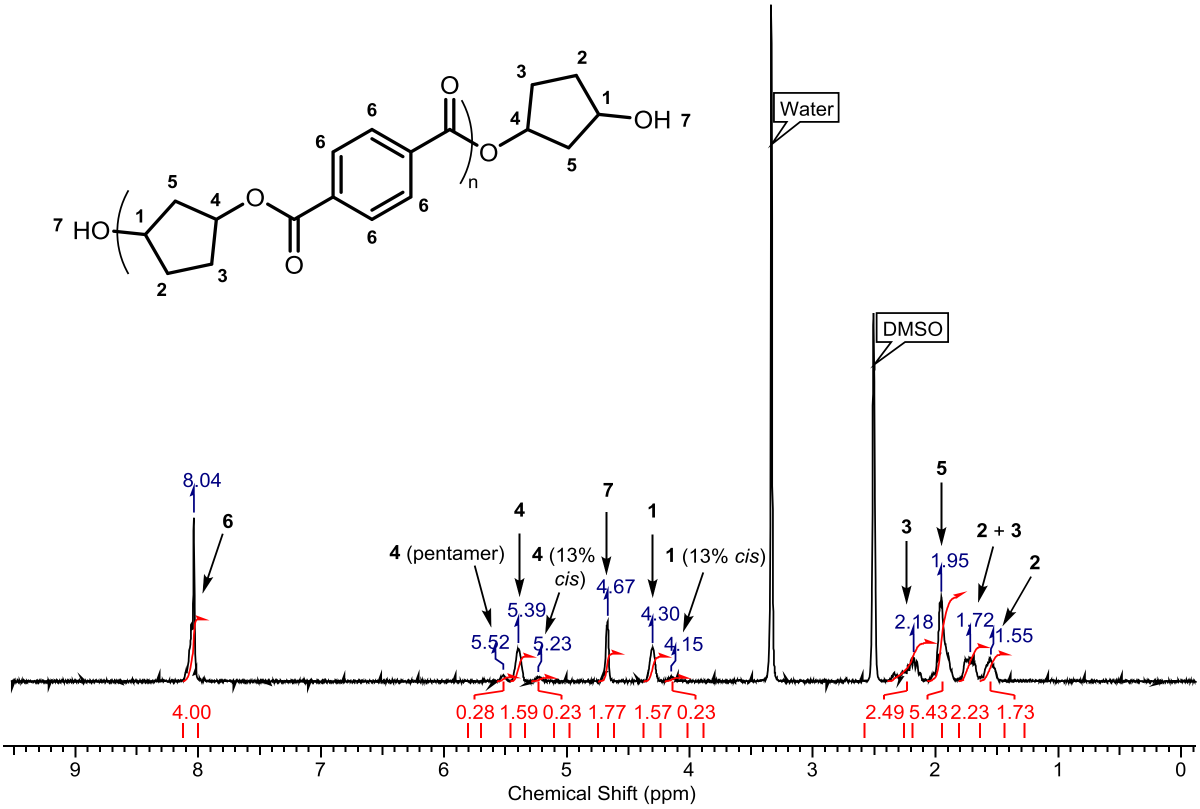

Figure 8. ¹H-NMR analysis of trimer 1,3-cyclopentanediol-terephthalate.

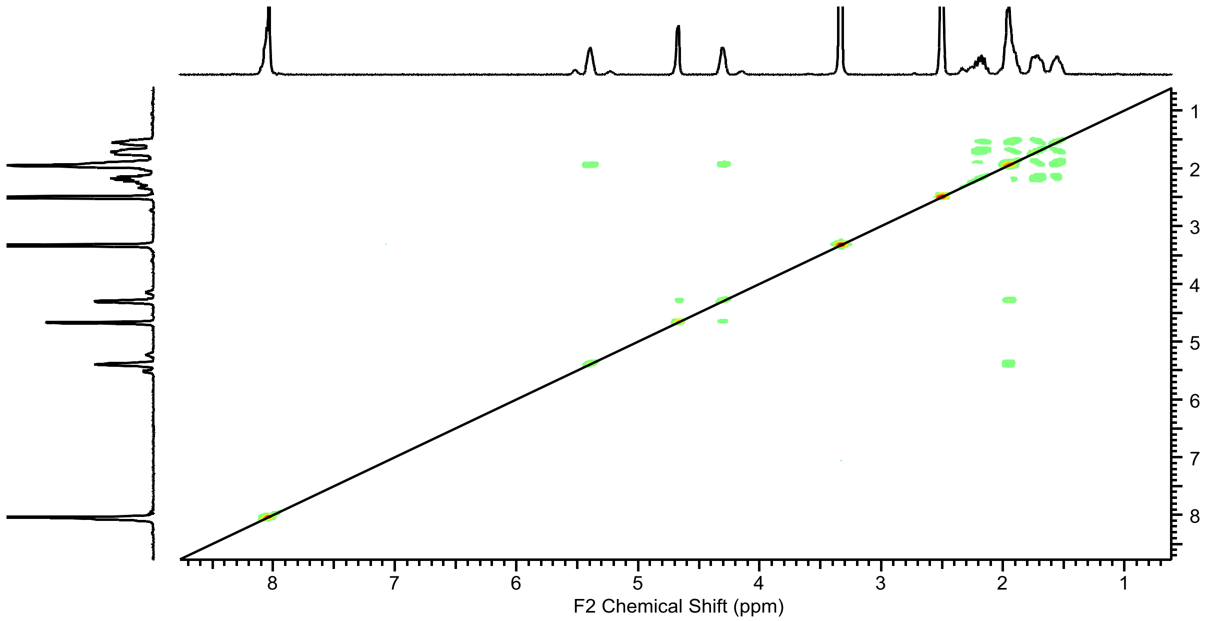

Figure 9. COSY (^{1}H-^{1}H) NMR analysis of trimer 1,3-cyclopentanediol-terephthalate.

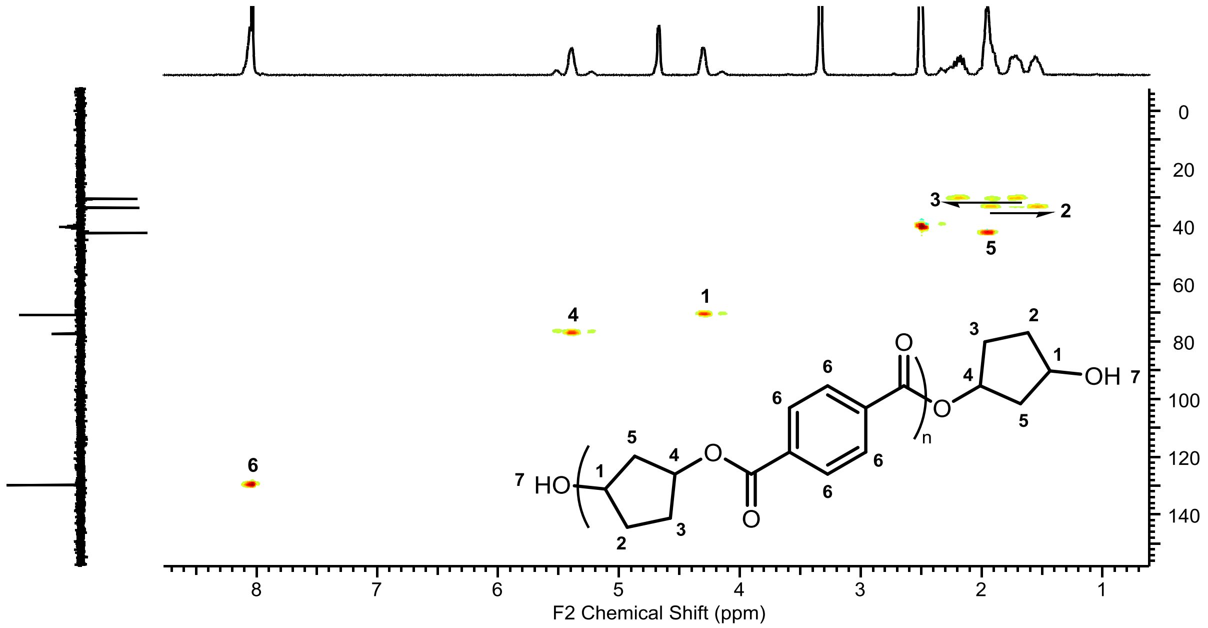

Figure 10. HSQC (^{1}H-APT) NMR analysis of trimer 1,3-cyclopentanediol-terephthalate.

NMR analysis of trimer 1,3-cyclopentanediol-furanoate – 8% *cis*

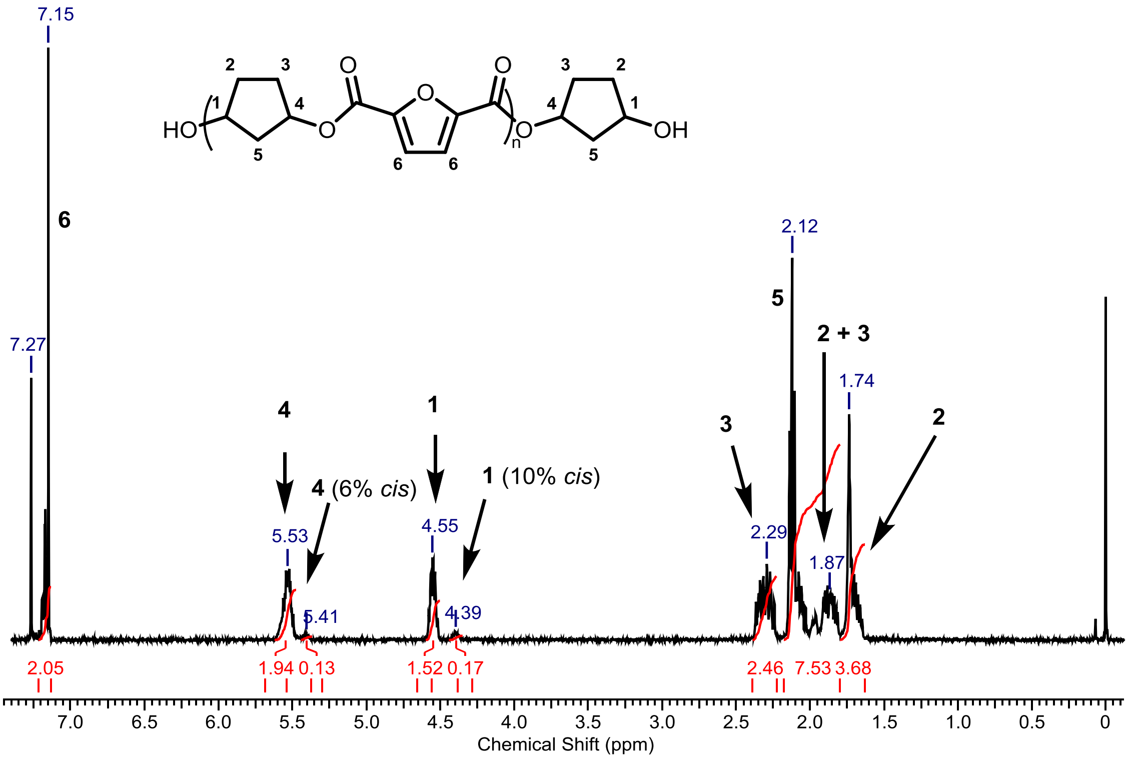

Figure 11. **^{1}H-NMR analysis of trimer 1,3-cyclopentanediol-furanoate – 8% *cis*.**

NMR analysis of trimer 1,3-cyclopentanediol-furanoate – 20% *cis*

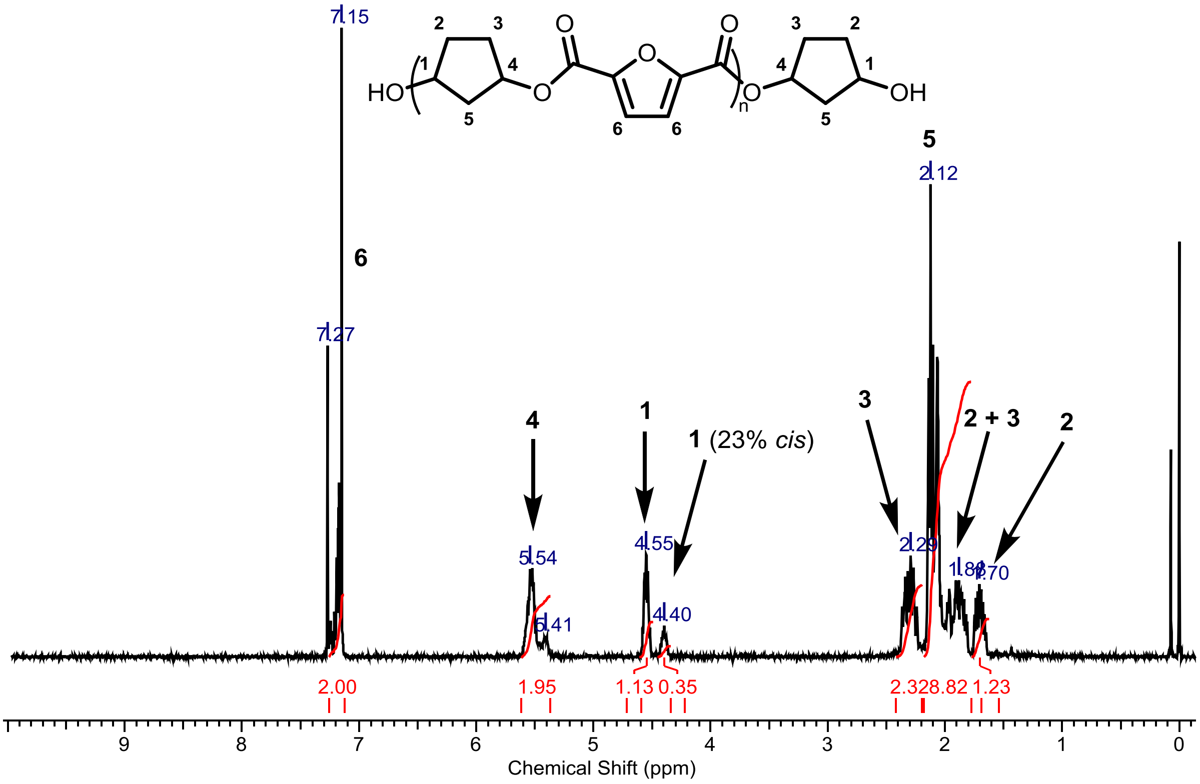

Figure 12. **^{1}H-NMR analysis of trimer 1,3-cyclopentanediol-furanoate – 20% *cis*.**

NMR analysis of trimer 1,3-cyclopentanediol-furanoate – 30% *cis*

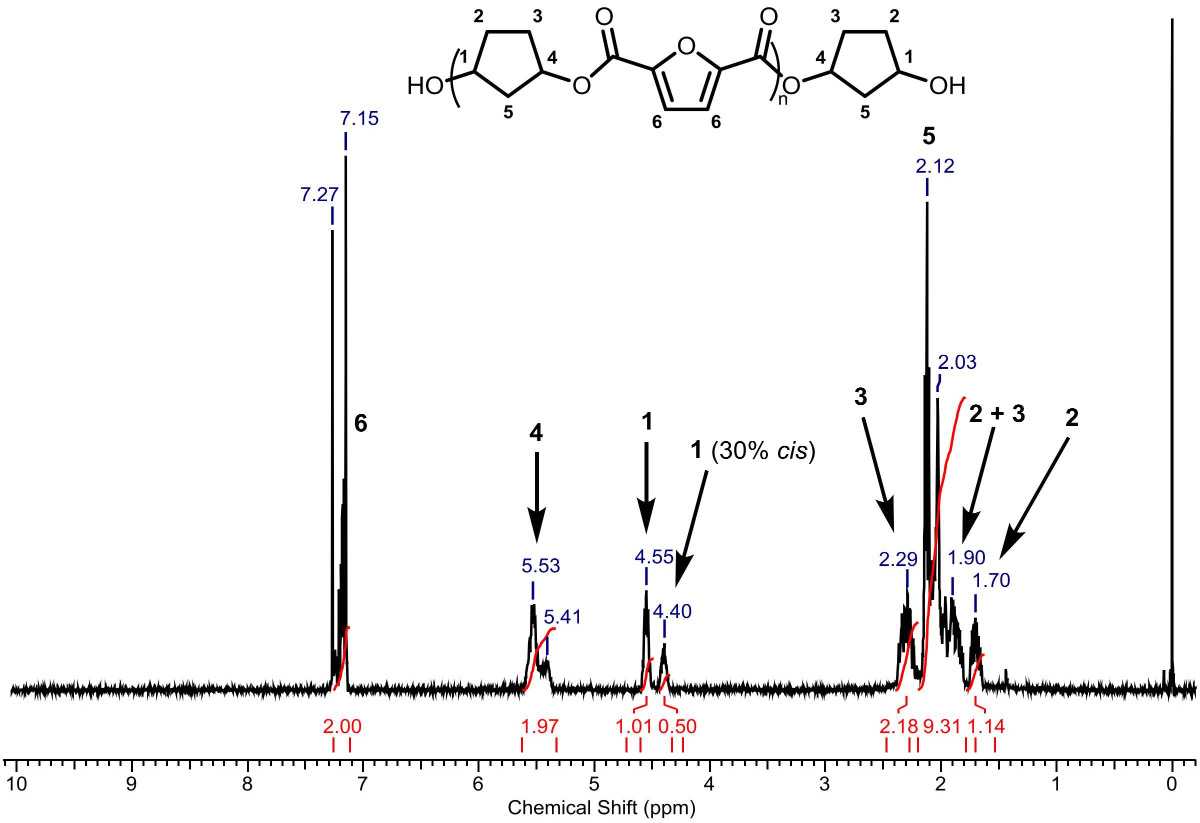

Figure 13. ^{1}**H-NMR analysis of trimer 1,3-cyclopentanediol-furanoate – 30% *cis*.**

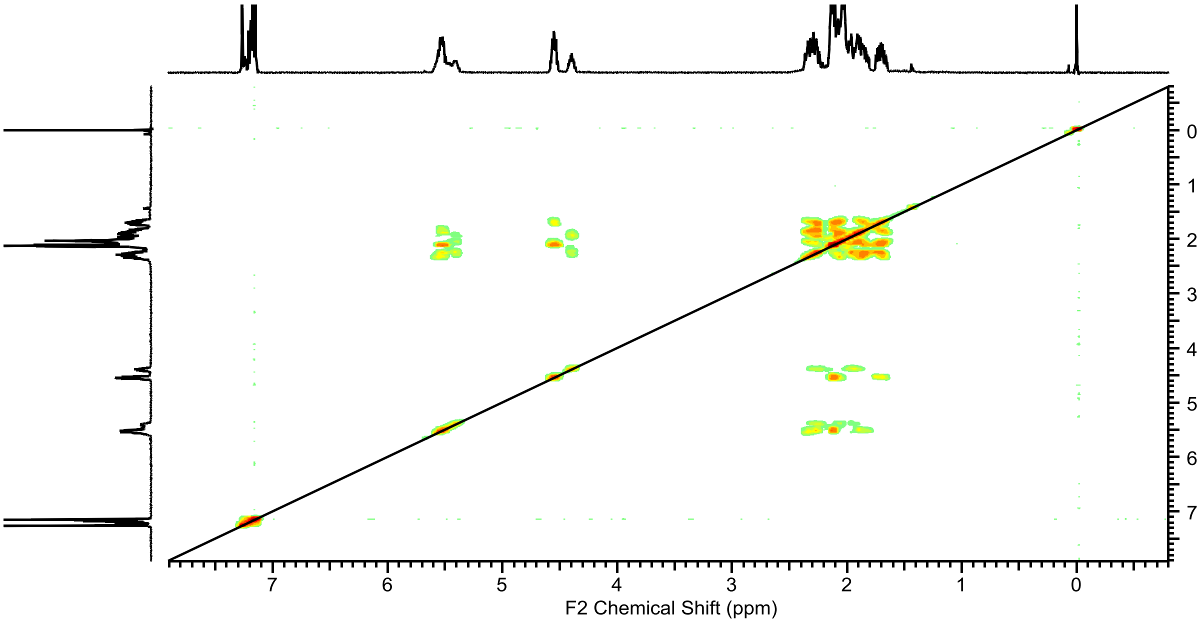

Figure 14. COSY (^{1}H-^{1}H) NMR analysis of trimer 1,3-cyclopentanediol-furanoate – 30% *cis*.

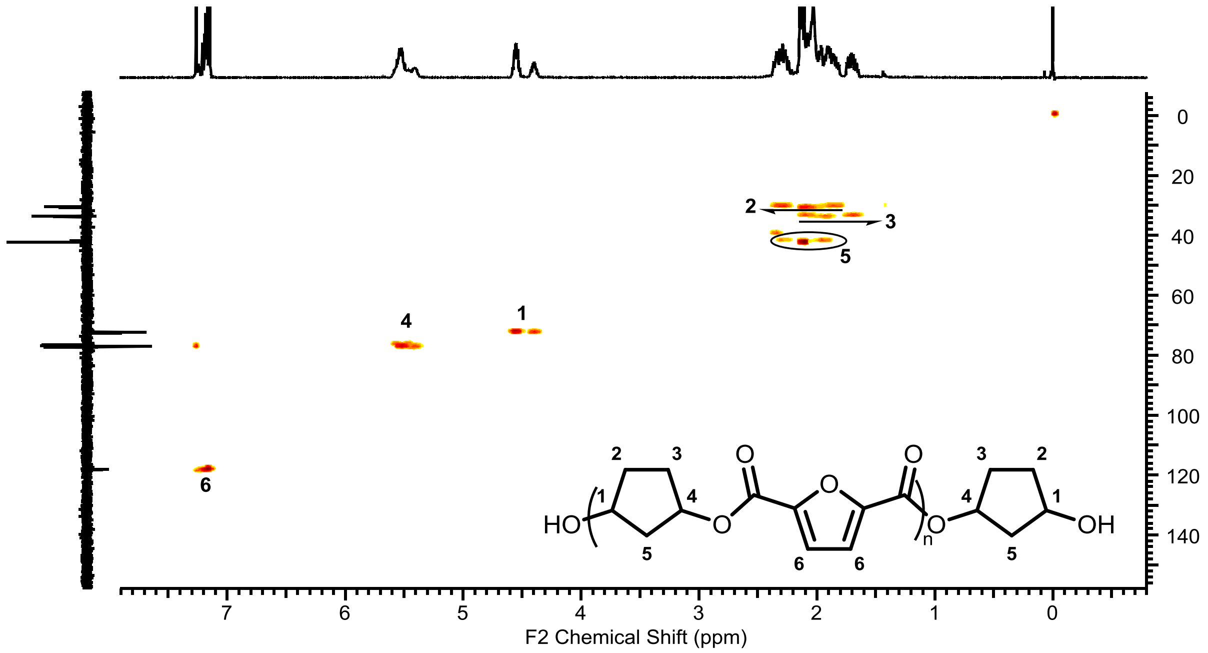

Figure 15. HSQC (^{1}H-DEPT) NMR analysis of trimer 1,3-cyclopentanediol-furanoate – 30% *cis*.

NMR analysis of trimer 1,3-cyclopentanediol-furanoate – 40% *cis*

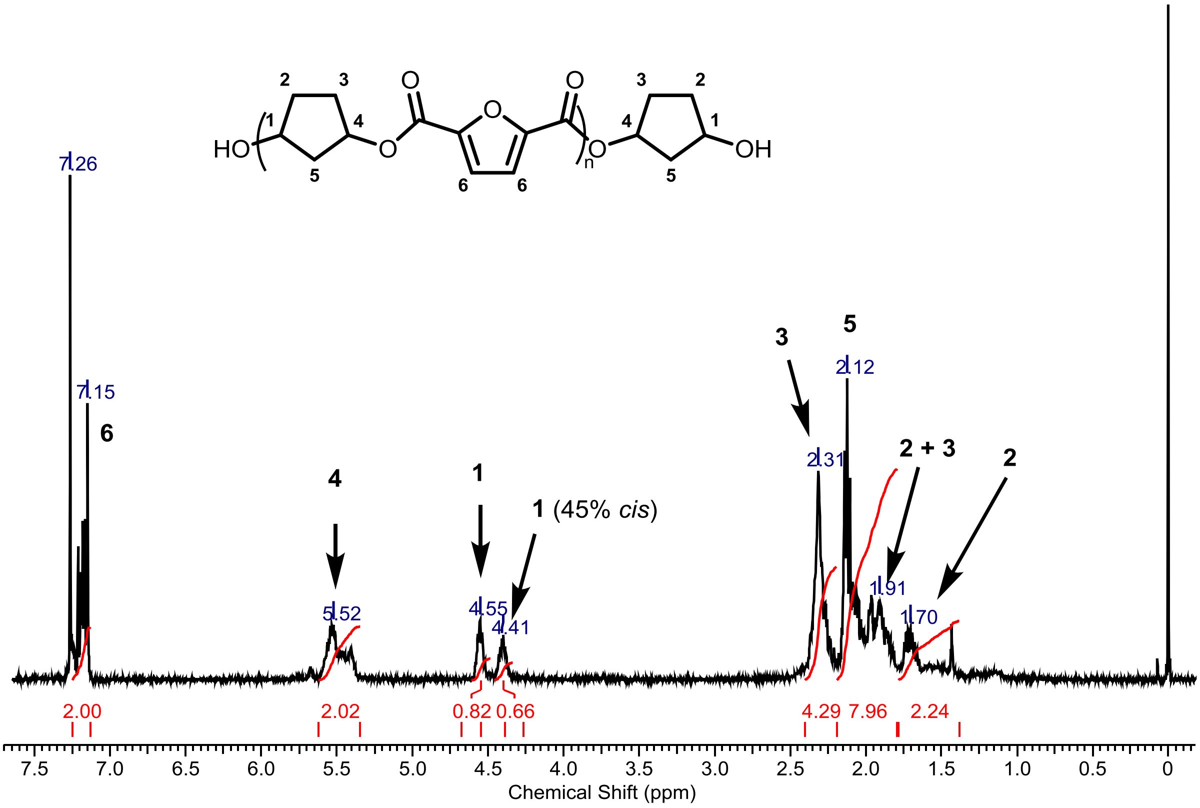

Figure 16. ^{1}H-NMR analysis of trimer 1,3-cyclopentanediol-furanoate – 40% *cis*.

NMR analysis of trimer 1,3-cyclopentanediol-adipate

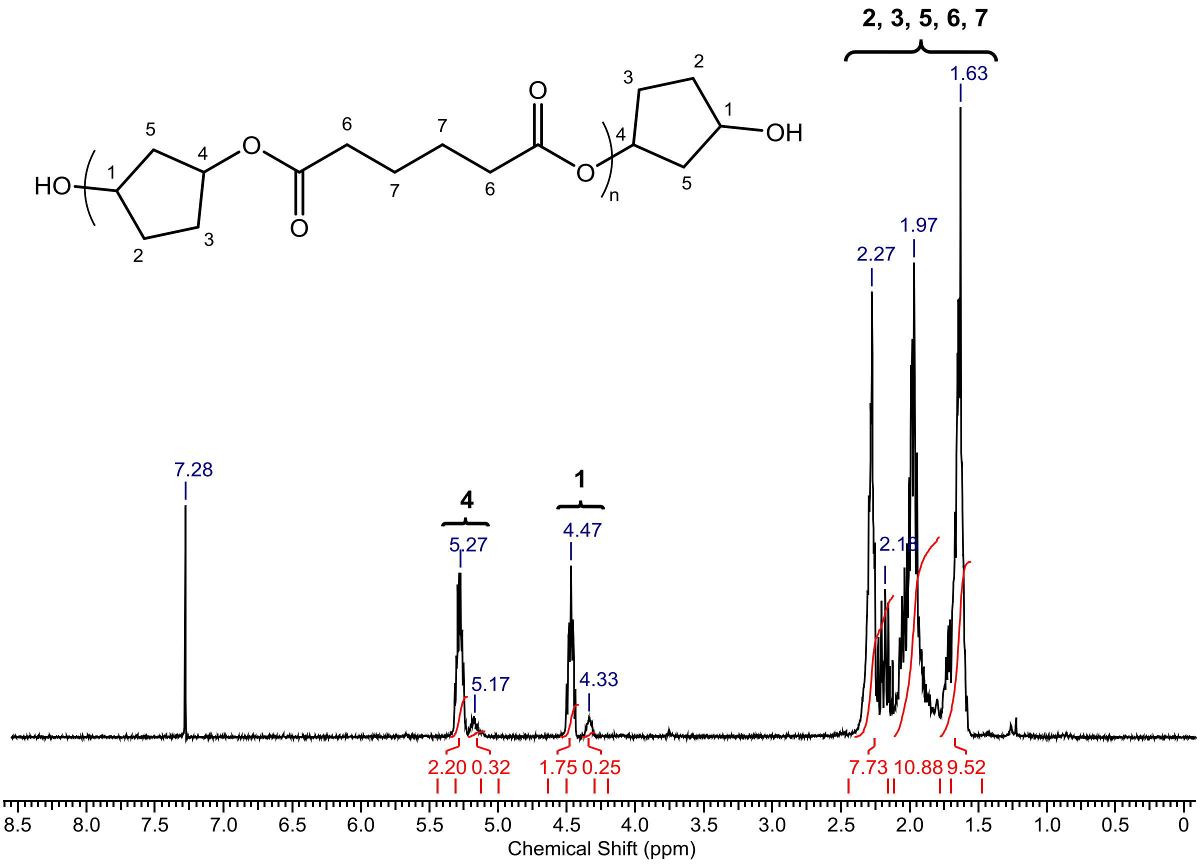

Figure 17. ^{1}H-NMR analysis of trimer 1,3-cyclopentanediol-adipate.

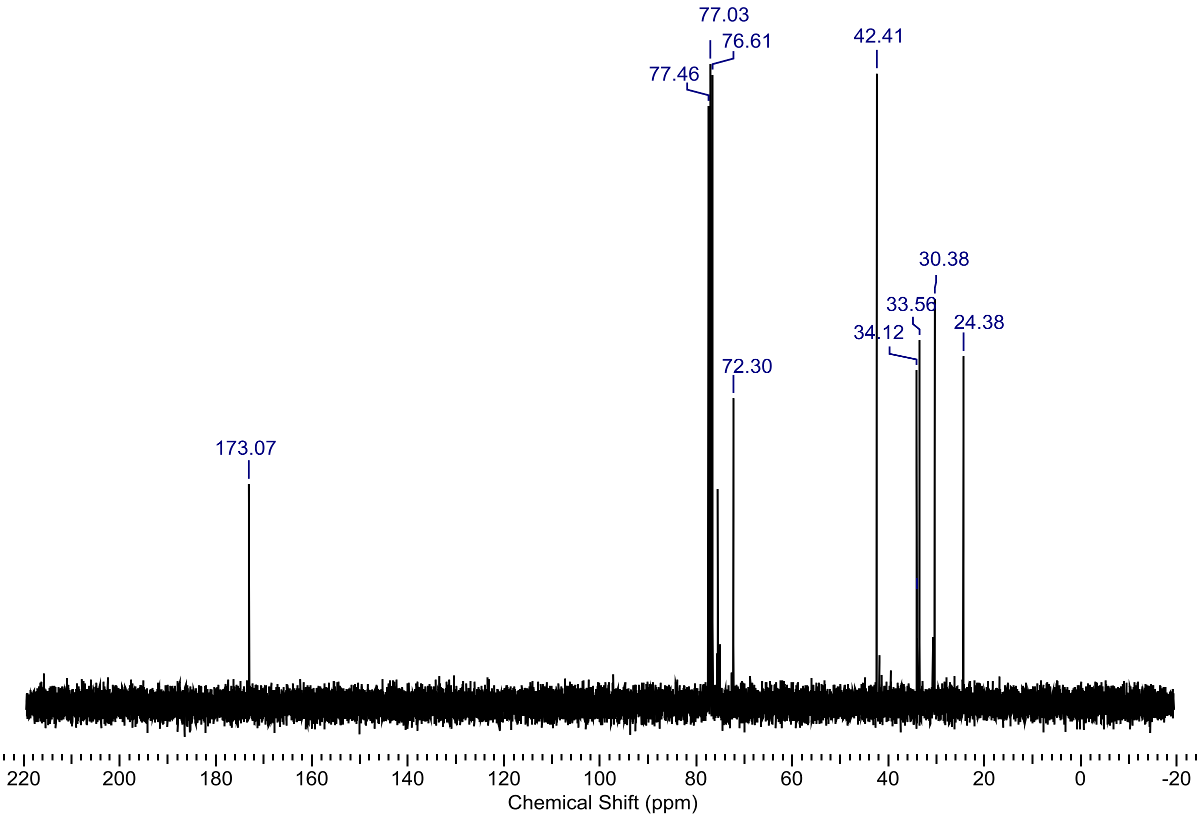

Figure 18. ^{13}C-NMR analysis of trimer 1,3-cyclopentanediol-adipate.

NMR analysis of trimer 1,3-cyclopentanediol-sebacate

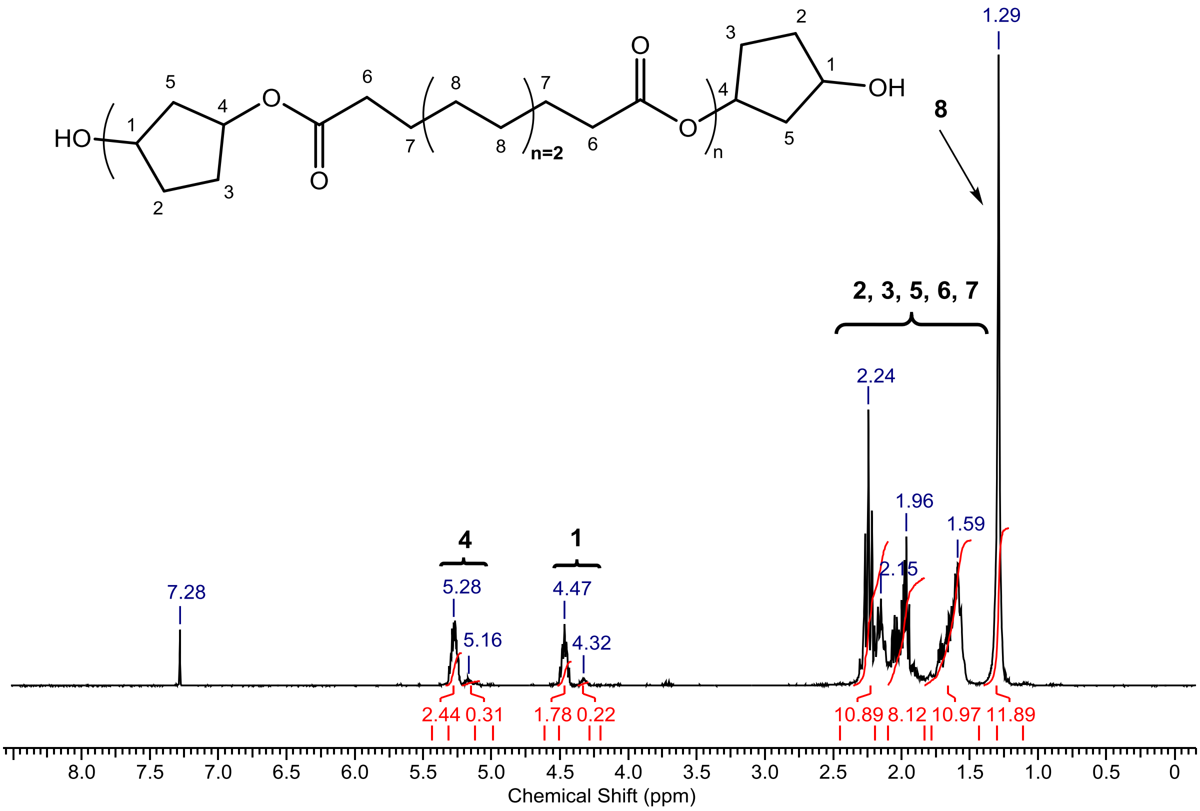

Figure 19. ^{1}**H-NMR analysis of trimer 1,3-cyclopentanediol-sebacate.**

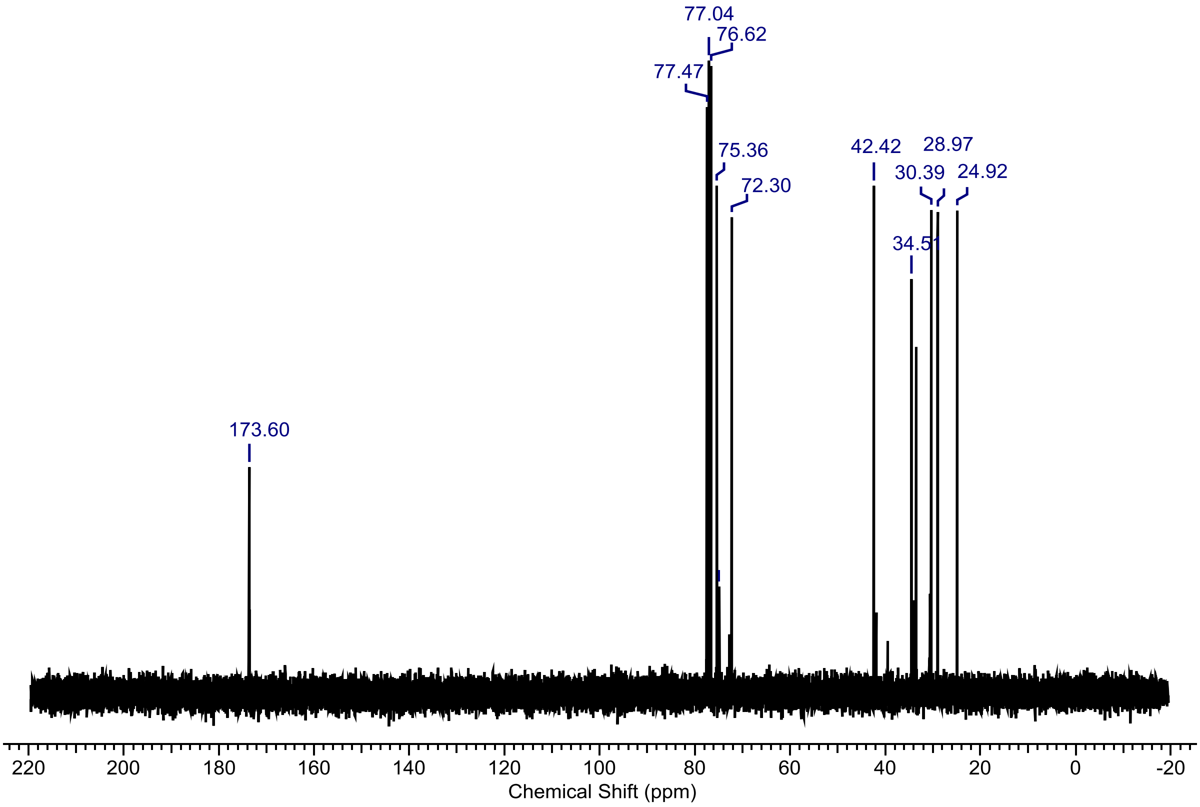

Figure 20. ^{13}**C-NMR analysis of trimer 1,3-cyclopentanediol-sebacate.**

NMR analysis of trimer 1,4-cyclohexanediol-terephthalate

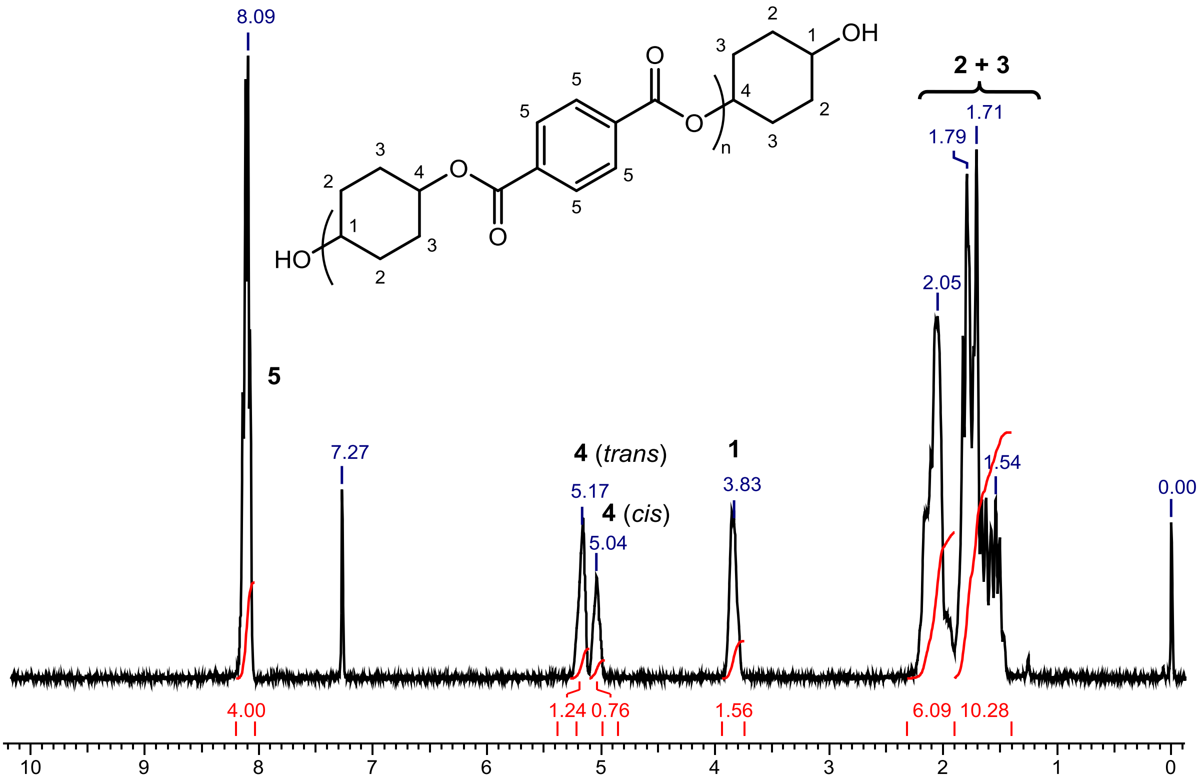

Figure 21. **^{1}H-NMR analysis of trimer 1,4-cyclohexanediol-terephthalate.**

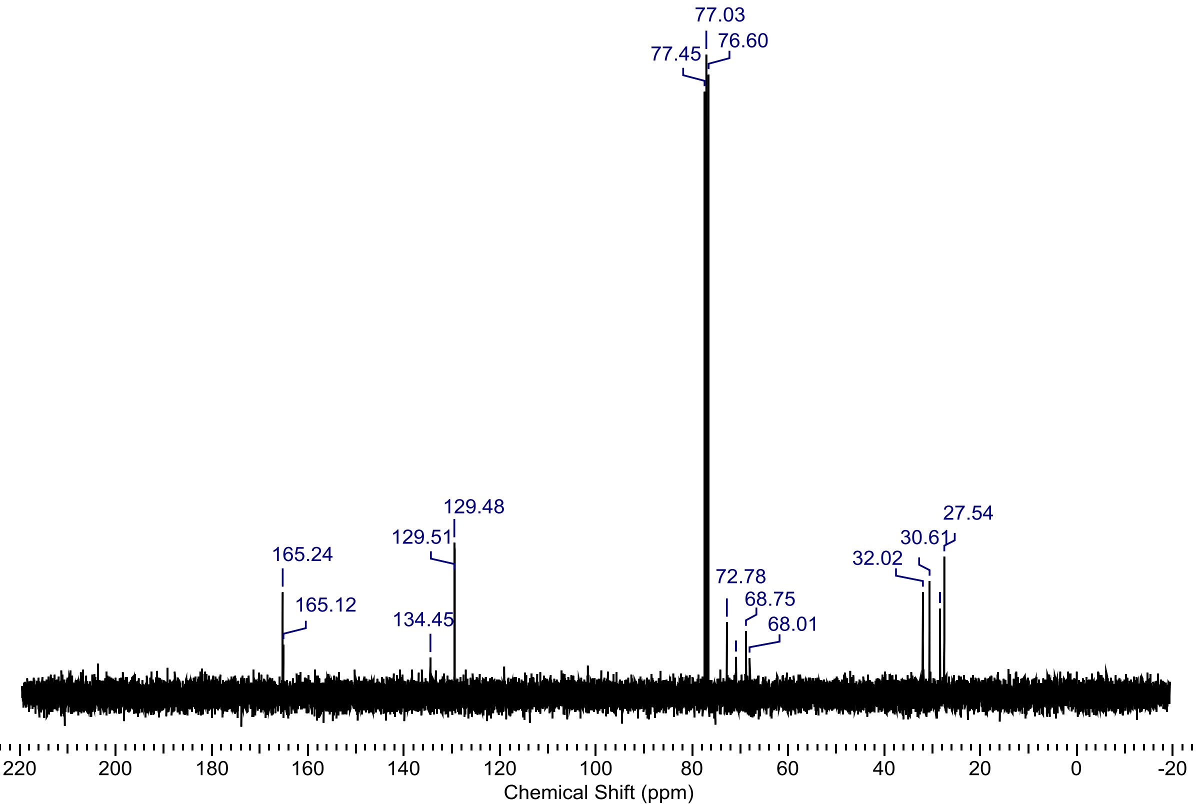

Figure 22. **^{13}C-NMR analysis of trimer 1,4-cyclohexanediol-terephthalate.**

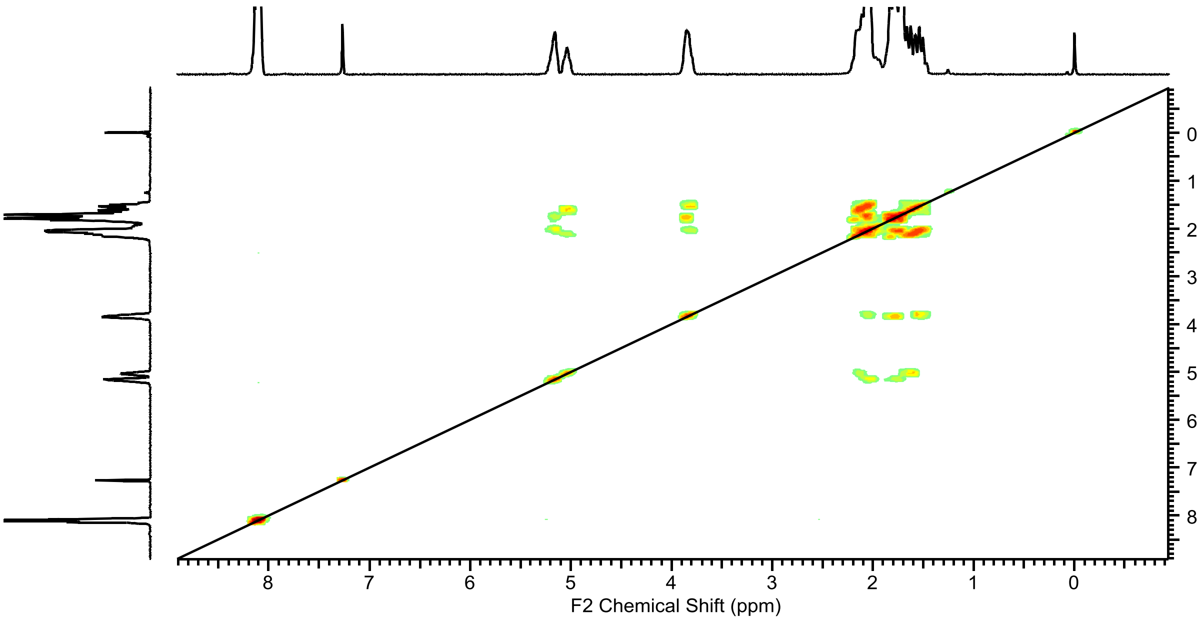

Figure 23. COSY (^{1}H-^{1}H) NMR analysis of trimer 1,4-cyclohexanediol-terephthalate.

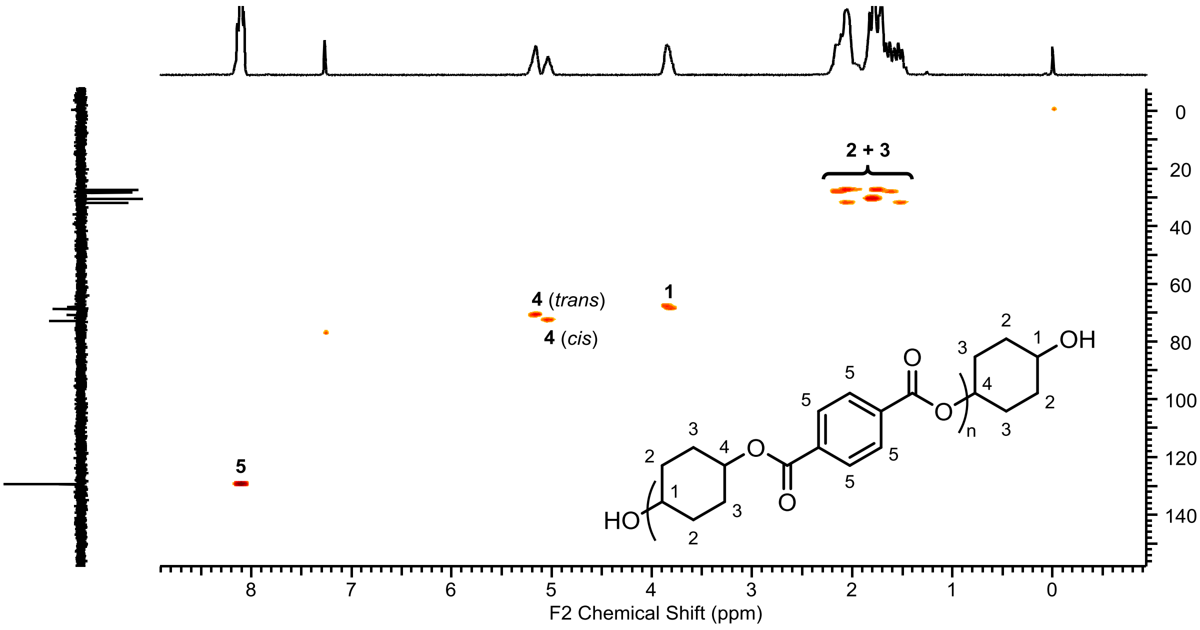

Figure 24. HSQC (^{1}H-DEPT) NMR analysis of trimer 1,4-cyclohexanediol-terephthalate.

NMR analysis of trimer 1,4-cyclohexanediol-adipate

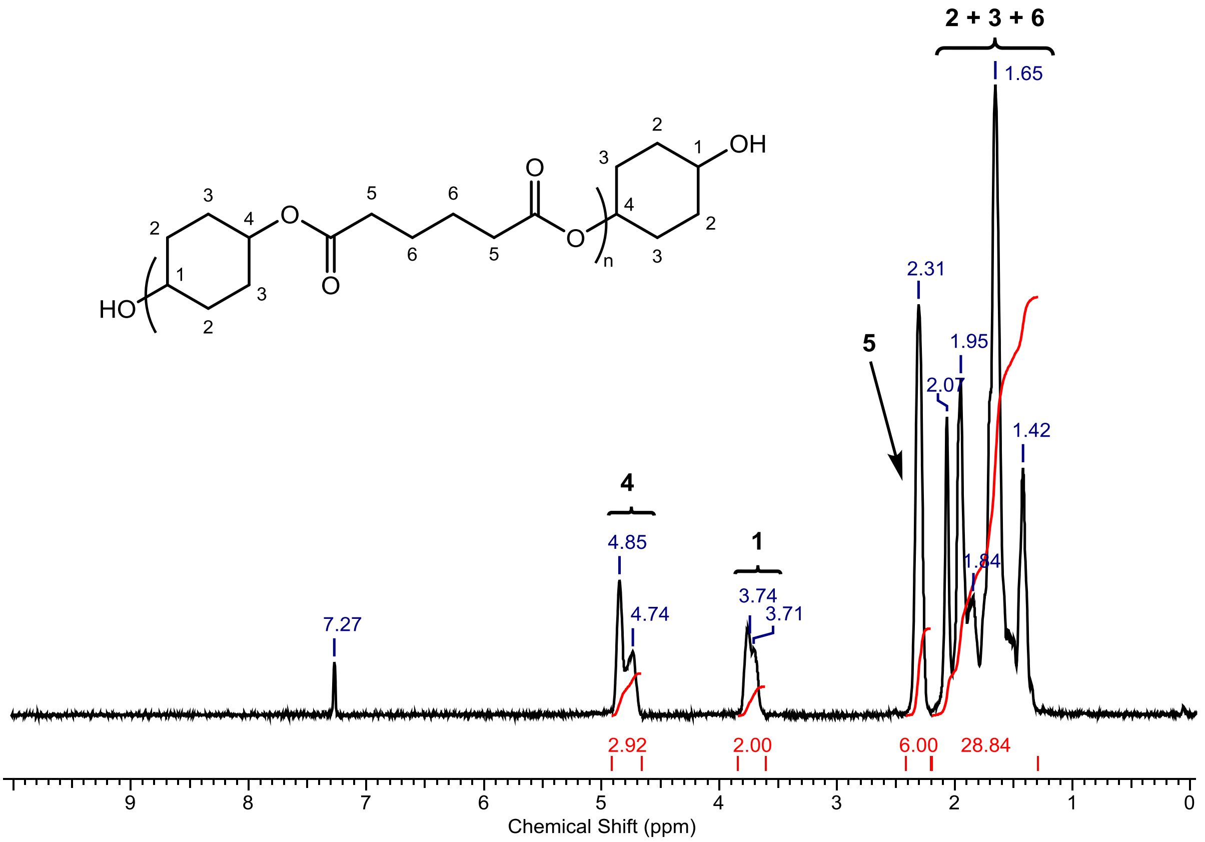

Figure 25. ^{1}H-NMR analysis of trimer 1,4-cyclohexanediol-adipate.

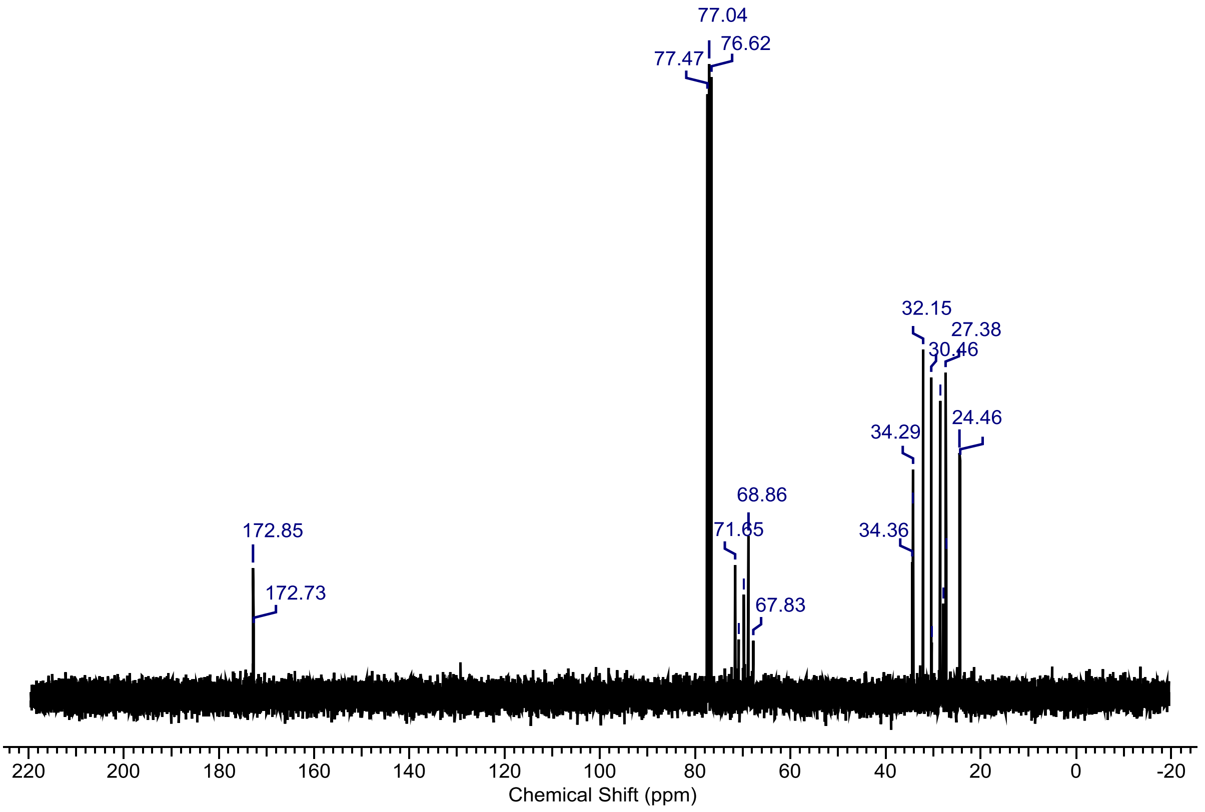

Figure 26. ^{13}C-NMR analysis of trimer 1,4-cyclohexanediol-adipate.

NMR analysis of trimer 1,4-cyclohexanediol-sebacate

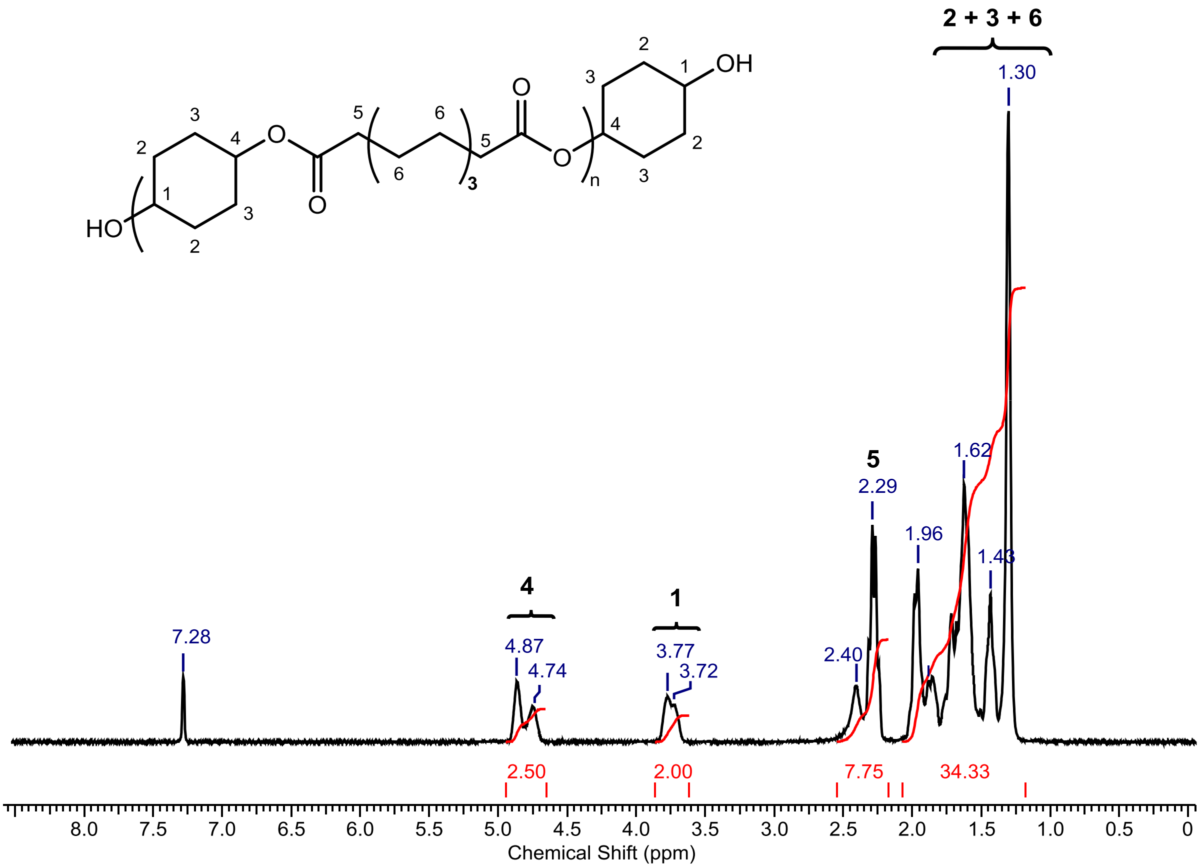

Figure 27. ^{1}H-NMR analysis of trimer 1,4-cyclohexanediol-sebacate.

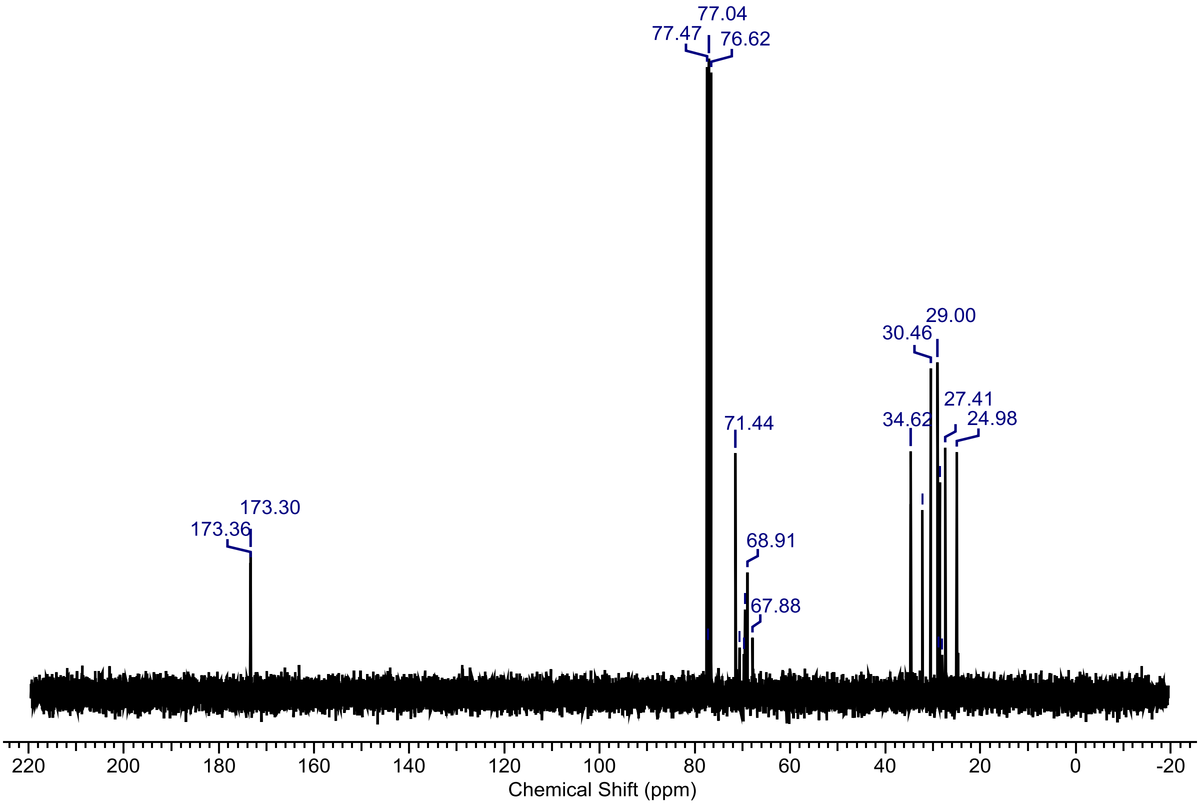

Figure 28. ^{13}C-NMR analysis of trimer 1,4-cyclohexanediol-sebacate.

NMR analysis of trimer 1,4-cyclohexanedimethanol-terephthalate

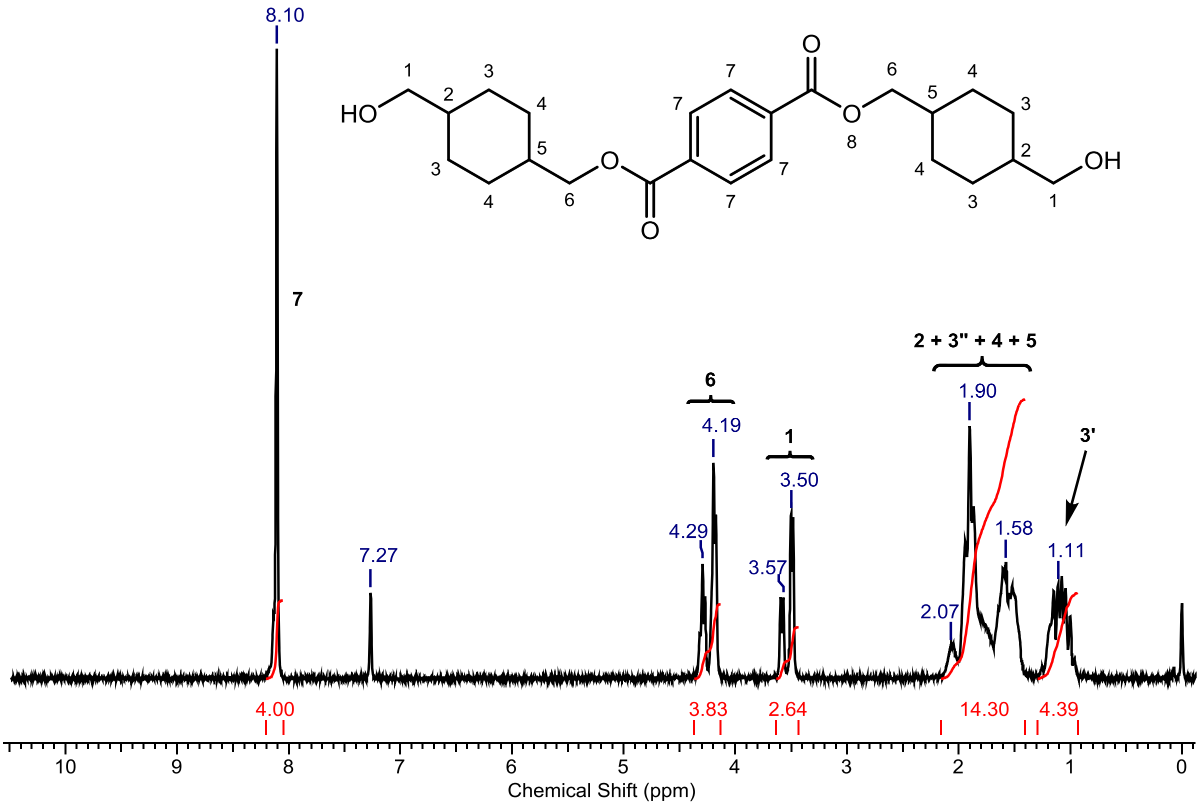

Figure 29. ^{1}**H-NMR analysis of trimer 1,4-cyclohexanedimethanol-terephthalate.**

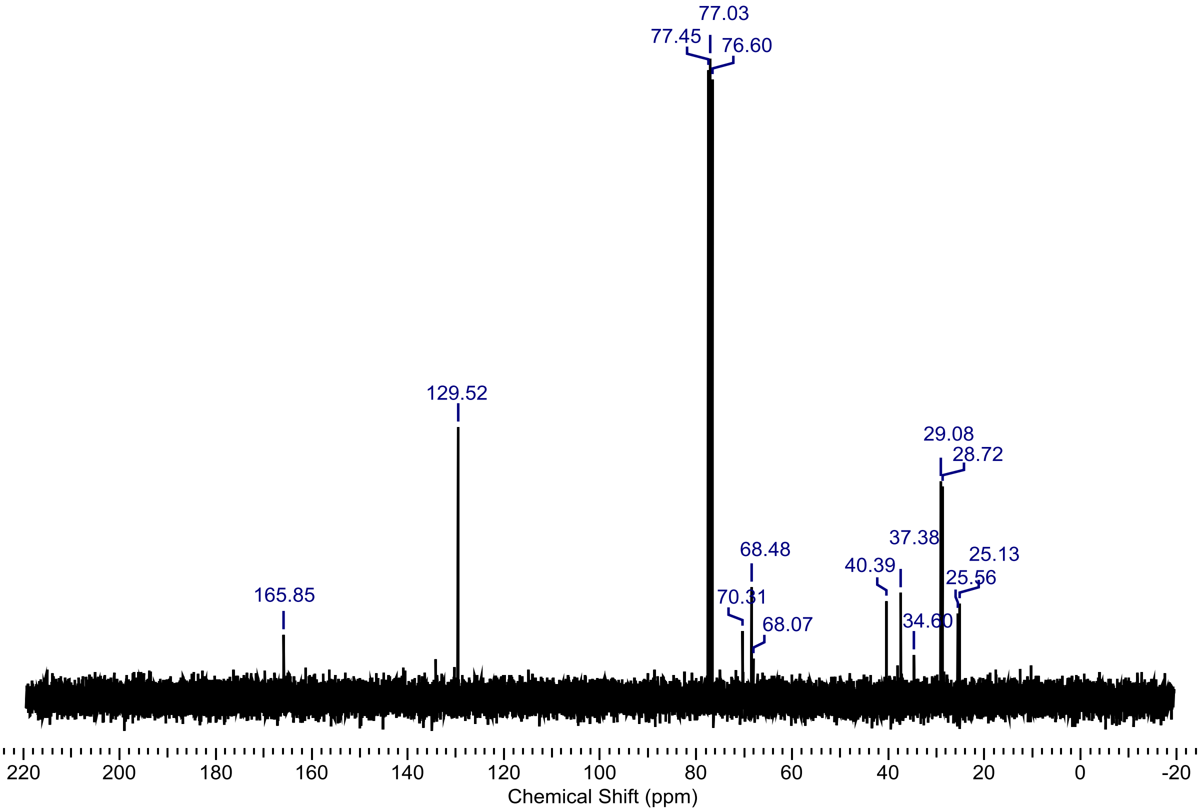

Figure 30. ^{13}**C-NMR analysis of trimer 1,4-cyclohexanedimethanol-terephthalate.**

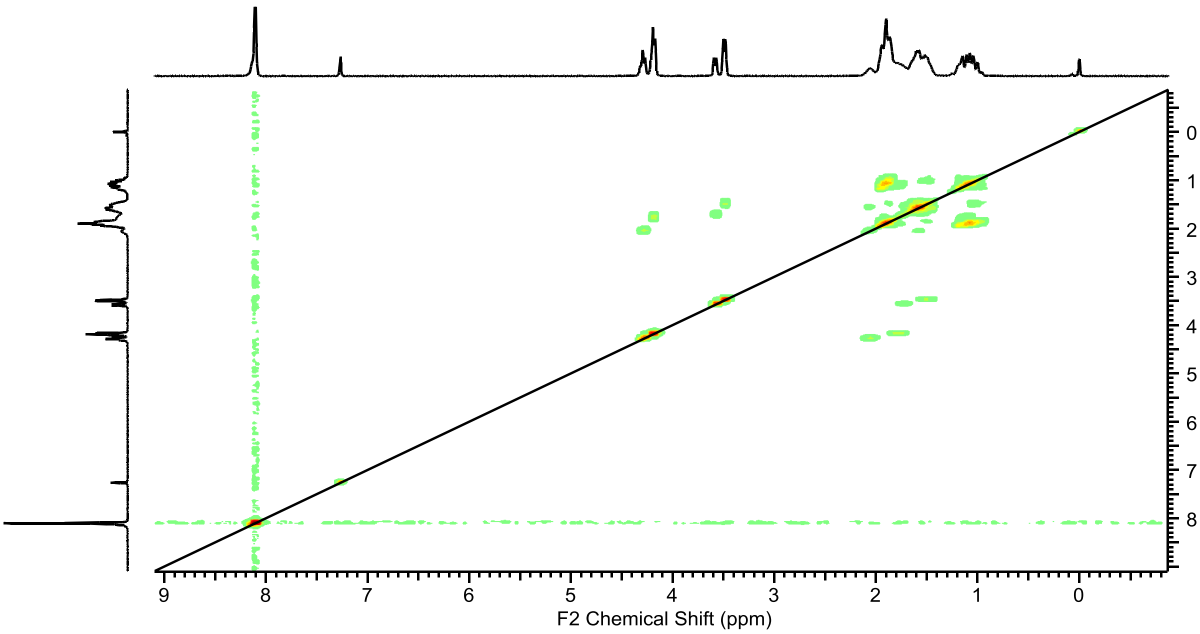

Figure 31. COSY (^{1}H-^{1}H) NMR analysis of trimer 1,4-cyclohexanedimethanol-terephthalate.

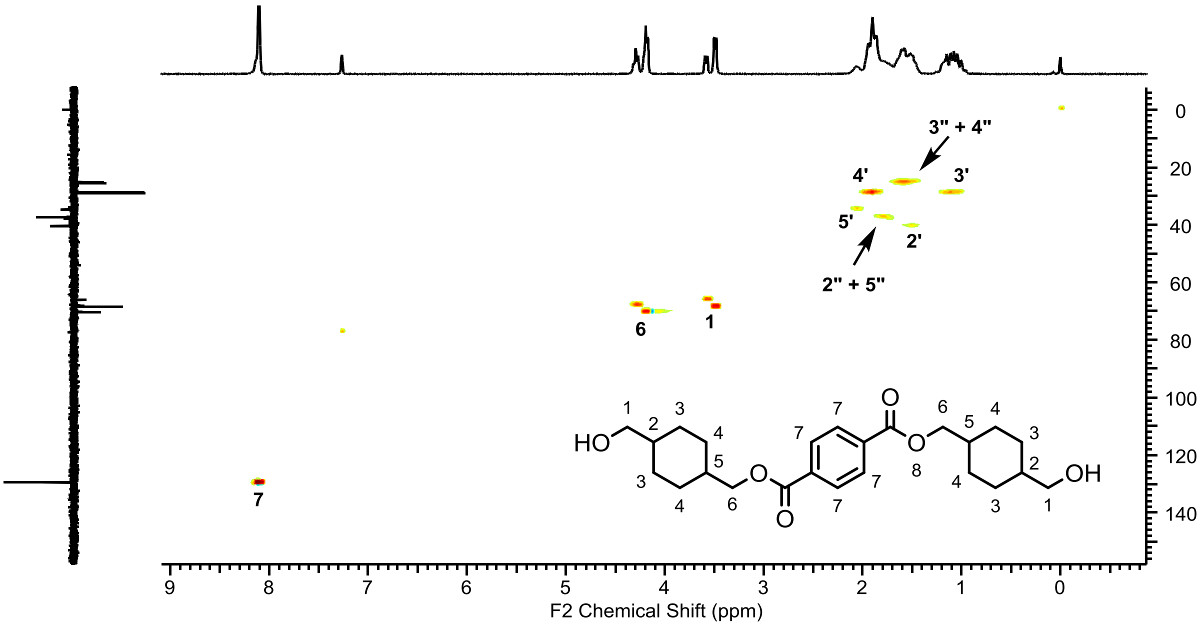

Figure 32. HSQC (^{1}H-DEPT) NMR analysis of trimer 1,4-cyclohexanedimethanol-terephthalate.

NMR analysis of trimer 1,4-cyclohexanedimethanol-adipate

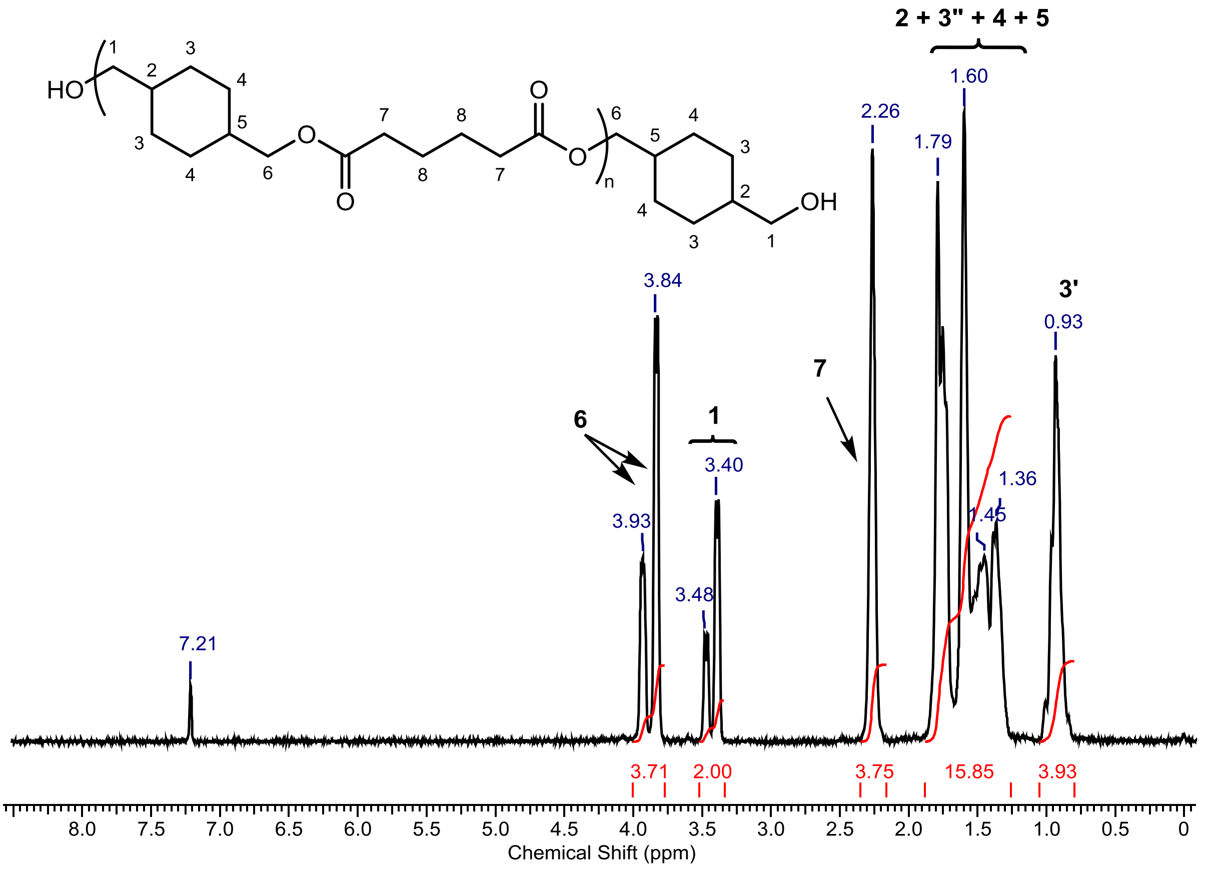

Figure 33. ^{1}H-NMR analysis of trimer 1,4-cyclohexanedimethanol-adipate.

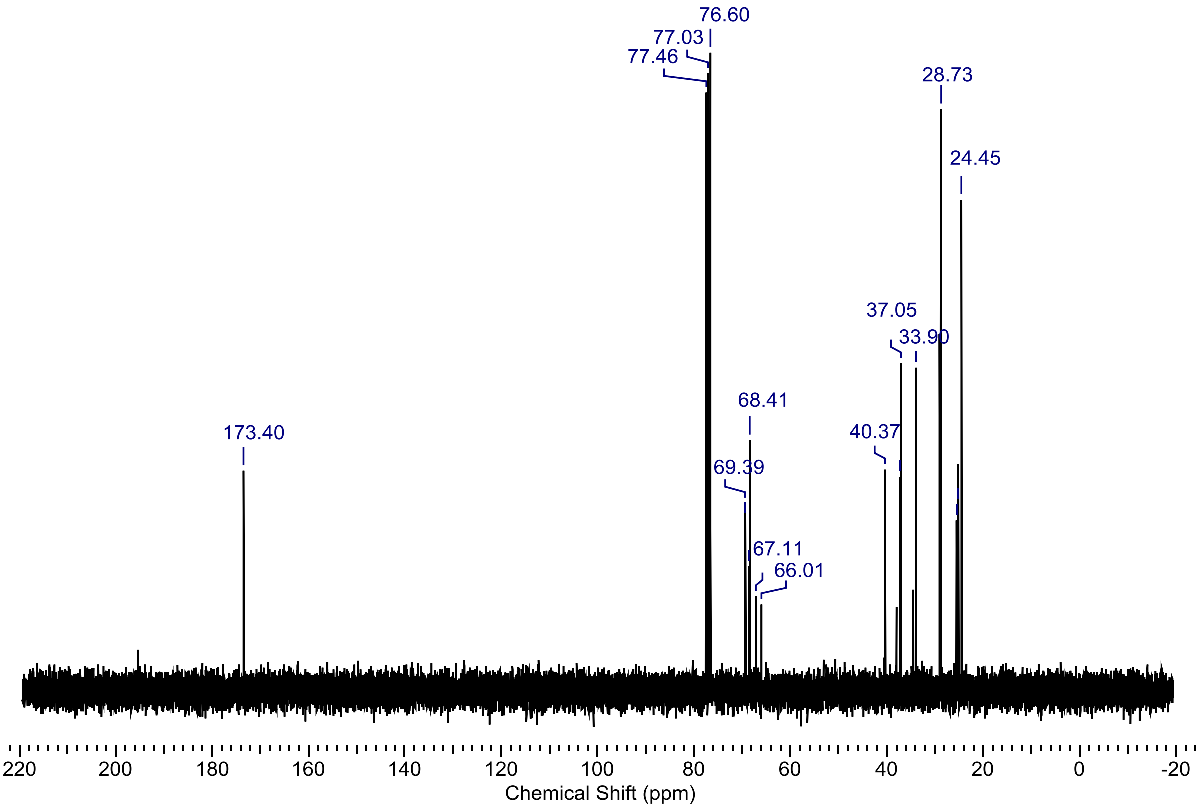

Figure 34. ^{13}C-NMR analysis of trimer 1,4-cyclohexanedimethanol-adipate.

NMR analysis of trimer 1,4-cyclohexanedimethanol-sebacate

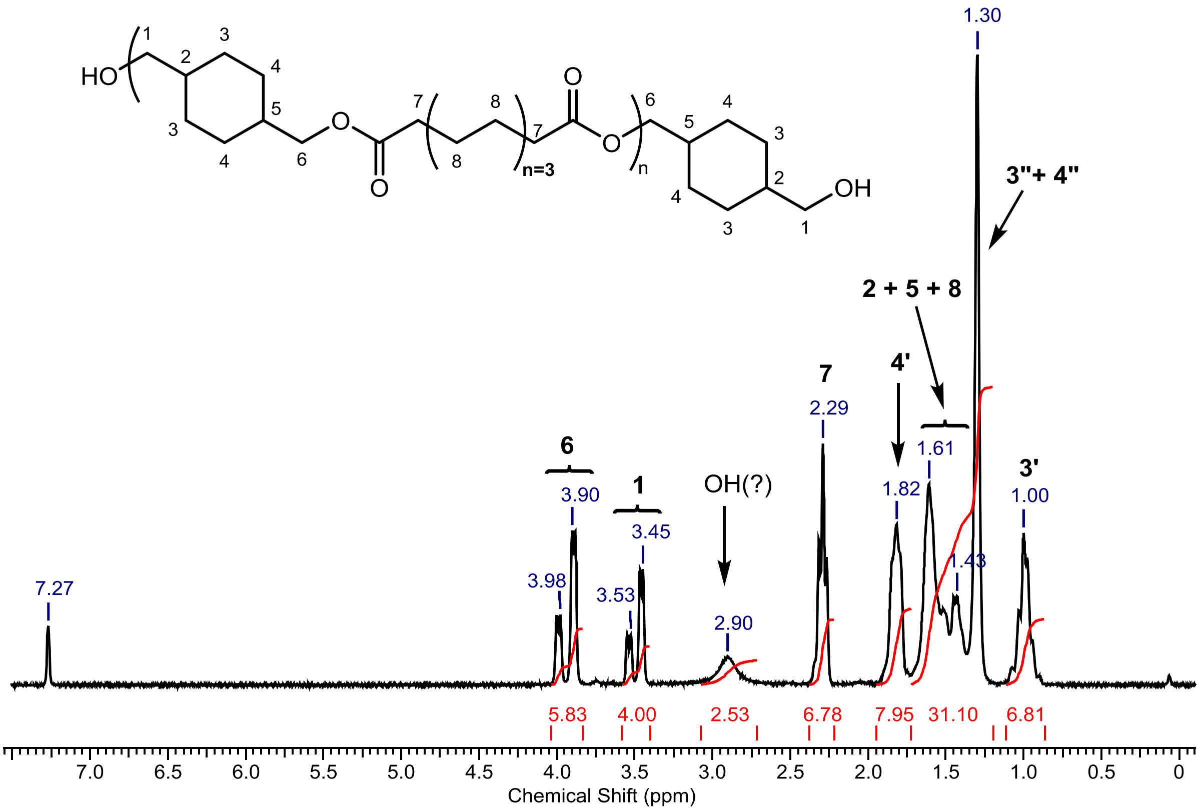

Figure 35. ¹H-NMR analysis of trimer 1,4-cyclohexanedimethanol-sebacate.

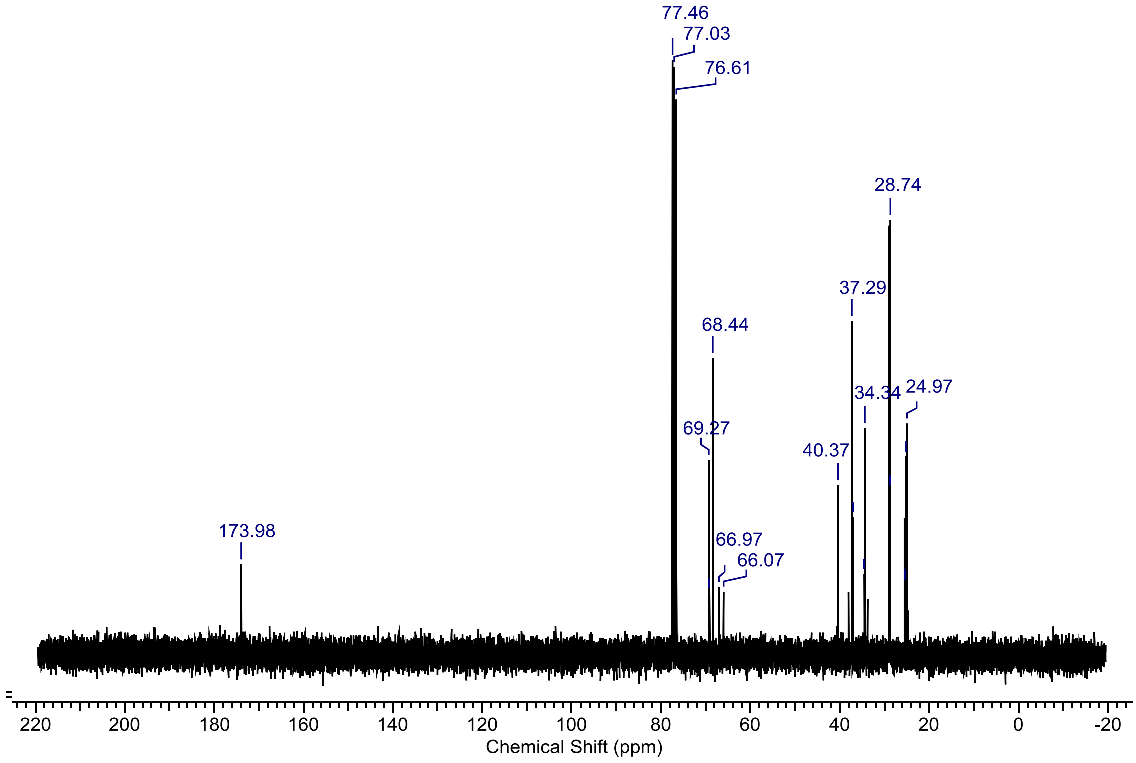

Figure 36. ¹³C-NMR analysis of trimer 1,4-cyclohexanedimethanol-sebacate.

Detailed polymerization of small-scale screening

Table 3. Details of polymerization of trimer erthyleneglycol-terephthalate for validation of consistency of small-scale reactor.

Monomer	run	Cat	Cat (mol%)	Weight input	Weight after rxn	weight loss %	theoretical	color	M_n	M_w	Đ
E-T	1	Ti(IV)butoxide	1.0%	10.0	8.0	20%	24%	white	17340	77780	4.49
E-T	1	Ti(IV)butoxide	1.0%	9.0	6.0	33%	24%	white	19590	95900	4.90
E-T	1	Ti(IV)butoxide	1.0%	10.0	7.0	30%	24%	white	17710	74210	4.19
E-T	1	Ti(IV)butoxide	1.0%	8.0	6.0	25%	24%	white	18790	82990	4.42
E-T	1	Ti(IV)butoxide	1.0%	10.0	8.0	20%	24%	white	19120	85210	4.46
E-T	1	Ti(IV)butoxide	1.0%	13.0	11.0	15%	24%	white	15550	81800	5.26
E-T	1	Ti(IV)butoxide	1.0%	12.0	10.0	17%	24%	white	15830	78690	4.97
E-T	1	Ti(IV)butoxide	1.0%	11.0	8.0	27%	24%	white	17140	85710	5.00
E-T	2	Ti(IV)butoxide	1.0%	10.4	7.7	26%	24%	white	12900	34970	2.71
E-T	2	Ti(IV)butoxide	1.0%	9.8	6.9	30%	24%	white	16160	46640	2.89
E-T	2	Ti(IV)butoxide	1.0%	11.0	9.0	18%	24%	white	18730	72100	3.85

Table 4. Details of polymerization of trimer ethyleneglycol-terephthalate for study of effect on monomer loading in small-scale reactor.

Monomer	Cat	Cat (mol%)	Weight input	Weight after rxn	weight loss %	theoretical	color	M_n	M_w	Đ
E-T	Ti(IV)butoxide	1.0%	1.2	0.5	58%	24%	white	24990	74310	2.97
E-T	Ti(IV)butoxide	1.0%	1.1	0.5	55%	24%	white	26830	72680	2.71
E-T	Ti(IV)butoxide	1.0%	2.1	1.4	33%	24%	white	23040	80450	3.49
E-T	Ti(IV)butoxide	1.0%	2.2	1.6	27%	24%	white	16600	60200	3.03
E-T	Ti(IV)butoxide	1.0%	3.0	2.0	33%	24%	white	19980	63270	3.17
E-T	Ti(IV)butoxide	1.0%	3.0	2.0	33%	24%	white	25150	78290	3.11
E-T	Ti(IV)butoxide	1.0%	4.2	2.7	36%	24%	white	19740	64850	3.29
E-T	Ti(IV)butoxide	1.0%	4.0	2.6	35%	24%	white	24860	71210	2.87
E-T	Ti(IV)butoxide	1.0%	5.0	3.3	34%	24%	white	18590	54270	2.92
E-T	Ti(IV)butoxide	1.0%	5.0	3.3	34%	24%	white	20360	55640	2.73
E-T	Ti(IV)butoxide	1.0%	6.0	3.9	35%	24%	white	18370	58470	3.18
E-T	Ti(IV)butoxide	1.0%	5.9	4.3	27%	24%	white	21420	63600	2.97
E-T	Ti(IV)butoxide	1.0%	7.0	5.2	26%	24%	white	15760	47750	3.03
E-T	Ti(IV)butoxide	1.0%	6.8	4.6	32%	24%	white	20360	55260	2.71
E-T	Ti(IV)butoxide	1.0%	8.4	5.9	30%	24%	white	13090	38110	2.91
E-T	Ti(IV)butoxide	1.0%	8.1	6.0	26%	24%	white	17200	44460	2.59
E-T	Ti(IV)butoxide	1.0%	8.9	6.7	25%	24%	white	14820	42080	2.84
E-T	Ti(IV)butoxide	1.0%	9.2	7.0	24%	24%	white	15360	46910	3.05
E-T	Ti(IV)butoxide	1.0%	10.4	7.7	26%	24%	white	12900	34970	2.71
E-T	Ti(IV)butoxide	1.0%	9.8	6.9	30%	24%	white	16160	46640	2.89
E-T	Ti(IV)butoxide	1.0%	11.0	9.0	18%	24%	white	18730	72100	3.85
E-T	Ti(IV)butoxide	1.0%	19.0	16.0	16%	24%	white	9669	38660	4.00
E-T	Ti(IV)butoxide	1.0%	50.0	40.0	20%	24%	white	3695	12260	3.32
E-T	Ti(IV)butoxide	1.0%	101.0	81.0	20%	24%	white	1613	4527	2.81

Table 5. Details of polymerization with 1,3-cyclopentanediol in the small-scale reactor.

monomer	Cat	cat (mol%)	Weight input	Weight after rxn	weight loss %	theoretical weight loss	color	M_n	M_w	Đ	T_g (°C)	T_m (°C)	T_c (°C)
CPdiol-T	-		9.2	3.6	61%	31%	opaque	1035	1266	1.22			
CPdiol-T	-		9.8	4.7	52%	31%	opaque	1033	1259	1.22			
CPdiol-T	-		10.0	5.0	50%	31%	opaque				n.o.	135.96	75
CPdiol-T	Ti(IV)butoxide	1.0%	9.7	7.8	20%	31%	white	5234	10812	2.07			
CPdiol-T	Ti(IV)butoxide	1.0%	10.9	8.9	18%	31%	white	5341	10849	2.03			
CPdiol-T	Ti(IV)butoxide	1.0%	9.5	7.6	20%	31%	white				n.o.	244.96	203.68
CPdiol-T	Sn(II)Octanoate	1.0%	9.8	7.4	24%	31%	white	3803	6711	1.76			
CPdiol-T	Sn(II)Octanoate	1.0%	9.6	7.0	27%	31%	white	3553	6358	1.79			
CPdiol-T	Sn(II)Octanoate	1.0%	10.0	7.6	24%	31%	white				n.o.	n.o.	n.o.
CPdiol-A	-		11.8	6.8	42%	32%	clear light brown	1244	1697	1.36			
CPdiol-A	-		10.6	6.1	42%	32%	clear light brown	1276	1792	1.40			
CPdiol-A	-		10.8	6.2	43%	32%	clear light brown				n.o.	n.o.	n.o.
CPdiol-A	Ti(IV)butoxide	1.0%	10.4	8.3	20%	32%	opaque yellow	16183	55419	3.42			
CPdiol-A	Ti(IV)butoxide	1.0%	11.0	8.5	23%	32%	opaque yellow	17389	58078	3.34			
CPdiol-A	Ti(IV)butoxide	1.0%	10.2	7.9	23%	32%	opaque yellow				-30.05	n.o.	n.o.
CPdiol-A	Sn(II)Octanoate	1.0%	10.3	7.6	26%	32%	opaque yellow	15667	39365	2.51			
CPdiol-A	Sn(II)Octanoate	1.0%	10.0	7.3	27%	32%	opaque yellow	15737	45620	2.90			
CPdiol-A	Sn(II)Octanoate	1.0%	9.6	7.1	26%	32%	opaque yellow				-23.62	n.o.	n.o.
CPdiol-S	-		11.6	6.4	45%	28%	opaque	1377	1911	1.39			
CPdiol-S	-		10.5	5.5	48%	28%	opaque	1400	1940	1.39			
CPdiol-S	-		10.4	5.7	45%	28%	opaque				n.o.	n.o.	n.o.
CPdiol-S	Ti(IV)butoxide	1.0%	10.8	9.2	15%	28%	white	25167	93115	3.70			
CPdiol-S	Ti(IV)butoxide	1.0%	10.6	8.6	19%	28%	white	24709	91541	3.70			
CPdiol-S	Ti(IV)butoxide	1.0%	9.9	8.2	17%	28%	white				-37.93	44.2	2.1
CPdiol-S	Sn(II)Octanoate	1.0%	10.4	8.0	23%	28%	white	14333	34792	2.43			
CPdiol-S	Sn(II)Octanoate	1.0%	10.4	8.2	21%	28%	white	14669	35596	2.43			
CPdiol-S	Sn(II)Octanoate	1.0%	10.0	7.8	22%	28%	white				-39.19	43.84	4.42

Table 6. Details of polymerization with 1,4-cyclohexanediol in the small-scale reactor.

monomer	Cat	cat (mol%)	Weight input	Weight after rxn	weight loss %	theoretical weight loss	color	M_n	M_w	Đ	T_g (°C)	T_m (°C)	T_c (°C)	T_{cc} (°C)
CHdiol-T	-		10.0	10.0	0%	32%	white powder	1055	1248	1.18				
CHdiol-T	-		10.2	10.2	0%	32%	white powder	1056	1246	1.18				
CHdiol-T	-		10.1	9.8	3%	32%	white powder				n.o.	n.o.	n.o.	
CHdiol-T	Ti(IV)butoxide	1.0%	9.7	9.7	0%	32%	white powder	1100	1380	1.25				
CHdiol-T	Ti(IV)butoxide	1.0%	10.4	10.3	1%	32%	white powder	1098	1352	1.23				
CHdiol-T	Ti(IV)butoxide	1.0%	10.6	10.4	2%	32%	white powder				n.o.	n.o.	n.o.	
CHdiol-T	Sn(II)Octanoate	1.0%	9.8	9.8	0%	32%	white powder	1128	1430	1.27				
CHdiol-T	Sn(II)Octanoate	1.0%	10.6	10.6	0%	32%	white powder	1140	1464	1.28				
CHdiol-T	Sn(II)Octanoate	1.0%	9.9	9.9	0%	32%	white powder				n.o.	n.o.	n.o.	
CHdiol-A	-		10.4	5.7	45%	34%	light brown	1147	1457	1.27				
CHdiol-A	-		10.3	5.8	44%	34%	light brown	1147	1442	1.26				
CHdiol-A	-		10.6	6.0	43%	34%	light brown				n.o.	n.o.	72.19	
CHdiol-A	Ti(IV)butoxide	1.0%	9.5	7.4	22%	34%	clear yellow	10542	28049	2.66				
CHdiol-A	Ti(IV)butoxide	1.0%	10.0	7.3	27%	34%	clear yellow	15957	45639	2.86				
CHdiol-A	Ti(IV)butoxide	1.0%	9.8	7.3	26%	34%	clear yellow				4.61	116.81		61.63
CHdiol-A	Sn(II)Octanoate	1.0%	9.7	6.8	30%	34%	clear	7654	17998	2.35				
CHdiol-A	Sn(II)Octanoate	1.0%	9.6	7.0	27%	34%	clear	8101	19015	2.35				
CHdiol-A	Sn(II)Octanoate	1.0%	10.1	7.6	25%	34%	clear				4.98	117.85	79.25	
CHdiol-S	-		9.8	5.9	40%	29%	opaque	1138	1439	1.26				
CHdiol-S	-		10.3	6.2	40%	29%	opaque	1124	1433	1.27				
CHdiol-S	-		10.4	6.1	41%	29%	opaque				-38.03	37.54	27.83	
CHdiol-S	Ti(IV)butoxide	1.0%	10.2	7.6	25%	29%	clear	15232	46792	3.07				
CHdiol-S	Ti(IV)butoxide	1.0%	10.5	8.1	23%	29%	clear	16651	44504	2.67				
CHdiol-S	Ti(IV)butoxide	1.0%	10.2	8.1	21%	29%	clear				-22.05	75.04		8.21
CHdiol-S	Sn(II)Octanoate	1.0%	10.5	7.6	28%	29%	opaque	4937	10392	2.10				
CHdiol-S	Sn(II)Octanoate	1.0%	9.8	7.4	24%	29%	opaque	5740	11025	1.92				
CHdiol-S	Sn(II)Octanoate	1.0%	10.1	7.9	22%	29%	opaque				-24.24	81.63	30.18	1.46

Table 7. Details of polymerization with 1,4-cyclohexanedimethanol in the small-scale reactor.

monomer	Cat	cat (mol%)	Weight input	Weight after rxn	weight loss %	theoretical weight loss	color	M_n	M_w	Đ	T_g (°C)	T_m (°C)	T_c (°C)	T_{cc} (°C)
CHdimeoh-T	-		10.0	9.1	9%	34%	white, part melted	1072	1572	1.47				
CHdimeoh-T	-		10.0	9.0	10%	34%	white, part melted	1069	1557	1.46				
CHdimeoh-T	-		10.5	9.5	10%	34%	white, part melted				n.o.	147.21	124.87	
CHdimeoh-T	Ti(IV)butoxide	1.0%	9.8	8.0	18%	34%	white, part melted	3814	6615	1.73				
CHdimeoh-T	Ti(IV)butoxide	1.0%	10.2	8.4	18%	34%	white, part melted	3812	6644	1.74				
CHdimeoh-T	Ti(IV)butoxide	1.0%	9.6	7.9	18%	34%	white, part melted				n.o.	n.o.	n.o.	
CHdimeoh-T	Sn(II)Octanoate	1.0%	10.4	8.3	20%	34%	white, part melted	4084	7181	1.76				
CHdimeoh-T	Sn(II)Octanoate	1.0%	10.4	8.4	19%	34%	white, part melted	4065	7083	1.74				
CHdimeoh-T	Sn(II)Octanoate	1.0%	10.0	8.3	17%	34%	white, part melted				n.o.	98.03	47.48	
CHdimeoh-A	-		9.2	7.4	20%	36%	opaque	1736	2658	1.53				
CHdimeoh-A	-		10.5	8.3	21%	36%	opaque	1720	2587	1.50				
CHdimeoh-A	-		10.2	8.3	19%	36%	opaque				n.o.	81.82	30.23	
CHdimeoh-A	Ti(IV)butoxide	1.0%	10.0	7.7	23%	36%	yellow	11597	28016	2.42				
CHdimeoh-A	Ti(IV)butoxide	1.0%	10.0	7.6	24%	36%	yellow	12018	27542	2.29				
CHdimeoh-A	Ti(IV)butoxide	1.0%	9.8	7.6	22%	36%	yellow				n.o.	98.03	47.96	
CHdimeoh-A	Sn(II)Octanoate	1.0%	10.5	8.3	21%	36%	opaque	12039	26910	2.24				
CHdimeoh-A	Sn(II)Octanoate	1.0%	9.4	7.4	21%	36%	opaque	11897	27617	2.32				
CHdimeoh-A	Sn(II)Octanoate	1.0%	10.2	8.1	21%	36%	opaque				n.o.	92.47	31.58	
CHdimeoh-S	-		10.3	8.7	16%	32%	opaque	2444	4077	1.67				
CHdimeoh-S	-		10.3	8.6	17%	32%	opaque	2419	4008	1.66				
CHdimeoh-S	-		9.4	8.0	15%	32%	opaque				n.o.	41.66	13.83	
CHdimeoh-S	Ti(IV)butoxide	1.0%	9.3	6.8	27%	32%	opaque	13533	34679	2.56				
CHdimeoh-S	Ti(IV)butoxide	1.0%	10.2	7.5	26%	32%	opaque	24481	59284	2.42				
CHdimeoh-S	Ti(IV)butoxide	1.0%	9.8	7.1	28%	32%	opaque				n.o.	48.35	-2.12	
CHdimeoh-S	Sn(II)Octanoate	1.0%	10.2	8.7	15%	32%	opaque	11587	26096	2.25				
CHdimeoh-S	Sn(II)Octanoate	1.0%	9.8	8.2	16%	32%	opaque	12446	26426	2.12				
CHdimeoh-S	Sn(II)Octanoate	1.0%	9.7	8.0	18%	32%	opaque				-38.21	44.08		-13.51

MALDI-ToF-MS

MALDI-ToF-MS expected linear chains

The expected linear distributions for poly(1,3-CP-F) are cyclics (not shown) and linear diol terminated chains (SI_**01**) (Figure 37). Thermal dehydration leads to loss of hydroxyl groups (SI_**02**, and SI_**03**). Further degradation can lead to loss of CPol group (SI_**04**), combined with dehydration (SI_**05**), eventually leading to di-acid terminated chains (SI_**06**).

SI_01

SI_02

SI_03

SI-04

SI_05

SI_06

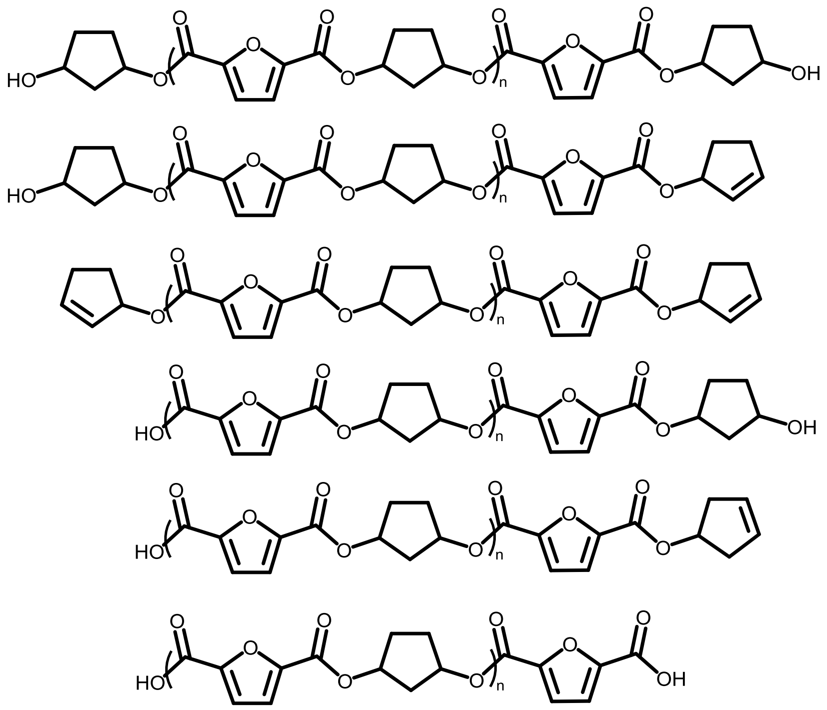

Figure 37. Expected linear distributions of poly(1,3-CP-F): cyclic (not shown), linear diol (SI_01), dehydrated (SI_02,SI_ 03), loss of CPol and dehydrated (SI_04, SI_05, SI_06).

Table 8. Calculated m/z of linear chains depicted in Figure 37, with the weight of a potassium ion.

#	m/z n=1 + K^+	m/z n=2 + K^+	m/z n=3 + K^+	m/z n=4 + K^+
cyclic	482,49	704,69	926,89	1149,09
SI_01	584,62	806,82	1029,02	1251,22
SI_02	566,60	788,80	1011,00	1233,20
SI_03	548,59	770,79	992,99	1215,19
SI_04	500,50	722,70	944,90	1167,10
SI_05	482,49	704,69	926,89	1149,09
SI_06	416,38	638,58	860,78	1082,98

MALDI-ToF-MS - side reactions with cyclopentene group

One expected side reaction is the reaction between two cyclopentene groups, which are generated by the thermal dehydration of pendant cyclopentanol groups. These double bonds can undergo (thermal) radical addition reactions, forming a Cp-Cp bond (Figure 38). The expected structures are diol terminated (SI_**07**), and dehydrated (SI_**08**, SI_**09**). Further degradation can lead to loss of CPol group (SI_**10**), combined with dehydration (SI_**11**), eventually leading to di-acid terminated chains (SI_**12**).

SI_07

SI_08

SI_09

SI_10

SI_11

SI_12

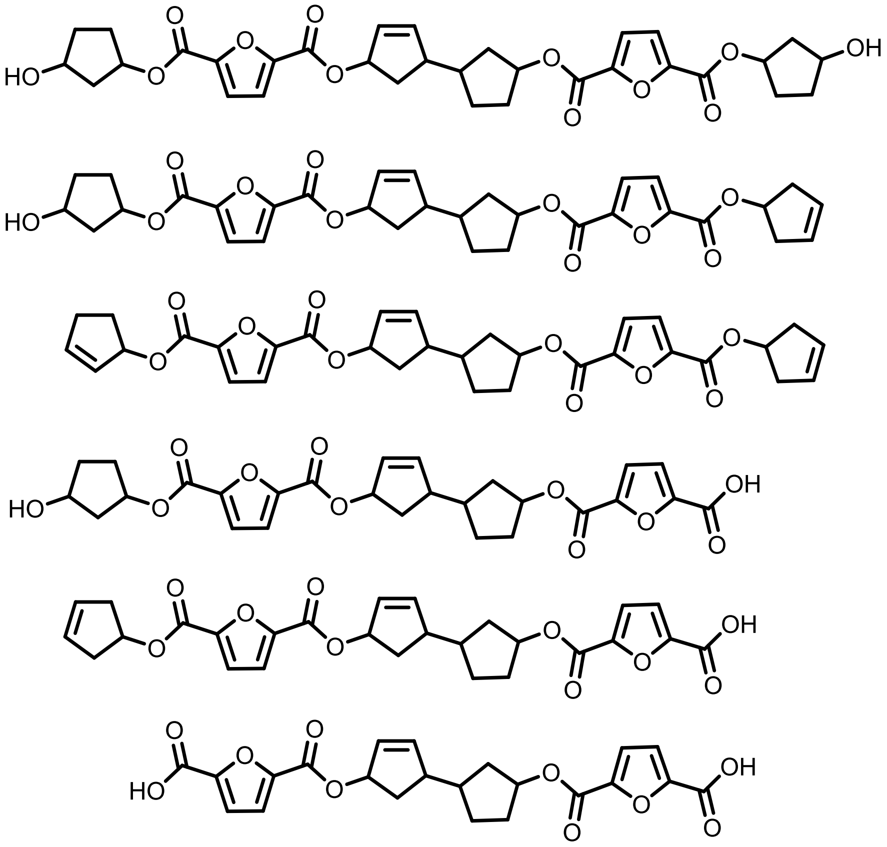

Figure 38. Expected linear distributions after CPene-CPene addition reaction.

Table 9. Calculated m/z of linear chains after Cp-Cp addition reaction depicted in Figure 38, with the weight of a potassium ion.

**#**	**m/z n=1 + K^+**	**m/z n=2 + K^+**	**m/z n=3 + K^+**	**m/z n=4 + K^+**
SI_07	650,72	872,92	1095,12	1317,32
SI_08	632,7	854,90	1077,10	1299,3
SI_09	614,69	836,89	1058,09	1281,29
SI_10	566,60	788,80	1011,0	1233,20
SI_11	548,59	770,79	992,99	1215,19
SI_12	482,48	704,68	926,88	1149,08

MALDI-ToF-MS - other possible side reactions

Two possible Diels-Alders 2+4 cycloaddition reactions are possible between the furan-group and the generated cyclopentene group. The first one (Figure 39, top) is the reaction of cyclopentene with a normal furan group of FDCA. The second one (Figure 39, bottom) is the reaction of cyclopentene with a decarboxylated furan group. The decarboxylation of FDCA is a well-known side reaction, and free acid groups on the furan ring are generated by the degradation mechanism described in this work. Furthermore. Diels-Alder reactions are also known to occur with these furan groups. A trace of SI_15 has been found in the samples with 30% cis 1,3-CPdiol in the polymer.

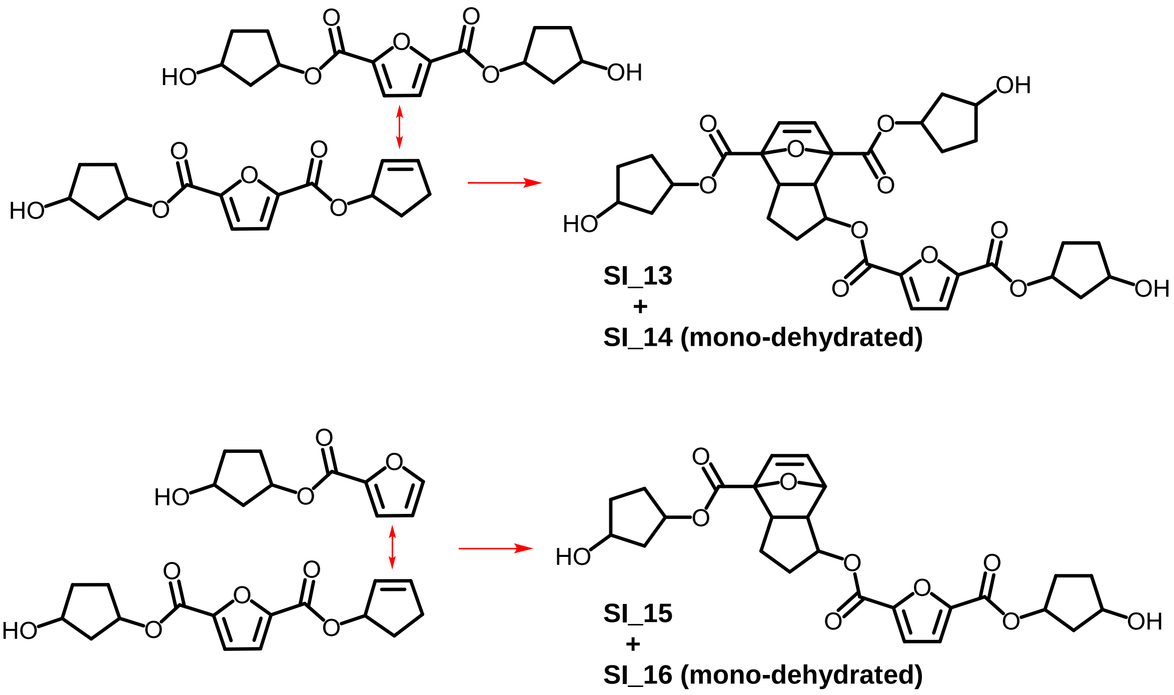

Figure 39. Possible occurring Dials-Alder side reaction between the furan group and the cyclopentene group.

Table 10. Calculated m/z of linear chains after Cp-Cp addition reaction depicted in Figure 39, with the weight of a potassium ion.

#	m/z n=0 + K^+	m/z n=1 + K^+	m/z n=2 + K^+
SI_13	668,73	890,83	1113,13
SI_14	650,73	872,93	1095,13
SI_15	540,61	762,81	985,01
SI_16	522,59	744,79	966,99

Obtained MALDI-ToF-MS spectra 8% cis

In the following MALDI-ToF-MS spectra, and the text, the distributions are numbered in accordance to the structures in Figure 37 and Figure 38, *e.g.* linear diol-terminated chains SI_01, is denoted as **1**. The major ticks are aligned to the *M*0 of the repeat unit CP-F (m/z 222.1), ranging from n=2 to n=6 of the linear diol-terminated chains **1**.

MALDI-ToF-MS spectra obtained for poly(CP-F) with 8% cis, after thermal stability experiments at 180 ºC, 200 ºC and 220 ºC (Figure 40). For the sample at 180 ºC only one distribution of linear diol terminated chains (**1**) are obtained. At 200 ºC more distributions appear: dehydrated linear chains **2**, Cp-Cp addition **7**, and linear ene-acid **5**. At 220 ºC distribution further linear dehydrated **3** appears, distribution **5** disappears, and **7** is dehydrated to **8**.

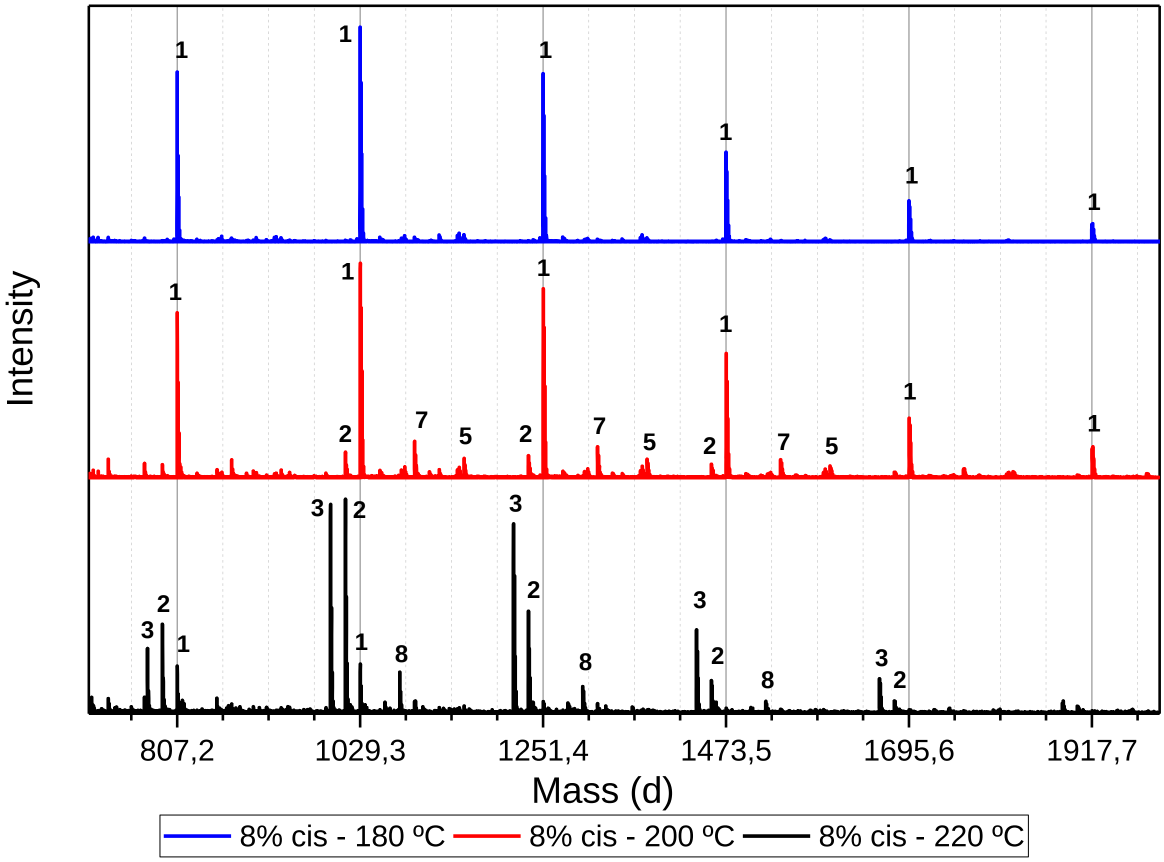

Figure 40. Obtained MALDI-ToF-MS spectra for poly(1,3-CP-F) with 8% cis, after thermal stability experiments at 180 ºC (blue, top), 200 ºC (red, middle), and 220 ºC (black, bottom).

Obtained MALDI-ToF-MS spectra 20% cis

MALDI-ToF-MS spectra obtained for poly(CP-F) with 20% cis, after thermal stability experiments at 180 ºC, 200 ºC and 220 ºC (Figure 41). For the sample at 180 ºC two distributions are found equally present: **1** linear diol terminated chains **1**, and linear di-acid terminated chains **6**. The third distribution is linear ene-acid terminated chains **5**. At 200 ºC mostly linear diol terminated chains **1** are present, together with dehydrated linear chain **2,** and linear di-acid terminated chain **6**. At 220 ºC, linear diol terminated chains **1** disappears, with the appearance of dehydrated linear chains **2**, and **3**. Linear di-acid terminated chain **6** is almost gone, and Cp-Cp addition chains **7**, and the dehydration **8** and **9** are appearing. Please note that **6** and **8** are close, but have a mass-difference of 4 dalton.

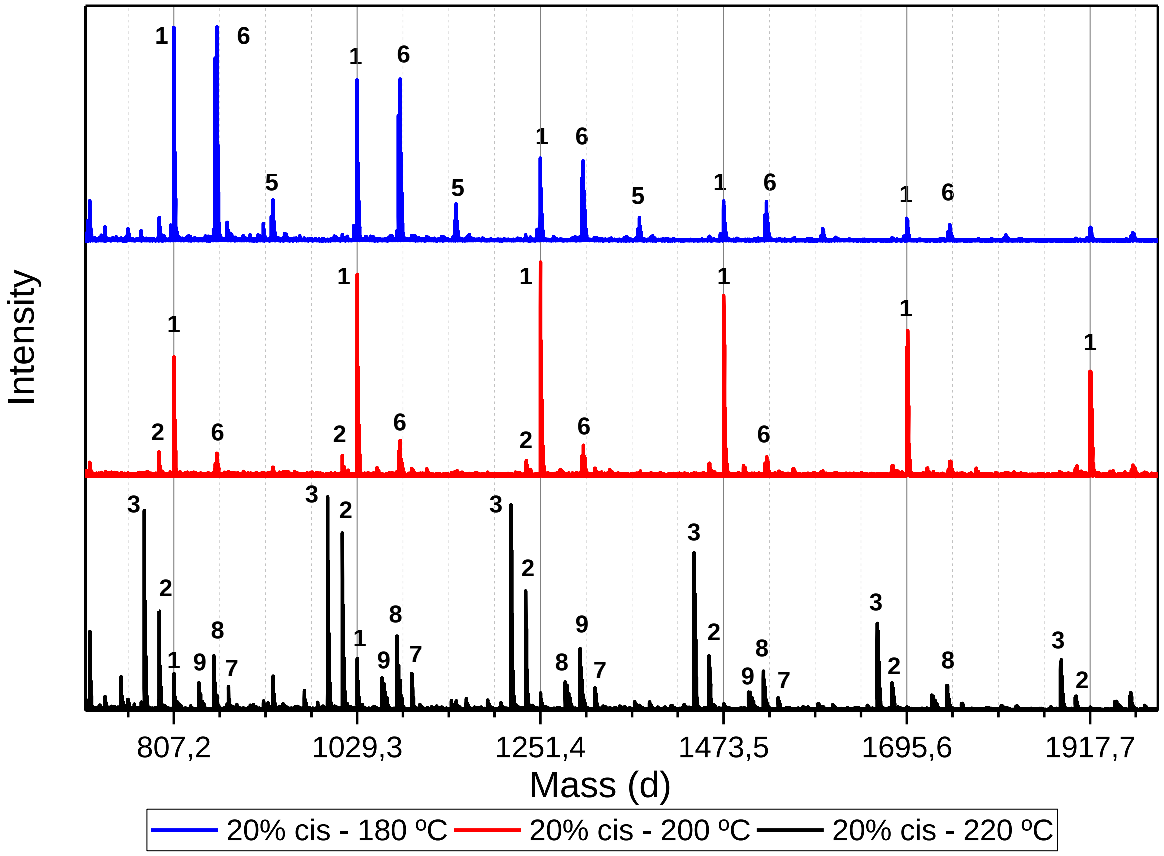

Figure 41. Obtained MALDI-ToF-MS spectra for poly(1,3-CP-F) with 20% cis, after thermal stability experiments at 180 ºC (blue, top), 200 ºC (red, middle), and 220 ºC (black, bottom).

Obtained MALDI-ToF-MS spectra 30% cis

MALDI-ToF-MS spectra obtained for poly(CP-F) with 30% cis, after thermal stability experiments at 180 ºC, 200 ºC and 220 ºC (Figure 42). For the sample at 180 ºC two main distributions are found: linear diol terminated chains **1**, and linear di-acid terminated chains **6**. The third distribution is linear ene-acid terminated chains **5**. At 200 ºC the linear di-acid terminated chain **6** is most abundant, together with linear diol terminated chains **1**, and dehydrated linear chain **2**. The fourth distribution is linear ene-acid terminated chains **5**. At 220 ºC, linear diol terminated chains **1** disappears, with the appearance of dehydrated linear chains **2**, and **3**. Linear di-acid terminated chain **6** is almost gone, but Cp-Cp addition chains **7**, and the dehydration **8** and **9** are appearing.

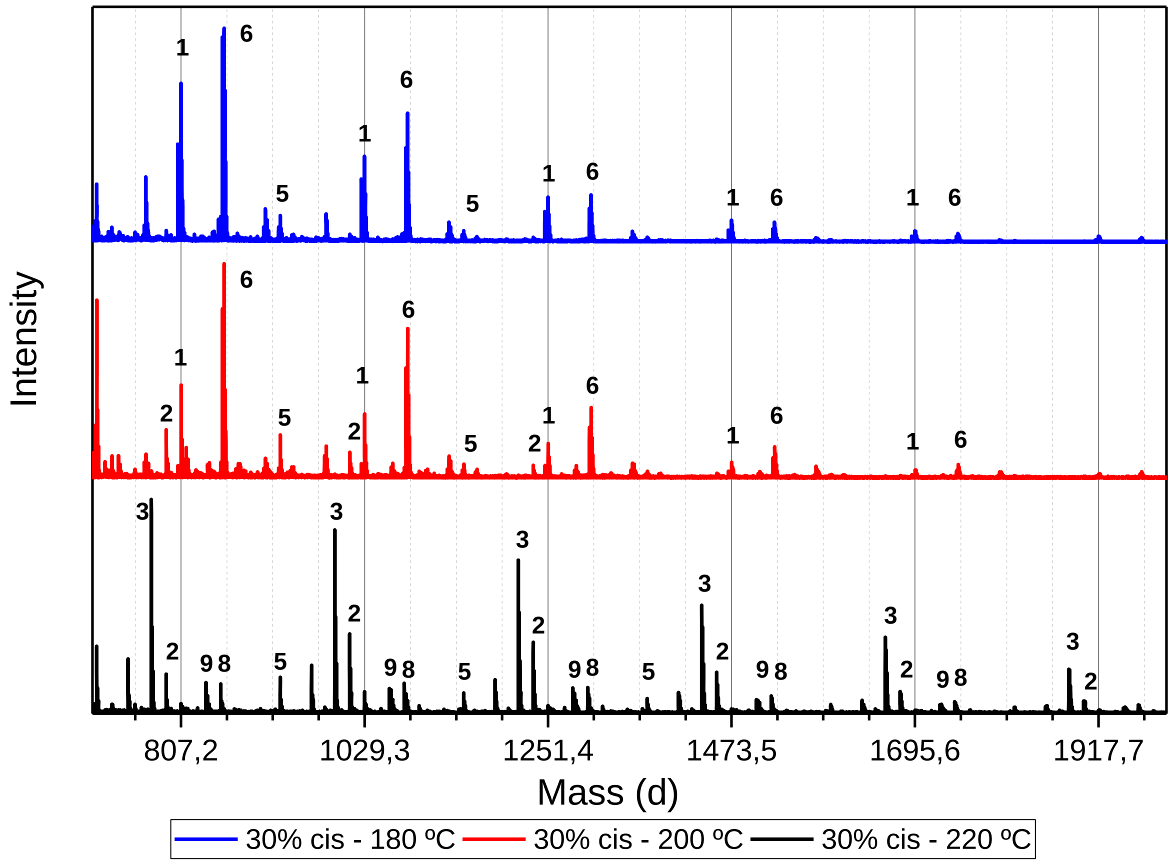

Figure 42. Obtained MALDI-ToF-MS spectra for poly(1,3-CP-F) with 30% cis, after thermal stability experiments at 180 ºC (blue, top), 200 ºC (red, middle), and 220 ºC (black, bottom).

Obtained MALDI-ToF-MS spectra 40% cis

MALDI-ToF-MS spectra obtained for poly(CP-F) with 40% cis, after thermal stability experiments at 180 ºC, 200 ºC and 220 ºC (Figure 43). For the sample at 180 ºC two distributions are found: linear diol terminated chains **1**, and linear di-acid terminated chains **6**. At 200 ºC the linear di-acid terminated chain **6** is most abundant, together with linear diol terminated chains **1**, and dehydrated linear chain **2**. The fourth distribution is linear ene-acid terminated chains **5**. At 220 ºC, linear dehydrated chains **3** are most abundant. Dehydrated Cp-Cp adducts **8** and **9** have appeared alongside linear ene-acid terminated chains **5**.

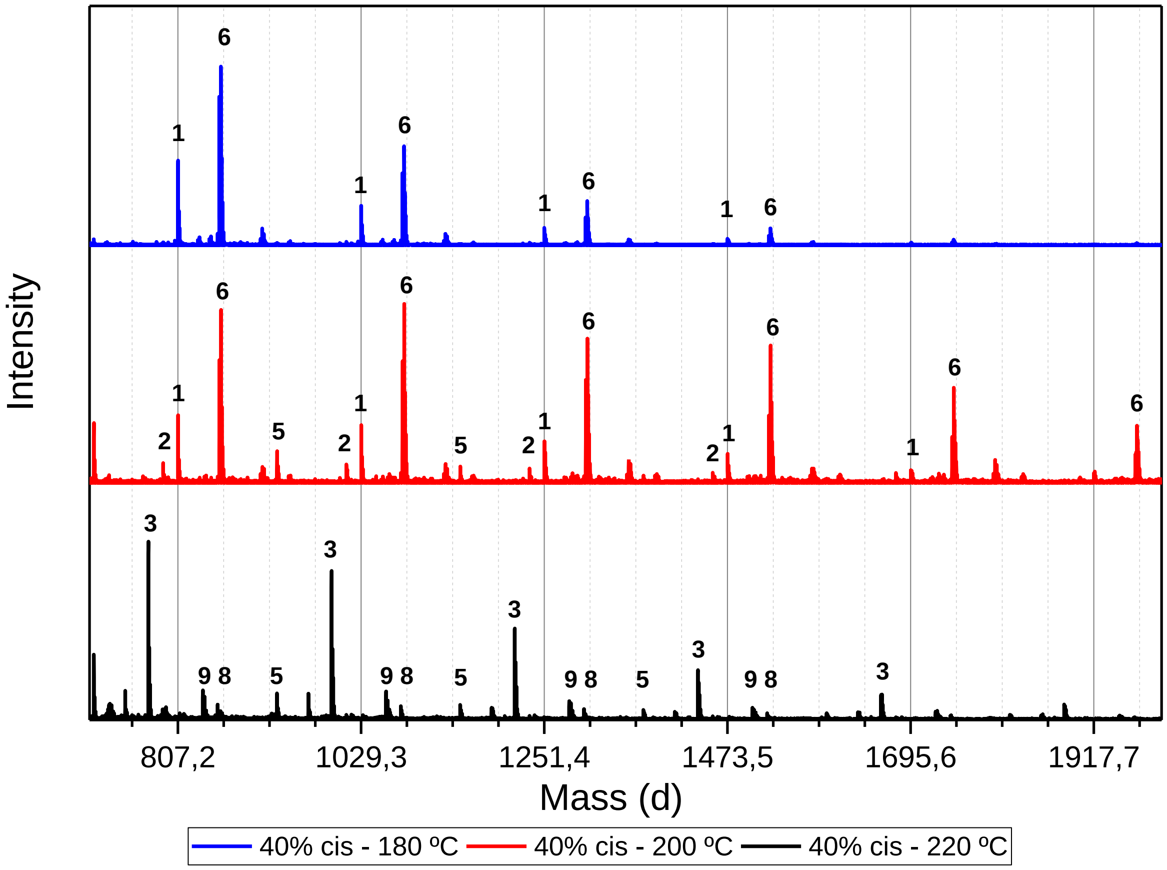

Figure 43. Obtained MALDI-ToF-MS spectra for poly(1,3-CP-F) with 40% cis, after thermal stability experiments at 180 ºC (blue, top), 200 ºC (red, middle), and 220 ºC (black, bottom).
